# Synthesis of Bisphenol Neolignans Inspired by Honokiol as Antiproliferative Agents

**DOI:** 10.3390/molecules25030733

**Published:** 2020-02-07

**Authors:** Nunzio Cardullo, Vincenza Barresi, Vera Muccilli, Giorgia Spampinato, Morgana D’Amico, Daniele Filippo Condorelli, Corrado Tringali

**Affiliations:** 1 Department of Chemical Sciences, University of Catania, Viale A. Doria 6, 95125 Catania, Italy; v.muccilli@unict.it; 2 Department of Biomedical and Biotechnological Sciences, Section of Medical Biochemistry, University of Catania, Via Santa Sofia 97, 95123 Catania, Italy; vincenza.barresi@unict.it (V.B.); giorgiaspampinato@unict.it (G.S.); morganadamico01@gmail.com (M.D.); daniele.condorelli@unict.it (D.F.C.)

**Keywords:** honokiol, bisphenol neolignans, polyphenols, Suzuki–Miyaura cross-coupling, antitumor activity, apoptosis

## Abstract

Honokiol (2) is a natural bisphenol neolignan showing a variety of biological properties, including antitumor activity. Some studies pointed out 2 as a potential anticancer agent in view of its antiproliferative and pro-apoptotic activity towards tumor cells. As a further contribution to these studies, we report here the synthesis of a small library of bisphenol neolignans inspired by honokiol and the evaluation of their antiproliferative activity. The natural lead was hence subjected to simple chemical modifications to obtain the derivatives 3–9; further neolignans (12a-c, 13a-c, 14a-c, and 15a) were synthesized employing the Suzuki–Miyaura reaction, thus obtaining bisphenols with a substitution pattern different from honokiol. These compounds and the natural lead were subjected to antiproliferative assay towards HCT-116, HT-29, and PC3 tumor cell lines. Six of the neolignans show GI_50_ values lower than those of 2 towards all cell lines. Compounds 14a, 14c, and 15a are the most effective antiproliferative agents, with GI_50_ in the range of 3.6–19.1 µM, in some cases it is lower than those of the anticancer drug 5-fluorouracil. Flow cytometry experiments performed on these neolignans showed that the inhibition of proliferation is mainly due to an apoptotic process. These results indicate that the structural modification of honokiol may open the way to obtaining antitumor neolignans more potent than the natural lead.

## 1. Introduction

**Figure 1:**
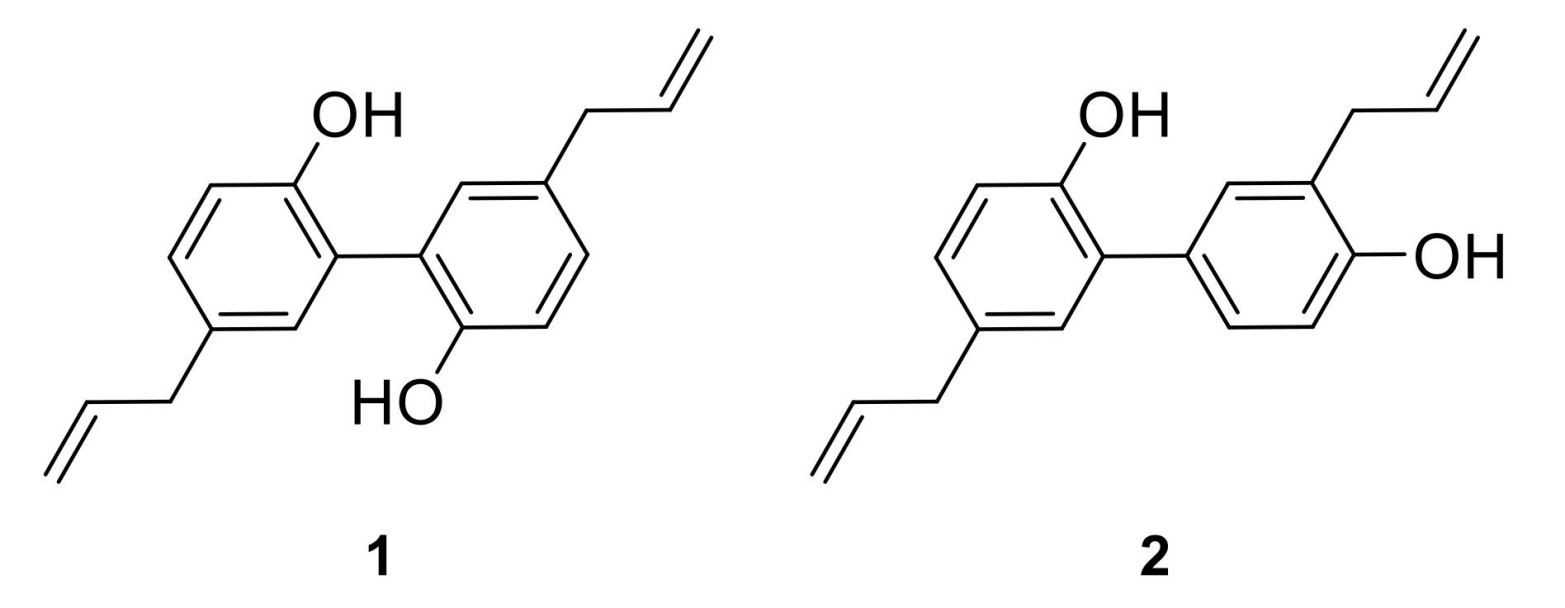
Chemical structures of magnolol (1) and honokiol (2).

The biaryl skeleton is relatively common among natural products and this structural feature is distinctive of bisphenol neolignans, a group of polyphenols belonging to the neolignan family [1]. These compounds are biosynthesized through oxidative coupling of phenoxy radicals generated by enzymes such as laccase, peroxidase, or a cytochrome P450 [2]. The most representative bisphenol neolignans are magnolol (1, Figure 1) and honokiol (2, Figure 1), originally isolated from roots and stem bark of *Magnolia officinalis* and *Magnolia obovata*. The extracts of *Magnolia* spp. (mainly *M. officinalis*) have been employed for centuries in traditional Chinese and Japanese medicine to treat many diseases, including anxiety, allergy, or gastrointestinal disorders [3,4]. These extracts have shown to possess promising biological activities, including anti-inflammatory, antioxidant, antiviral, anti-depressant, and anti-platelet activity [4,5]. 

Magnolol and honokiol are the main bioactive ingredients of these extracts [6] and have shown an array of biological properties, including antioxidant [6,7], anti-inflammatory [8], neuroprotective [9], and antitumor activity [10,11]. Specifically, 1 and 2 inhibit proliferation of tumor cells, inducing differentiation and apoptosis, and suppressing angiogenesis [12,13,14,15]. Furthermore, their unique pharmacophore structure, that is two phenolic rings linked through a C–C bond allows the interaction with a variety of biological targets [16].

The above-cited properties have prompted many researchers to synthesize magnolol and honokiol analogues and evaluate their biological properties to obtain new potential therapeutic agents. These efforts have afforded new bisphenol neolignans with optimized properties, among which antimicrobial [17,18], neuroprotective [19], anti-inflammatory [20], antitumor [18,21,22], and antiangiogenic activity [23]. According to some studies, the antitumor activity of honokiol, magnolol, and their analogues is related to the presence of free hydroxyl group and allylic chains on a bisphenolic moiety [18,23].

Although several synthetic methods have been employed to obtain biaryl compounds, the Pd-catalyzed Suzuki–Miyaura (S–M) cross-coupling reaction is one of the most efficient [24,25]. Moreover, with respect to other Pd-catalyzed reactions, S–M coupling has the advantage of requiring mild conditions and employing commercially available boronic acids that are environmentally safer than organometallic reagents [24]. On the other hand, oxidative coupling methods, based on the use of enzymes such as horseradish peroxidase [26], allow the synthesis of bisphenol neolignans in eco-friendly conditions but provide moderate or poor yield.

Thus, in continuation of our previous studies on the synthesis of natural-derived polyphenols with antitumor [27,28,29,30,31], antioxidant, hypoglycemic [32,33], antifungal [34], and anti-inflammatory activity [35,36], we oriented our recent works toward the synthesis of magnolol analogues, which were evaluated as potential antidiabetic [26], anticancer [37], and antioxidative [38] agents. As a further contribution, in the present work we report the synthesis of bisphenols neolignans inspired by honokiol (2). All the synthetic neolignans, in comparison with 2, have been evaluated for their potential antiproliferative activity towards three tumor cell lines, (HCT-116, HT-29 and PC3).

## 2. Results and Discussion

### 2.1. Synthesis

**Scheme 1:**
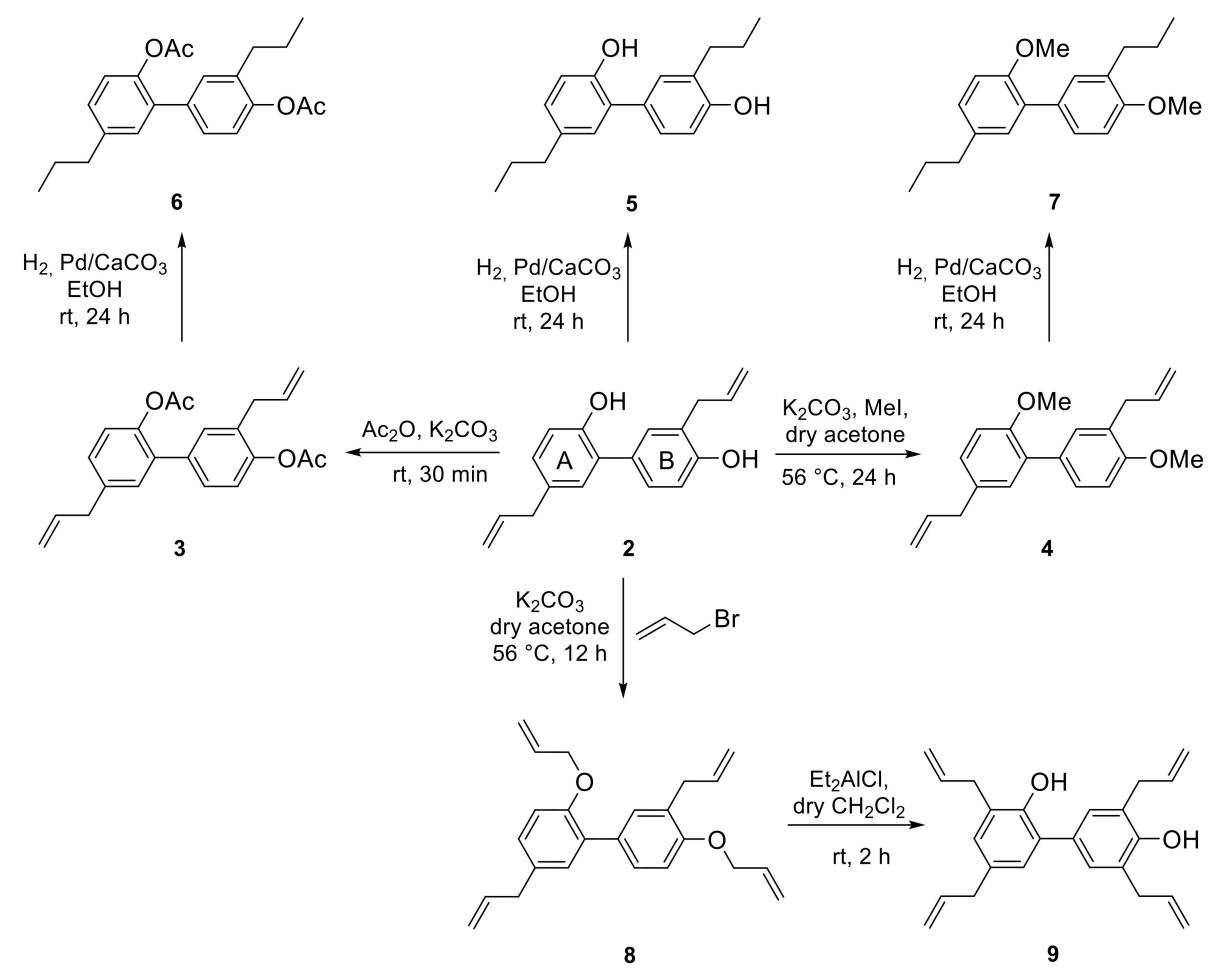
Synthesis of honokiol derivatives 3–9.

On the basis of the above-cited biological properties of the natural lead 2, we planned to synthesize a small library of honokiol-inspired bisphenol neolignans. A first group of honokiol analogues was obtained through simple modifications of 2, as depicted in Scheme 1. By acetylation and methylation, we obtained compounds 3 and 4, respectively. Peracetate derivatives usually undergo in vivo enzymatic hydrolysis and are frequently prepared to overcome the low metabolic stability and poor bioavailability of natural polyphenols [29,39], whereas methylated analogues of polyphenols have shown in many cases enhanced biological activity and high metabolic stability [39,40]. The neolignans 2, 3, and 4 were subjected to catalytic hydrogenation to give respectively 5, 6, and 7, as it is useful to establish the possible role of the terminal double bond. The spectroscopic data of compounds 3–5, and 7 were in agreement with those previously reported in the literature [41,42], whereas the new bisphenol neolignan 6 was subjected to spectroscopic characterization and the analysis of HRMS, ^1^H and ^13^C-NMR spectra confirmed the expected structure.

According to the above cited report [18], the allylic chains on the bisphenolic core of honokiol are important structural requirements for antiproliferative activity; thus, we planned to investigate the effect of further allylic or *O*-allylic substituents. Namely, 2 was subjected to S_N_2 reaction with allyl bromide to obtain the bis-*O*-allyl honokiol 8, whose structure was confirmed by analysis of its ^1^H and ^13^C-NMR data, in agreement with those previously reported [20]. As a further step, the Claisen rearrangement of 8 was planned to obtain the bis-*C*-allyl derivative 9. This reaction was carried out in mild conditions, namely at room temperature and in the presence of Et_2_AlCl which catalyzes the [3,3]-sigmatropic rearrangement via an ether–aluminum complex, avoiding the use of high temperature. The analysis of ^1^H and ^13^C-NMR data, in agreement with those reported in literature, confirmed the structure of 9 [43]. 

**Scheme 2:**
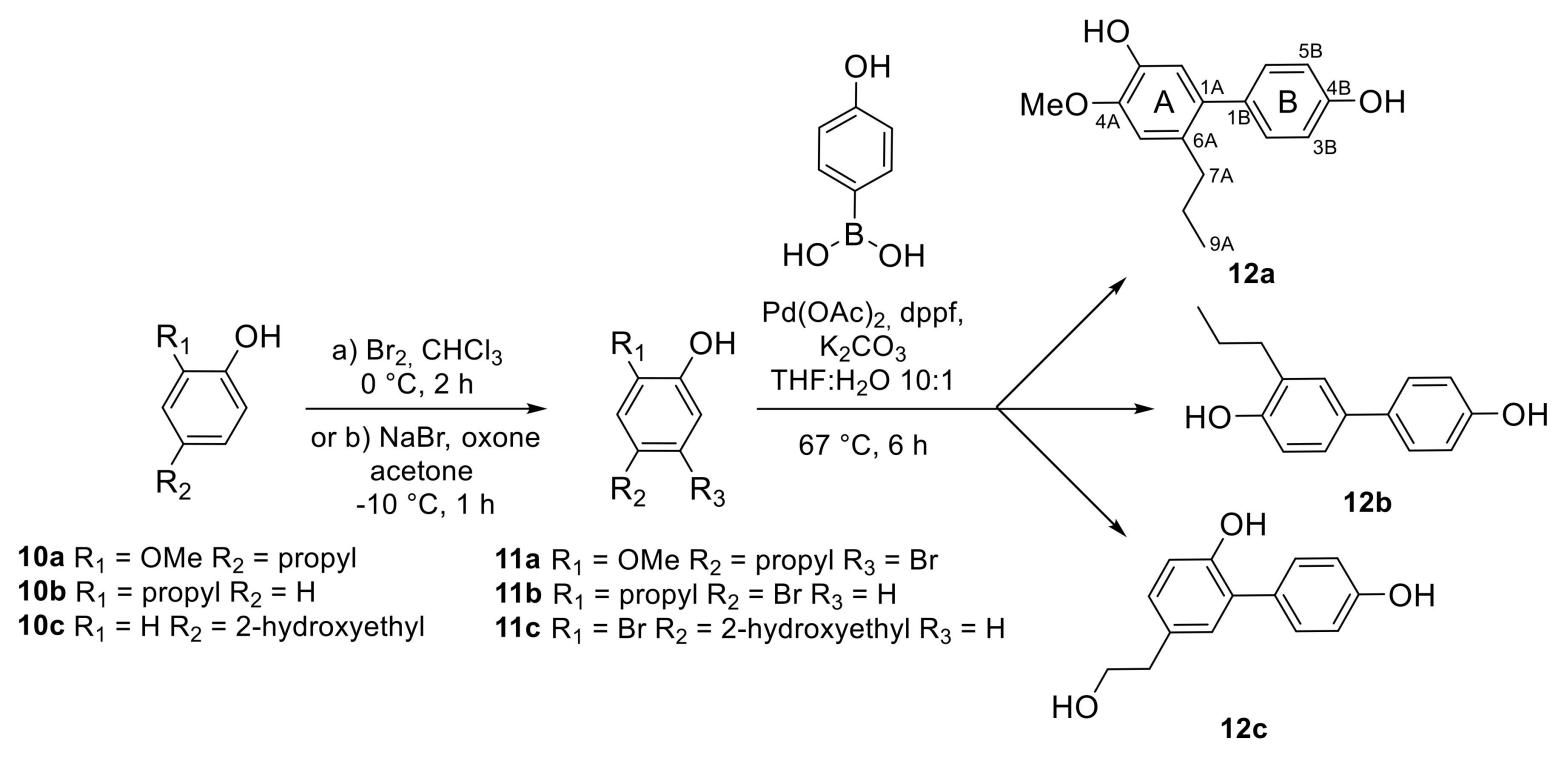
Synthesis of bisphenol neolignans 12a-c. (**a**) These conditions were employed to obtain 11a and 11b; (**b**) these conditions were employed to obtain 11c.

**Scheme 3:**
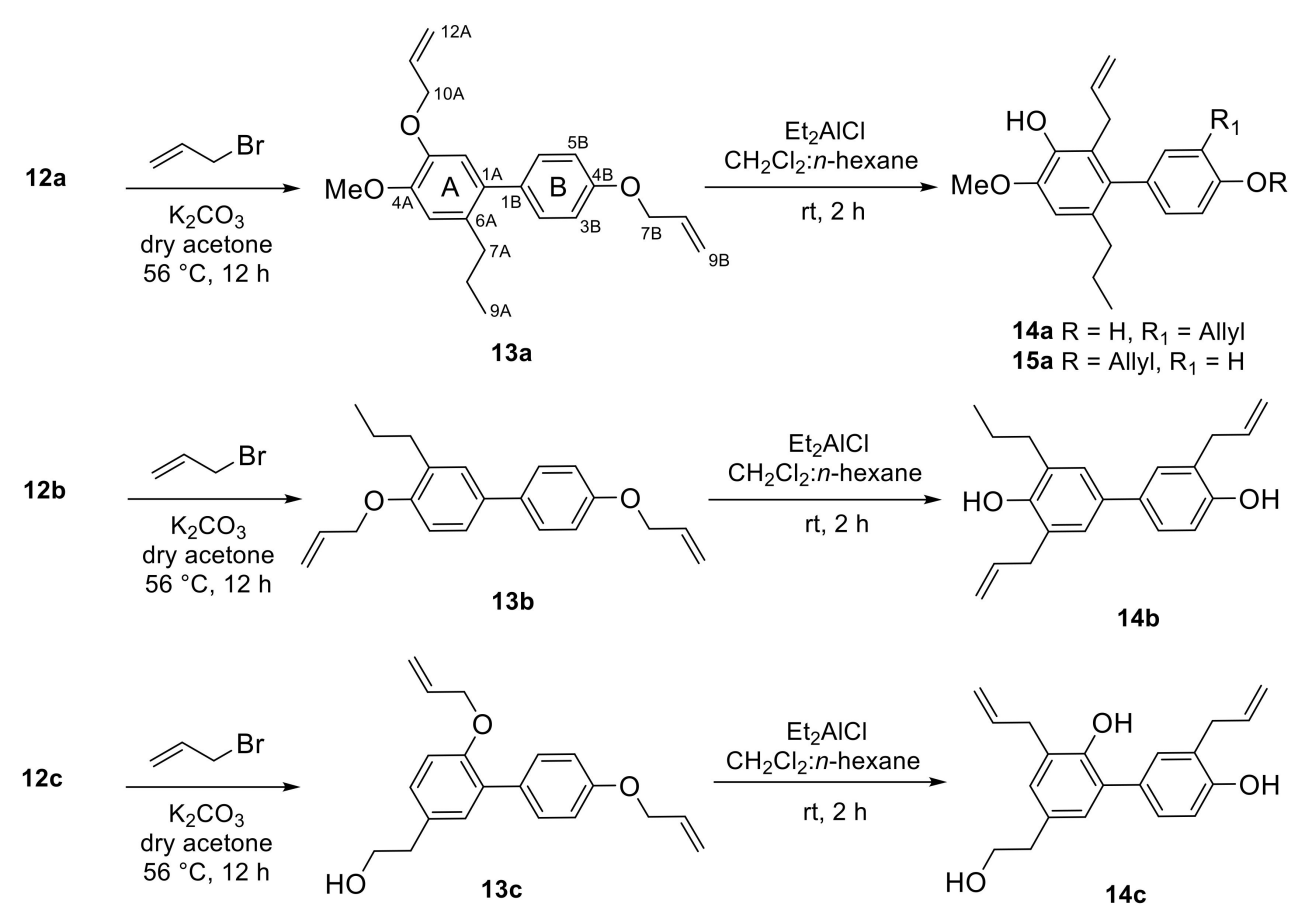
Synthesis of bisphenol neolignans 13a-c, 14a-c, and 15a.

Another set of bisphenol neolignans has been synthesized starting from commercial phenolics and employing the synthetic strategy reported in Scheme 2 and Scheme 3 based on the Suzuki–Miyaura cross-coupling reaction; accordingly, bisphenols 12a-c, 13a-c, 14a-c, and 15a were obtained.

Scheme 2 and Scheme 3 summarize the final reaction conditions employed for each step, these were then optimized through a series of preliminary reactions. More specifically, the substrate 10a was used to optimize both the bromination and the S–M reactions. Preliminary experiments for the bromination were carried out employing three different brominating agents (namely, Br_2_, *N*-bromosuccinimide, and NaBr/oxone) with or without a catalyst (AlCl_3_ or I_2_) and testing different solvents (CH_3_CN, CHCl_3_, and acetone). The reaction mixtures were analyzed by HPLC-UV on a C18 reversed-phase column in order to quantify the yield of the product 11a. These experiments are reported in detail in the experimental section and the results are summarized in Table 1.

When the substrate 10a was treated with Br_2_ (entry 6) the expected monobromo derivative was obtained with higher yield (47%) respect to when the reaction occurred in other conditions. Furthermore, by working at 0 °C (entry 7) the yield grew up to 63%. 

The same methodology was employed for the bromination of 10b, thus obtaining 11b with 67% yield; 10c afforded 11c with a lower yield (36%), hence, we applied the procedure previously described by Bovicelli et al. [44], namely by treating tyrosol (10c) with NaBr and oxone; in contrast with the low yield obtained for 11a (entry 8), 11c was recovered with 78% yield. 

Also for the S–M coupling step a careful analysis of different reaction conditions was performed; the results for the coupling of 11a with 4-hydroxyphenyl boronic acid are summarized in Table 2, reporting the yields for the product 12a, subsequently established as the expected bisphenol neolignan (see below). The reaction was carried out varying solvent or solvent mixtures, the temperature or the bromide concentration (entries 3 and 4); Pd(OAc)_2_ and the ligand 1,1′-bis(diphenylphosphino)ferrocene (dppf) were used to generate in situ the catalyst, and K_2_CO_3_ as base. The yield of neolignan 12a was determined by HPLC-UV analysis of the reaction mixtures. The results clearly indicated the best conditions for this step: a mixture THF/H_2_O as solvent system at 70 °C, with 0.05 M concentration of bromide, affording 12a with 67% yield.

On the basis of these encouraging results, the S–M coupling was carried out in a preparative scale and, after purification, 12a was submitted to a complete characterization by means of HRMS, ^1^H and ^13^C-NMR spectra analysis, including two-dimensional methods (COSY, HSQC, and HMBC). The HRMS spectrum confirmed the formation of a biphenyl structure. The NMR spectra showed the signals of a typical AA′XX′ aromatic spin system assigned to ring B of 12a, namely two proton doublets at δ 7.14 (H-2B/H-6B) and 6.85 (H-3B/H-5B), with corresponding carbon signals at δ 130.7 (C-2B/C-6B) and 115.0 (C-3B/C-5B). Two sp^2^ quaternary carbon signals were assigned at C-4B (δ 154.4) and C-1B (δ 134.4) on the basis of chemical shift and HMBC correlations with H-2B/H-6B and H-3B/H-5B. C-1B also showed a correlation with the singlet at δ 6.78, assigned to H-2A; this signal was HMBC correlated with the carbon at δ 134.6, assigned to C-1A on the basis of further HMBC correlations with H-5A and H-2B/H-6B; C-1A was further correlated with the signal at δ 2.47, evidently due to the H_2_-7A protons of the propyl chain. Overall, these and other HMBC data unambiguously established structure 12a. 

The new bisphenol neolignan 12a was reacted with allyl bromide and afforded the bis-*O*-allyl derivative 13a, whose structure was confirmed by analysis of HRMS, ^1^H and ^13^C-NMR spectra. Namely, the mass spectrum proved that a double substitution occurred. This was of course confirmed by the NMR data clearly showing signals due to two allyl chains; these were distinguished on the basis of the HMBC correlations of C-3A and C-4B with the pertinent methylene signals in the ^1^H-NMR spectrum.

As final step, the allyloxy neolignan 13a was used as substrate for a Claisen rearrangement (carried out as above reported for the preparation of 9) affording two main products. The HRMS and NMR data of the more polar product indicated that both allyl chains underwent the Claisen rearrangement. COSY and HMBC experiments corroborated this assumption, thus establishing structure 14a for this bisphenol neolignan. The less polar product showed ^1^H and ^13^C-NMR signals of ring B and those of one *O*-allyl chain substantially superimposable with those of 13a; the other signals indicated that the rearrangement occurred only for the ring A chain; thus, structure 15a was assigned to this product.

With the same protocol (Scheme 2 and Scheme 3) the new bisphenols 12b and c, 13b and c, and 14b and c were obtained and fully characterized. 

### 2.2. Biochemical Assay

**Figure 2:**
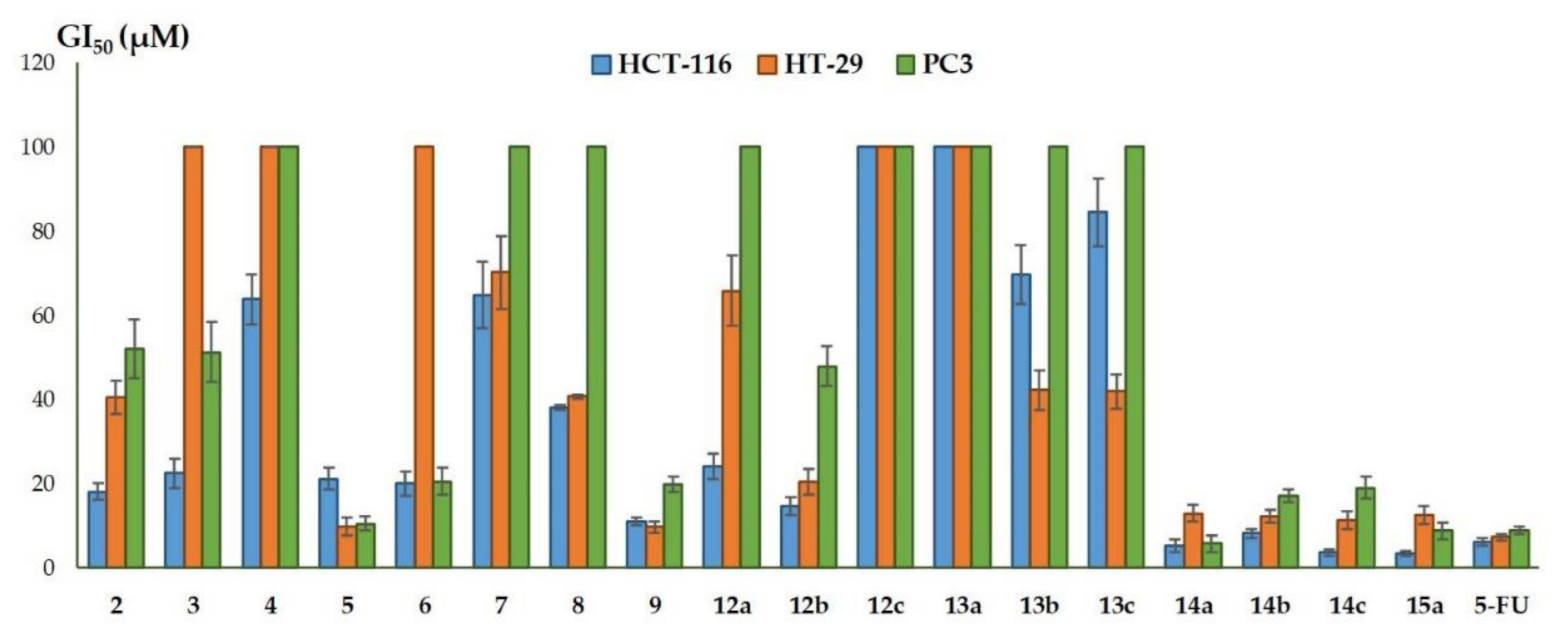
GI50 values (µM) of bisphenol neolignans 2–9, 12a-c, 13a-c, 14a-c, and 15a and of the reference compound 5-fluorouracil (5-FU) on HCT-116, HT-29, and PC3 cell lines after an incubation time of 72 h. The results shown are means ± SD of four experiments.

The honokiol derivatives 3–9 and the bioinspired bisphenol neolignans 12a-c, 13a-c, 14a-c, and 15a were evaluated as potential antiproliferative agents towards three tumor cell lines: HCT-116, HT-29 (both human colorectal adenocarcinoma) and PC3 (human prostate adenocarcinoma) employing the MTT colorimetric assay. The anticancer drug 5-fluorouracil (5-FU) was used as positive control, while honokiol (2) was included in the study for comparison. The results are reported in Table 3 as GI_50_ values (µM) and in Figure 2 for the sake of clarity. The majority of the tested compounds shows, at least on one cell line, a higher activity than that of the lead compound honokiol (GI_50_ = 18.2, 40.6 and 52.1 µM towards HCT-116, HT-29, and PC3 cells, respectively). In particular, six compounds (9, 12b, 14a-c, 15a) show GI_50_ values lower than those of 2 for all cell lines, with GI_50_ values in the range 3.6–47.9 µM. Also 5, the hydrogenated analogue of 2, shows GI_50_ values lower than those of the natural lead for both HT-29 and PC3 cell lines and a comparable value towards HCT-116. Finally, compound 3 is comparable to 2 towards both HCT-116 and PC3 cell lines. These results indicate that the structural modification of honokiol may open the way to obtaining antitumor neolignans more potent than the lead compound.

The bisphenol neolignans 14a, 14c, and 15a gave the most promising results, in particular 14a and 15a showed antiproliferative activity higher or comparable with that of the anticancer drug 5-FU against both HCT-116 and PC3. 

Although the data reported in Table 3 do not allow conclusive assessments about the structural determinants required for an optimized antiproliferative activity of honokiol-inspired neolignans analogues, some considerations about structure-activity relationships can be made and are reported below.

The neolignans 9, 12b, 14a-c, and 15a, with higher antiproliferative activity than 2, possess one allyl or propyl chain in ortho position to a free phenolic group. Among these, 14a, 14b and 14c present the same structural motif of honokiol on ring B. In particular, the presence of free phenolic groups seems to be a pivotal requirement: in fact, compound 4, the dimethyl ether of honokiol, is practically inactive, and the majority of poorly or not active neolignans have no free phenolic groups, with the exception of 12a and 12c, lacking of the allyl chains present in honokiol. Diacetates 3 and 6 show an activity slightly lower than that of 2 towards HCT-116 and PC3 cells, suggesting that these compounds may act as prodrug and may release 2 or the hydrogenated honokiol in presence of intracellular esterases. The above cited structural features are not present in compounds with very low antiproliferative activity. The presence of one or more allyloxy groups does not seem to be, by itself, an essential structural motif, being present in active compounds such as 15a, but also in poor or not active neolignans, such as 8, 13a-c. Finally, it is worthy of note that the hydrogenation of honokiol to give 5 causes an enhancement of activity toward HT-29 and PC3 cells, thus suggesting that these terminal double bonds are not essential for the activity.

**Figure 3:**
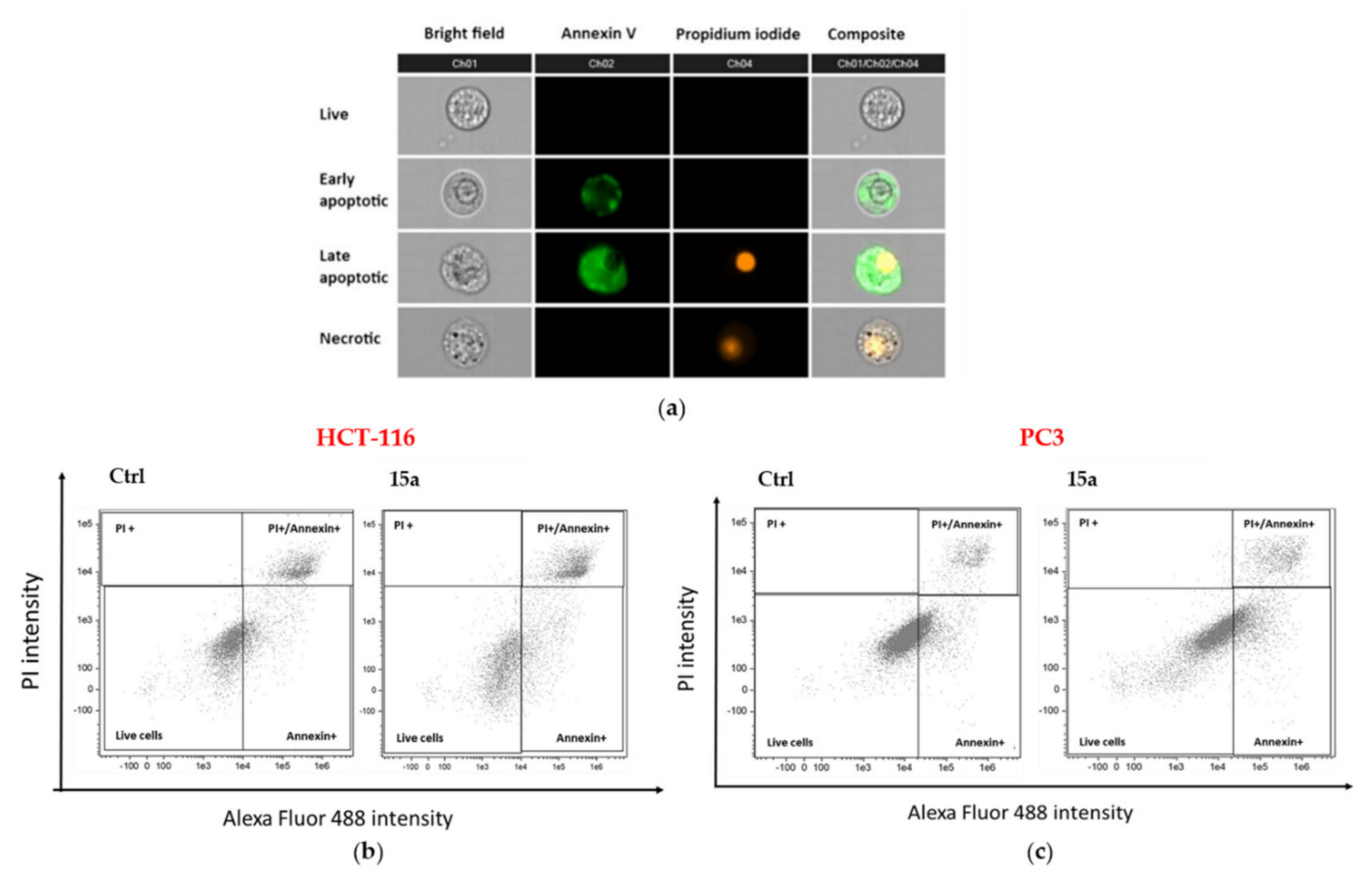
Flow cytometry: (**a**) Typical images of cells analyzed by flow cytometry (Amnis FlowsSigh). Each cell (event) is visible in a bright field and stained by annexin-V positive, propidium iodide positive, and double positive cells; Flow cytometric dot plot of specific cell populations in HCT-116 (**b**) and PC3 (**c**) cell lines in the presence of 15a: live (double annexin/PI negative), necrosis (annexin negative and PI positive), early apoptosis (annexin positive), and late apoptosis (double annexin/PI positive).

On the basis of the above data, we selected three of the most potent neolignans, namely 14a, 14c, and 15a for a flow cytometric analysis on HCT-116 and PC3 cells. This analysis showed that the inhibition of proliferation is mainly due to an apoptotic process, with high values of apoptotic cells in almost all assays (Table 4). Cells treated with 15a showed the highest values of apoptotic cells in both lines: 53.3% of HCT-116 and 38.4% of PC3 were detected in early and late apoptosis status (Figure 3, Table 4). On the contrary, necrotic cells detectable by Propidium Iodide (PI) staining alone were not revealed at significant levels for all tested compounds in both cell lines (Table 4 and Figure 3 for 15a). The results obtained by antiproliferative assay and flow cytometry suggest that selected bioinspired bisphenol neolignans could be further examined in depth in order to define the underlying antitumor mechanism.

## 3. Materials and Methods 

### 3.1. General Information

All chemicals were of reagent grade, and were used without further purification. Honokiol, 4-hydroxyphenylboronic acid, 1,1 ′-bis(diphenylphosphino)ferrocene (dppf), Pd(OAc)_2_ were purchased from TCI Europe (Milan, Italy), eugenol, 2-allyl phenol, tyrosol were purchased from Sigma Aldrich (Milan, Italy). 

Preparative liquid chromatography was performed on silica gel (63-200 µm, Merck, Darmstadt, Germany), or Sephadex-LH20 (Sigma-Aldrich, Milan, Italy) using different mixtures of solvents, as reported for each compound. TLC was carried out using pre-coated silica gel F254 plates (Macherey-Nagel, Düren, Germany); visualization of reaction components was achieved under UV light at wavelengths of 254 and 366 nm, and by staining with a solution of cerium sulfate followed by heating.

HPLC-UV instrument (Agilent, Milan, Italy), equipped with an auto-sampler (G1313A), a pump (G1354A) and a diode array detector (DAD; G1315D), was employed for quantitative analysis with an analytical reversed-phase column (Luna C18, 5 μm; 4.6 × 250 mm; Phenomenex, Castel Maggiore, BO, Italy) and eluting at 1 mL/min with the following gradient of CH_3_CN−HCOOH (99:1 v/v; A) in H_2_O−HCOOH (99:1 v/v; B): t_0_ min A = 50%, t_15_ min A = 100%, t_20_ min A = 50%. 

NMR spectra were run on a Varian Unity Inova spectrometer (Italy, Milan) operating at 499.86 (^1^H) and 125.70 MHz (^13^C), and equipped with a gradient-enhanced, reverse-detection probe. Chemical shifts (δ) are indirectly referred to TMS using residual solvent signals. All NMR experiments, including 2D spectra, i.e., g-COSY, g-HSQCAD, and g-HMBCAD, were performed using software supplied by the manufacturer, and acquired at constant temperature (300 K). g-HMBCAD experiments were optimized for a long-range ^13^C-^1^H coupling constant of 8.0 Hz. High-resolution mass spectra were acquired with an Orbitrap Fusion Tribrid® (Q-OT-qIT) mass spectrometer (Thermo Fisher Scientific, Bremen, Germany) equipped with an ESI ion source operating in positive or negative mode. Samples were directly infused and converted to the gas phase using the following parameters: source voltage, 2.6 kV; sheath gas flow rate, 25 au; and auxiliary gas, 8 au. The ions were introduced into the mass spectrometer through a heated ion transfer tube (300 °C). Survey scan was performed from *m/z* 150 to 1000 at 500k resolution (@ 200 *m/z*) using the following parameters: RF lens, 60%; auto gain control target, 20 000.

### 3.2. Synthesis of Compound 3

Honokiol (214.2 mg, 0.76 mmol) was mixed with acetic anhydride (10 mL) and with K_2_CO_3_ (445.3 mg, 3.2 mmol) at rt for 30 min. The mixture was diluted with cold water (10 mL) and partitioned with EtOAC (3 x 15 mL), the combined organic phase was dried over anhydrous Na_2_SO_4_, filtered and taken to dryness. The recovered organic layer was purified by column chromatography on silica gel (*n*-hexane → *n*-hexane:acetone 60:40) to give acetyl honokiol (3) with 56% yield (150.4 mg). Spectroscopic data were in agreement with those previously reported [41]. 

### 3.3. Synthesis of Compound 4

Honokiol (200 mg, 0.75 mmol) was solubilized in dry acetone (15 mL) and the solution was mixed with K_2_CO_3_ (630.3 mg, 4.5 mmol) for 10 min. Then, MeI was added (0.19 mL, 3.2 mmol) and the mixture was refluxed for 24 h. The mixture was filtered out and the permethylated compound 4 was recovered after column chromatography on silica gel (cyclohexane → cyclohexane:acetone 80:20) with 84% yield (185.6 mg). Spectroscopic data were in agreement with those previously reported [42]. 

### 3.4. Synthesis of Compounds 5–7, 10a and b 

The hydrogenation of 2–4, eugenol, and 2-allyl phenol (0.5 mmol) was carried out employing absolute EtOH (7 mL) and Pd/CaCO_3_ (10% *w/w*; 17.4 mg) as catalyst. The reaction flask was filled with H_2_ (1 atm) and stirred at rt for 24 h. The catalyst was removed by filtration on Celite 545. In these conditions the expected products have been obtained quantitatively without further purification. The spectroscopic data of 3′,5-dipropyl-(1,1′-biphenyl)-2,4′-diol (5) [42] 2,4′-dimethoxy-3′,5-dipropyl-1,1′-biphenyl (7) [42] 2-methoxy-4-propylphenol (10a) [45] and 2-propylphenol (10b) [46] were in agreement with literature data.

*3′,5-Dipropyl-(1,1′-biphenyl)-2,4′-diyl diacetate (6).* Yield: 96% (169.7 mg). R*_f_* 0.47 (*n*-hexane:acetone 70:30). ^1^H-NMR (CDCl_3_): δ 7.29 (d, *J* = 2.0 Hz, 1 H, H-2B), 7.25 (dd, *J* = 8.5, 2.0 Hz, 1 H, H-6B), 7.20 (d, *J* = 1.9 Hz, 1 H, H-6A), 7.17 (dd, *J* = 8.2, 1.9 Hz, 1 H, H-4A), 7.06 (d, *J* = 8.5 Hz, 1 H, H-5B), 7.03 (d, *J* = 8.2 Hz, 1 H, H-3A), 2.62 (bdd, *J* = 7.5 Hz, 2 H, C*H*_2_-7A), 2.54 (bdd, *J* = 7.6 Hz, 2 H, C*H*_2_-7B), 2.34 (s, 3 H, OCOC*H*_3_), 2.09 (s, 3 H, OCOC*H*_3_), 1.66 (m, 4 H, C*H*_2_-8A and C*H*_2_-8B), 0.96 (m, 6 H, C*H*_3_-9A and C*H*_3_-9B). ^13^C-NMR (CDCl_3_): δ 169.8 (C, O*C*OCH_3_), 169.6 (C, O*C*OCH_3_), 148.5 (C, C-4B), 145.8 (C, C-2A), 140.9 (C, C-5A), 135.7 (C, C-3B), 134.1 (C, C-1B), 133.9 (C, C-1A), 131.0 (CH, C-4A), 130.9 (CH, C-2B), 128.6 (CH, C-6A), 127.5 (CH, C-6B), 122.6 (CH, C-3A), 122.2 (CH, C-5B), 37.6 (CH_2_, C-7A), 32.4 (CH_2_, C-7B), 24.6 (CH_2_, C-8A), 23.3 (CH_2_, C-8B), 21.1 (CH_3_, OCO*C*H_3_), 21.0 (CH_3_, OCO*C*H_3_), 14.2 (CH_3_, C-9B), 14.0 (CH_3_, C-9A). HRMS (ESI+) *m/z* 377.1739 [M+Na] ^+^ (calcd for C_22_H_26_O_4_Na, 377.1728).

### 3.5. Synthesis of Bromophenols 11a-c

Preliminary experiments for bromination have been performed and the details of these experiments have been reported in Supplementary Materials. 

According to the experiment 7 reported in Supplementary Materials (entry 7 of Table 1), a solution of each compound (10a-b; 5 mmol) in CHCl_3_ (17 mL) was kept in ice bath and a solution containing Br_2_ (300 μL; 1.2 mmol in 10 mL CHCl_3_), was added dropwise. The reaction was monitored by TLC and the Br_2_ was quenched by the addition of a saturated Na_2_S_2_O_3_ solution (15 mL). The mixture was partitioned with CH_2_Cl_2_ (3 x 15 mL) and the organic layer was dried and taken to dryness. 

*5-Bromo-2-methoxy-4-propylphenol (11a).* The expected compound was recovered by column chromatography on silica gel (cyclohexane:EtOAc 98:2 → cyclohexane:EtOAc 96:4) with 63% yield (765.2 mg). R*_f_* 0.48 (cyclohexane:EtOAc 75:25). 

*4-Bromo-2-propylphenol (11b).* The organic layer was purified on silica gel column chromatography (cyclohexane → cyclohexane:EtOAc 95:5) obtaining 11b with 67% yield (720.1 mg). R*_f_* 0.31 (*n*-hexane:acetone 70:30).

*2-Bromo-4-(2-hydroxyethyl)phenol (11c).* Compound 11c was obtained as previously described [44]. Briefly, a solution of tyrosol (10c; 340.3 mg; 2.5 mmol) in acetone (9.2 mL) was stirred with NaBr (514.3 mg; 5 mmol) at −10 °C and a 0.33 M oxone solution (2 gr in 10 mL of H_2_O) was dropwise added. The mixture was stirred for 1 h at −10 °C and then it was partitioned with EtOAc (3 x 10 mL). The combined organic layer was dried over anhydrous Na_2_SO_4_, filtered and taken to dryness. The column chromatography on silica gel (cyclohexane → cyclohexane: acetone 65:35) afforded 11c with 78% yield (423.5 mg).

NMR data of the isolated compounds are in agreement with those previously reported for 11a [47], 11b [48], and 11c [44].

### 3.6. Suzuki–Miyaura Cross-Coupling Reaction: Synthesis of Bisphenols 12a-c

The experimental conditions for the preliminary experiments performed on 11a for S–M reaction have been reported in Supplementary Materials. 

According to entry 4 of these experiments (entry 4 in Table 2), each aryl bromide 11a-c (1.0 mmol) was solubilized in THF (17 mL) and mixed with 4-hydroxyphenylboronic acid (207.2 mg, 1.5 mmol), dppf (165.7 mg, 0.3 mmol), Pd(OAc)_2_ (22.5 mg, 0.1 mmol). Then, a 3 M K_2_CO_3_ solution (1.7 mL, 5.0 mmol) was added and the mixture was stirred at 70 °C for 6 h. The mixture was diluted with water (20 mL) and partitioned with EtOAc (3 x 25 mL). The combined organic layer was washed, dried over anhydrous Na_2_SO_4_, filtered and taken to dryness. The expected bisphenol was recovered after column chromatography. 

*4-Methoxy-6-propyl-(1,1′-biphenyl)-3,4′-diol (12a).* The silica gel column chromatography (petroleum ether → petroleum ether:acetone 92:8) furnished the bisphenol neolignan 12a with 65% yield (167.7 mg). R*_f_* 0.39 (cyclohexane:acetone 70:30). ^1^H-NMR (CDCl_3_): δ 7.14 (d, *J* = 8.5 Hz, 2 H, H-2B/H-6B), 6.85 (d, *J* = 8.5 Hz, 2 H, H-3B/H-5B), 6.78 (s, 1 H, H-2A), 6.76 (s, 1 H, H-5A), 5.52 (bs, 1 H, 3A-O*H*), 5.07 (bs, 1 H, 4B-O*H*), 3.92 (s, 3 H, OC*H*_3_), 2.47 (m, 2 H, C*H*_2_-7A), 1.47 (m, 2 H, C*H*_2_-8A), 0.82 (t, *J* = 7.4 Hz, 3 H, C*H*_3_-9A). ^13^C-NMR (CDCl_3_): δ 154.4 (C, C-4B), 145.7 (C, *C*OCH_3_), 143.1 (C, C-3A), 134.6 (C, C-1A), 134.4 (C, C-1B), 132.2 (CH, C-6A), 130.7 (CH, C-2B/C-6B), 116.4 (C, C-2A), 115.0 (CH, C-3B/C-5B), 111.7 (CH, C-5A), 56.1 (CH_3_, O*C*H_3_), 35.0 (CH_2_, C-7A), 25.0 (CH_2_, C-8A), 14.1 (CH_3_, C-9A). HRMS (ESI-) *m/z* 257.1169 [M-H]^−^ (calcd for C_16_H_17_O_3_, 257.1178). 

*3-Propyl-(1,1′-biphenyl)-4,4′-diol (12b).* The silica gel column chromatography (*n*-hexane:acetone 95:5 → 88:12) afforded the bisphenol neolignan 12b with 70% yield (158.2 mg). R*_f_* 0.35 (cyclohexane:acetone 70:30). ^1^H-NMR ((CD_3_)_2_CO): δ 7.40 (d, *J* = 8.4 Hz, 2 H, H-2B/H-6B), 7.31 (d*, J* = 1.9 Hz, 1 H, H-2A), 7.22 (dd, *J* = 8.2, 1.9 Hz, 1 H, H-6A), 6.88 (d, *J* = 8.4 Hz, 2 H, H-3B/H-5B), 6.87 (d, *J* = 8.2 Hz, 1 H, H-5A), 2.64 (m, 2 H, C*H*_2_-7A), 1.67 (m, 2 H, C*H*_2_-8A), 0.98 (t, *J* = 7.4 Hz, 3 H, C*H*_3_-9A). ^13^C-NMR ((CD_3_)_2_CO): δ 157.1 (C, C-4B), 145.9 (C, C-4A), 133.6 (C, C-1B), 133.2 (C, C-1A), 129.7 (C, C-3A), 129.0 (CH, C-6A), 128.2 (CH, C-3B/C-5B), 125.5 (C, C-2A), 116.3 (CH, C-2B/C-6B), 116.0 (CH, C-5A), 33.1 (CH_2_, C-7A), 23.8 (CH_2_, C-8A), 14.3 (CH_3_, C-9A). HRMS (ESI-) *m/z* 227.1065 [M-H]^−^ (calcd for C_15_H_15_O_2_, 227.1072). 

*5-(2-Hydroxyethyl)-(1,1′-biphenyl)-2,4′-diol (12c).* The neolignan 12c was recovered after column chromatography (cyclohexane.acetone 98:2 → 70:30) with 68% yield (155.7 mg) R*_f_* 0.2 (cyclohexane:acetone 60:40). ^1^H-NMR ((CD_3_)_2_CO): δ 8.30 (bs C-4B-O*H*), 7.91 (bs, C-2A-O*H*), 7.43 (d, *J* = 8.6 Hz, 2 H, H-2B/H-6B), 7.12 (d, *J* = 2.2 Hz, 1 H, H-6A), 6.99 (dd, *J* = 8.2, 2.2 Hz, 1 H, H-4A), 6.87 (d, *J* = 8.6 Hz, 2 H, H-3B/H-5B), 6.85 (d, *J* = 8.2 Hz, 1 H, H-3A), 3.75 (t, *J* = 7.1 Hz, 2 H, C*H*_2_-8A), 3.53 (bs, 1 H, O*H*-8A), 2.76 (t, *J* = 7.1 Hz, 2 H, C*H*_2_-7A). ^13^C-NMR ((CD_3_)_2_CO): δ 157.2 (C, C-4B), 153.1 (C, C-2A), 131.7 (CH, C-6A), 131.6 (C, C-5A), 131.2 (CH, C-2B/C-6B), 131.0 (C, C-1B), 129.1 (CH, C-4A), 129.0 (C, C-1A), 116.7 (CH, C-3A), 115.7 (CH, C-3B/C-5B), 64.2 (CH_2_, C-8A), 39.5 (CH_2_, C-7A). HRMS (ESI-) S *m/z* 229.0874 [M-H]^−^ (calcd for C_14_H_13_O_3_, 229.0865).

### 3.7. Synthesis of O-Allyloxy Neolignans 8 and 13a-c

A solution of the proper bisphenol neolignan (2, 12a-c; 0.5 mmol) in dry acetone (5 mL) was mixed with K_2_CO_3_ (275.7 mg; 2.0 mmol) for 10 min, then, allyl bromide (130 μL; 1.5 mmol) was added and the mixture was refluxed overnight. The mixture was filtered and where necessary it was chromatographed. 

*3′,5-Diallyl-2,4′-bis(allyloxy)-1,1′-biphenyl (8).* The expected compound was recovered with 97% yield (184.9 mg) after filtration of the mixture. R*_f_* 0.79 (cyclohexane:acetone 70:30). Spectroscopic data were in agreement with those reported in the literature [20]. 

*4′,5-Bis(allyloxy)-4-methoxy-2-propyl-1,1′-biphenyl (13a)*. The mixture was purified on Sephadex-LH20 column chromatography eluting with CH_2_Cl_2_, to give 13a with 95% yield (160.5 mg). R*_f_* 0.69 (cyclohexane:acetone 70:30). ^1^H-NMR (CDCl_3_): δ 7.19 (d, *J* = 8.6 Hz, 2 H, H-2B/H-6B), 6.94 (d, *J* = 8.6 Hz, 2 H, H-3B/H-5B), 6.78 (s, 1 H, H-5A), 6.73 (s, 1 H, H-2A), 6.11 (m, 1 H, H-11A), 6.07 (m, 1 H, H-8B), 5.45 (dd, *J* = 15.0, 5.0 Hz, 1 H, C*H*_a_-12A), 5.39 (dd, *J* = 15.0, 5.0 Hz, 1 H, C*H*_a_-9B), 5.31 (dd, *J* = 10.0, 5.0 Hz, 1 H, C*H*_b_-12A), 5.26 (dd, *J* = 10.0, 5.0 Hz, 1 H, C*H*_b_-9B), 4.59 (d, *J* = 5.3 Hz, 2 H, C*H*_2_-7B), 4.58 (d, *J* = 5.4 Hz, 2 H, C*H*_2_-10A), 3.91 (s, 3 H, OC*H*_3_), 2.48 (m, 2 H, C*H*_2_-7A), 1.49 (m, 2 H, C*H*_2_-8A), 0.32 (t, *J* = 7.3 Hz, 3 H, C*H*_3_-9A). ^13^C-NMR (CDCl_3_): δ 157.7 (C, C-4B), 148.5 (C, *C*OCH_3_), 145.7 (C, C-3A), 134.6 (C, C-1B), 133.8 (C, C-1A), 133.7 (CH, C-11A), 133.5 (CH, C-8B), 133.1 (C, C-6A), 130.5 (CH, C-2B/C-6B), 117.9 (CH_2_, C-9B), 117.8 (CH_2_, C-12A), 115.8 (CH, C-2A), 114.4 (CH, C-3B/C-5B), 112.9 (CH, C-5A), 70.1 (CH_2_, C-10A), 69.0 (CH_2_, C-7B), 56.2 (CH_3_, O*C*H_3_), 35.0 (CH_2_, C-7A), 24.9 (CH_2_, C-8A), 14.2 (CH_3_, C-9A). HRMS (ESI+) *m/z* 361.1791 [M+Na]^+^ (calcd for C_22_H_26_O_3_Na, 361.1780). 

*4,4′-Bis(allyloxy)-3-propyl-1,1′-biphenyl (13b).* The expected compound was recovered after filtration without further purification with 96% yield (147.2 mg). R*_f_* 0.79 (cyclohexane:acetone 75:25). ^1^H-NMR (CDCl_3_): δ 7.47 (d, *J* = 8.7 Hz, 2 H, H-2B/H-6B), 7.33 (d, *J* = 1.9 Hz, 1 H, H-2A), 7.31 (dd, *J* = 8.2, 1.9 Hz, 1 H, H-6A), 6.97 (d, *J* = 8.7 Hz, 2 H, H-3B/H-5B), 6.87 (d, *J* = 8.2 Hz, 1 H, H-5A), 6.09 (m, 2 H, H-11A and H-8B), 5.45 (m, 2H, C*H_2_*-12A), 5.30 (m, *2* H, C*H*_2_-9B), 4.58 (d, *J* = 5.1 Hz, 4 H, C*H*_2_-10A and C*H*_2_-7B), 2.68 (t, *J* = 7.5 Hz, 2 H, C*H*_2_-7A), 1.68 (sext, *J* = 7.5 Hz, 2 H, C*H*_2_-8A), 0.98 (t, *J* = 7.5, 3 H, C*H*_3_-9A). ^13^C-NMR (CDCl_3_): δ 157.8 (C, C-4B), 155.8 (C, C-4A), 134.1 (C, C-1B), 133.3 (C, C-1A), 133.8 (CH, C-11A), 133.5 (CH, C-8B), 131.8 (C, C-3A), 128.6 (CH, C-2A), 127.9 (CH, C-2B/C-6B), 125.0 (CH, C-6A), 117.8 (CH_2_, C-9B), 116.9 (CH_2_, C-12A), 115.1 (CH, C-3B/C-5B), 112.0 (CH, C-5A), 69.1 (CH_2_, C-10A), 68.1 (CH_2_, C-7B), 32.7 (CH_2_, C-7A), 23.3 (CH_2_, C-8A), 14.3 (CH_3_, C-9A). HRMS (ESI+) *m/z* 331.1683 [M+Na]^+^ (calcd for per C_21_H_24_O_2_Na, 331.1674).

*5-(2-Hydroxyethyl)-2,4′-bis(allyloxy)-1,1′-biphenyl (13c).* The Sephadex-LH20 column chromatography (CH_2_Cl_2_) afforded the expected *O*-allyl derivative 13c with 75% yield (116.8 mg). R*_f_* 0.49 (cyclohexane.acetone 65:35). ^1^H-NMR ((CD_3_)_2_CO): δ 7.49 (d, *J* = 8.8 Hz, 2 H, H-2B/H-6B), 7.18 (d, *J* = 1.9 Hz, 1 H, H-6A), 7.13 (dd, *J* = 8.3, 1.9 Hz, 1 H, H-4A), 6.97 (d, *J* = 8.7 Hz, 2 H, H-3B/H-5B), 6.96 (d, *J* = 8.3 Hz, 1 H, H-3A), 6.10 (m, 1 H, H-11A), 6.01 (m, 1 H, H-8B), 5.44 (dd, *J* = 17.3, 1.5 Hz, 1 H, C*H*_a_-12A), 5.34 (dd, *J* = 17.3, 1.7 Hz, 1 H, C*H*_a_-9B), 5.25 (dd, *J* = 10.6, 1.5 Hz, 1 H, C*H*_b_-12A), 5.16 (dd, *J* = 10.6, 1.7 Hz, 1 H, C*H*_b_-9B), 4.61(d, *J* = 5.2 Hz, 2 H, CH_2_-10A), 4.53 (d, *J* = 3.2 Hz, 2 H, CH_2_-7B), 3.75 (t, *J* = 6.6 Hz, 2 H, C*H*_2_-8A), 2.79 (t, *J* = 6.6 Hz, 2 H, C*H*_2_-7A). ^13^C-NMR ((CD_3_)_2_CO): δ 158.6 (C, C-4B), 154.8 (C, C-2A), 134.88 (CH, C-11A), 134.86 (CH, C-8B), 133.2 (C, C-5A), 132.2 (C, C-1B), 131.9 (CH, C-6A), 131.4 (CH, C-2B/C-6B), 131.1 (C, C-1A), 129.4 (CH, C-4A), 117.3 (CH_2_, C-9B), 116.7 (CH, C-3A), 114.9 (CH, C-3B/C-5B), 113.9 (CH_2_, C-12A), 69.8 (CH_2_, C-10A), 69.3 (CH_2_, C-7B), 64.1 (CH_2_, C-8A), 39.5 (CH_2_, C-7A). HRMS (ESI-) *m/z* 309.1501 [M-H]^−^ (calcd for C_20_H_21_O_3_, 309.1491).

### 3.8. General Procedure for Claisen Rearrangement: Synthesis of Bisphenols 14a-c and 15a 

A 1 M Et_2_AlCl solution (in *n-*hexane; 1.5 mL) was added dropwise to a solution of *O*-allyl derivatives 8, 13a-c (0.35 mmol) in dry CH_2_Cl_2_ (2 mL). The mixture was stirred at rt for 2 h and then the reaction was quenched adding 2 N HCl solution (5 mL). The mixture was partitioned with CH_2_Cl_2_ (2 × 5 mL); the combined organic phase was washed with water, dried oved anhydrous Na_2_SO_4_, filtered and taken to dryness. Column chromatography on Sephadex-LH20 (CH_2_Cl_2_) afforded the pure products.

*3,3′,5,5′-Tetraallyl-(1,1′-biphenyl)-2,4′-diol (9).* Yield: 20% (24.7 mg). R*_f_* 0.55 (cyclohexane.acetone 70:30). Spectroscopic data were in agreement with those previously reported [43]. 

*2,3′-Diallyl-4-methoxy-6-propyl-(1,1′-biphenyl)-3,4′-diol (14a).* Yield: 18% (21.5 mg). R*_f_* 0.44 (cyclohexane:acetone 70:30). ^1^H-NMR (CDCl_3_): δ 6.88 (m, 1 H, H-6B), 6.87 (bs, 1 H, H-2B), 6.81 (d, *J* = 7.9 Hz, 1 H, H-5B), 6.66 (s, 1 H, H-5A), 6.03 (m, 1 H, H-8B), 5.82 (m, 1 H, H-11A), 5.59 (bs, 1H, 3A-O*H*), 5.13 (m, 2 H, CH_2_-9B), 4.96 (bs, 1 H, 4B-O*H*), 4.87 (bd, *J* = 10.1 Hz, 1 H, C*H*_a_-12A), 4.75 (bd, *J* = 17.1 Hz, 1 H, C*H*_b_-12A), 3.91 (s, 3 H, OC*H*_3_), 3.41 (d, *J* = 6.2 Hz, 2 H, C*H*_2_-7B), 3.12 (d, *J* = 6.1 Hz, 2 H, C*H*_2_-10A), 2.24 (bdd, *J* = 7.5 Hz, 2 H, C*H*_2_-7A), 1.40 (sext, *J* = 7.5 Hz, 2 H, C*H*_2_-8A), 0.77 (t, *J* = 7.5 Hz, 3 H, C*H*_3_-9A). ^13^C-NMR (CDCl_3_): δ 152.7 (C, C-4B), 145.2 (C, *C*OCH_3_), 141.4 (C, C-3A), 136.8 (CH, C-11A), 136.4 (CH, C-8A), 134.5 (C, C-1A), 132.6 (C, C-6A), 132.5 (C, C-1B), 132.3 (CH, C-2B), 129,4 (CH, C-6B), 124.5 (C, C-2A), 124.4 (C,C-3B), 116.3 (CH, C-5B), 115.2 (CH_2_, C-9B), 114.4 (CH_2_, C-12A), 109.1 (CH, C-5A), 55.9 (CH_3_, O*C*H_3_), 35.8 (CH_2_, C-7A), 34.9 (CH_2_, C-7B), 32.1 (CH_2_, C-10A), 24.9 (CH_2_, C-8A), 14.0 (CH_3_, C-9A). HRMS (ESI-) *m/z* 337.2123 [M-H]^−^ (calcd for C_22_H_25_O_3_, 337.2117). 

*3,3′-Diallyl-5-propyl-(1,1′-biphenyl)-4,4′-diol (14b).* Yield: 40% (42.8 mg) R*_f_* 0.48 (cyclohexane:acetone 70:30). ^1^H-NMR (CDCl_3_): δ 7.31 (dd, *J* = 8.2, 2.1 Hz, 1 H, H-6B), 7.28 (d, *J* = 2.1 Hz, 1 H, H-2B), 7.19 (d, *J* = 1.5 Hz, 1 H, H-2A), 7.13 (d, *J* = 1.5 Hz, 1 H, H-6A), 6.85 (d, *J* = 8.2 Hz, 1 H, H-5B), 6.06 (m, 2 H, H-11A and H-8B), 5.21 (m, 4 H, C*H*_2_-12A and C*H*_2_-9B), 4.98 (bs, 1H, 4A-O*H*) 4.96 (bs, 1 H, 4B-O*H*), 3.47 (d, *J* = 6.3 Hz, 4 H, C*H*_2_-10A and C*H*_2_-7B), 2.73 (bt, *J* = 7.7 Hz, 2 H, C*H*_2_-7A), 1.68 (sext, *J* = 7.5 Hz, 2 H, C*H*_2_-8A), 1.0 (t, *J* = 7.5 Hz, 3 H, C*H*_3_-9A). ^13^C-NMR (CDCl_3_): δ 153.3 (C, C-4B), 151.7 (C, C-4A), 136.6 (CH, C-11A and C-6A), 136.5 (CH, C-8B), 134.3 (C, C-1B), 133.4 (C, C-1A), 129,4 (C, C-3A), 127.2 (CH, C-2A), 126.4 (CH, C-6B), 125.5 (C, C-3B), 125.0 (C, C-5A), 117.0 (CH_2_, C-12A), 116.8 (CH_2_, C-9B), 116.3 (CH, C-5B), 36.1 (CH_2_, C-10A), 35.6 (CH_2_, C-7B), 32.5 (CH_2_, C-7A), 23.3 (CH_2_, CH_2_-8A), 14.3 (CH_3_, C-9A). HRMS (ESI-) *m/z* 307.1707 [M-H]^−^ (calcd for C_21_H_23_O_2_, 307.1698). 

*3,3′-Diallyl-5-(2-hydroxyethyl)-(1,1′-biphenyl)-2,4′-diol (14c).* Yield: 48% (51.1 mg). R*_f_* 0.42 (cyclohexane:acetone 65:35). ^1^H-NMR ((CD_3_)_2_CO): δ 7.15 (bs, 1 H, H-6A), 7.12 (bd, *J* = 8.2 Hz, 1 H, H-6B), 6.91, (bs, 1 H, H-4A), 6.90 (bs, 1 H, H-2B), 6.88 (d, *J* = 8.2 Hz, 1 H, H-5B), 6.00 (m, 2 H, H-11A and H-8B), 5.09 (m, 2 H, C*H*_2_-12A), 5.04 (m, 2 H, C*H*_2_-9B), 3.80 (t, *J* = 6.6 Hz, 2 H, C*H*_2_-8A), 3.40 (bt, *J* = 7.1 Hz, 4 H, C*H*_2_-10A and C*H_2_*-7B), 2.77 (t, *J* = 6.6 Hz, 2 H, C*H*_2_-7A). ^13^C-NMR ((CD_3_)_2_CO): δ 154.0 (C, C-4B), 148.8 (C, C-2A), 136.5 (CH, C-11A), 136.1 (CH, C-8B), 130.7 (CH, C-6A), 129.7 (C, C-5A), 129.4 (CH, C-4A), 128.7 (C, C-3A), 128.5 (CH, C-2B), 128.0 (CH, C-6B), 127.9 (C, C-3B), 126.8 (C, C-1A), 126.1 (C, C-1B), 115.88 (CH_2_, C-12A), 115.86 (CH_2_, C-9B), 115.5 (CH, C-5B), 63.5 (CH_2_, C-8A), 38.1 (CH_2_, C-7A), 34.5 (CH_2_, C-10A), 34.3 (CH_2_, C-7B). HRMS (ESI-) *m/z* 309.1484 [M-H]^−^ (calcd for C_20_H_21_O_3_, 309.1491).

*2-Allyl-4′-(allyloxy)-4-methoxy-6-propyl-(1,1′-biphenyl)-3-ol (15a).* Yield: 15% (17.7 mg). R*_f_* 0.52 (cyclohexane:acetone 70:30). ^1^H-NMR (CDCl_3_): δ 7.03 (d, *J* = 7.5 Hz, 2 H, H-2B/H-6B), 6.92 (d, *J* = 7.5 Hz, 2 H, H-3B/H-5B), 6.66 (s, 1 H, H-5A), 6.11 (m, 1 H, H-8B), 5.82 (m, 1 H, H-11A), 5.59 (bs, 1 H, O*H*), 5.46 (dd, *J* = 17.2, 1.2 Hz, 1 H, C*H*_a_-9B), 5.31 (dd, *J* = 10.5, 1.2 Hz, 1 H, C*H*_b_-9B), 4.87 (dd, *J* = 10.1, 1.4 Hz, 1 H, C*H*_a_-12A), 4.76 (dd, *J* = 17.0, 1.4 Hz, 1 H, C*H*_b_-12A), 4.58 (d, *J* = 7.5 Hz, 2 H, C*H*_2_-7B), 3.91 (s, 3 H, OC*H*_3_), 3.12 (d, *J* = 6.1 Hz, 2 H, C*H*_2_-10A), 2.24 (m, 2 H, C*H*_2_-7A), 1.40 (m, 2 H, C*H*_2_-8A), 0.76 (t, *J* = 7.5 Hz, 3 H, C*H*_3_-9A). ^13^C-NMR (CDCl_3_): δ 157.5 (C, C-4B), 145.4 (C, *C*OCH_3_), 141.5 (C, C-3A), 136.9 (CH, C-11A), 134.6 (C, C-1A), 133.6 (CH, C-8B), 132.7 (C, C-6A), 132.6 (C, C-1B), 131.3 (CH, C-2B/C-6B), 124.6 (C, C-2A), 117.8 (CH_2_, C-9B), 114.6 (CH_2_, C-12A), 114.1 (CH, C-3B/C-5B), 109.3 (CH, C-5A), 69.0 (CH_2_, C-7B), 56.1 (CH_3_, O*C*H_3_), 35.9 (CH_2_, C-7A), 32.1 (CH_2_, C-10A), 24.9 (CH_2_, C-8A), 14.2 (CH_3_, C-9A). HRMS (ESI-) *m/z* 337.1812 [M-H]^−^ (calcd for C_22_H_25_O_3_, 337.1804).

### 3.9. Human Cell Cultures 

Two human colorectal adenocarcinoma cell lines (HCT-116 and HT-29) and one human prostate adenocarcinoma cell line (PC3) have been used in the present work and been cultured as previously reported [28,49]. The HCT-116 (ATCC number: CCL-247) cell line was cultured in McCoy’s 5A (Medium, GlutaMAX™ Supplement, cod. 36600021 containing 3 g/L of d-glucose), HT-29 (ATCC number: HTB-38) was cultured in in DMEM medium (Dulbecco’s Modified Eagle Medium 1X; GIBCO, Cat No. 31965-023 containing 4.5g/L of d-glucose) while PC3 cell line (ATCC number: CRL-1435) was cultured in DMEM/F-12, (Dulbecco’s Modified Eagle Medium/Nutrient Mixture F-12, GlutaMAX™ Supplement, cod. 31331028). Each medium was supplemented with 10% Fetal Bovine Serum (FBS) and 100 U/ml of penicillin-streptomycin. The cell cultures were incubated at 37 °C in humidified atmosphere with 5% of CO_2_ and 95% of air. The medium was changed twice in the week.

### 3.10. Antiproliferative Assay 

HCT-116 HT-29 and PC3 cell lines (1.8 and 2.5 × 10^3^ cells/0.33 cm^2^ respectively) were plated in 96 well plates from “Nunclon™ Microwell™” (Thermo Fisher Scientific, Bremen, Germany), and were incubated at 37 °C. After 24 h cells (60% confluence) were treated with compounds 2-9, 12a-c, 13a-c, 14a-c, and 15a at increasing concentration from 0.001 to 100 µM. The cellular vitality and/or the cellular cytotoxicity has been evaluated by colorimetric assay with tetrazolium salt MTT (3-(4,5-methylthiazol-2-yl)-2,5-diphenyltetrazolium bromide) as previously reported [27]. After 72 hours of treatment with the bisphenol neolignans, MTT salt was added and maintained for 3 hours. The purple formazan dye, produced by the metabolic process of vital cells, was solubilized by adding 150 µL/well of DMSO (dimethyl sulfoxide) and the optical density (OD) values were read on a multiwell scanning spectrophotometer (Multiskan™ reader, Thermo Fisher Scientific, Bremen, Germany), using a wavelength of 570 nm. Each value was the average of four–six wells. The percentage of cellular growth was calculated according to NCI [50]: 100 × (T − T_0_) / (C−T_0_) (where T is the optical density of the test well after a 72 h period of exposure to test compound, T_0_ is the optical density at time zero, and C is the optical density of the untreated control cell cultures). When the optical density of treated cells was lower than the T_0_ value the following formula was used: 100 × (T − T_0_) /T_0_. GI_50_ values were calculated by the software GraphPad Prism v.6. 

### 3.11. Apoptosis Analysis by Imaging Flow Cytometry

HCT116 and PC3 cell lines were cultured in six-well plates (about 3.5 × 10^5^ cells/9.6 cm^2^) and treated with the following compounds: 14a and c, and 15a. Vehicle-treated cells and the anticancer 5-fluorouracil (5-FU, final concentration of 10 µM) were used as negative and positive control, respectively. Cells were harvested by trypsinization after 72 h of treatment, washed in PBS, and stained with Alexa Fluor® 488 dye conjugated to annexin V and propidium iodide (PI) in according to manufacturing protocol (The Alexa Fluor® 488 annexin V /Dead Cell Apoptosis Kit by Invitrogen, Cat. V13245; Thermo Fisher Scientific, Bremen, Germany). PI is impermeable to live cells and to cells in early apoptosis, but stains dead cells and late apoptosis cells with red fluorescence, binding tightly to the nucleic acids while Alexa Fluor® 488 dye, conjugated to annexin V, stains in green the apoptotic cells. After staining the cellular suspension was analyzed on a flow cytometer (Amnis FlowSight Millipore, Merck KgaA, Darmstadt, Germany), and results were analyzed using Image Data Exploration and Analysis (IDEAS) software (Amnis part of EMD Millipore, Seattle, WA, USA).

## 4. Conclusions

The present work reports the synthesis of a library of bisphenol neolignans inspired by honokiol. These products were evaluated for their potential antiproliferative activity against three cancer cell lines, namely HCT-116, HT-29 (both human colorectal adenocarcinoma) and PC3 (human prostate adenocarcinoma). This study suggests some structural motifs important for the activity and overall highlights that bisphenol neolignans 9, 12b, 14a-c, and 15a, structurally related to honokiol, show higher antiproliferative activity than the natural lead. The most promising antiproliferative agents (14a, 14c, and 15a) were selected for a flow cytometric analysis; this showed that the inhibition of proliferation is mainly due to apoptotic process. In conclusion, some of the bisphenol neolignans reported in the present study have shown promising properties for the development of new natural-derived antitumor agents.

## Figures and Tables

**Table 1:** Reactions for bromination of 10a.

Entry	Brominating Agent	Catalyst	Solvent^1^	% Yield (11a)^2^
1	NBS	I_2_	CH_3_CN	10
2	NBS	I_2_	CHCl_3_	12
3	NBS	AlCl_3_	CH_3_CN	15
4	NBS	AlCl_3_	CHCl_3_	25
5	Br_2_	AlCl_3_	CH_3_CN	18
6	Br_2_	/	CHCl_3_	47
7^3^	Br_2_	/	CHCl_3_	63
8^4^	NaBr/oxone	/	acetone/water	5

^1^ If it is not indicated, the reactions were carried out at rt. ^2^ The yield was determined by HPLC-UV. ^3^ The reaction was carried out at 0 °C. ^4^ The reaction was performed at −10 °C.

**Table 2:** Reactions for Suzuki–Miyaura cross coupling of 11a.

Entry	Solvent	Temperature	% Yield (12a)^1^
1	THF	25 °C	5
2	THF	70 °C	10
3	THF/H_2_O^2^	70 °C	20
4	THF/H_2_O^3^	70 °C	67
5	1,4-dioxane	70 °C	6
6	1,4-dioxane	180 °C	8

^1^ The yield was determined by HPLC-UV. ^2^ The concentration of bromide 11a was 0.10 M. ^3^ The concentration of bromide 11a was 0.05 M.

**Table 3:** Antiproliferative activity of bisphenol neolignans inspired by honokiol.

Compound	GI_50_ (µM) ± SD ^1^		
	HCT-116	HT-29	PC3
2	18.2 ± 2.1	40.6 ± 3.9	52.1 ± 7.1
3	22.5 ± 3.4	> 100	51.3 ± 7.2
4	63.9 ± 5.9	> 100	> 100
5	21.2 ± 2.6	9.9 ± 2.1	10.5 ± 1.7
6	20.1 ± 2.9	> 100	20.6 ± 3.2
7	64.7 ± 7.9	70.2 ± 8.7	> 100
8	38.1 ± 0.6	40.7 ± 0.5	> 100
9	11.1 ± 0.9	9.8 ± 1.4	19.8 ± 1.8
12a	24.1 ± 3.0	65.9 ± 8.3	> 100
12b	14.7 ± 2.1	20.5 ± 3.1	47.9 ± 4.7
12c	> 100	> 100	> 100
13a	> 100	> 100	> 100
13b	69.7 ± 6.9	42.3 ± 4.7	> 100
13c	84.5 ± 8.0	42.0 ± 4.1	> 100
14a	5.3 ± 1.5	13.0 ± 2.0	5.8 ± 1.9
14b	8.2 ± 1.1	12.3 ± 1.6	17.2 ± 1.5
14c	3.7 ± 0.7	11.3 ± 2.2	19.1 ± 2.6
15a	3.6 ± 0.6	12.7 ± 2.1	8.9 ± 2.0
5-FU	6.2 ± 0.8	7.3 ± 0.7	9.0 ± 0.9

^1^ GI_50_ value were calculated after 72 h of continuous exposure relative to untreated controls; values are the mean (±SD) of four experiments. HCT-116 and HT-29: human colorectal adenocarcinoma cells. PC3: human prostate cancer cells. 5-FU: 5-fluorouracil.

**Table 4:** Apoptotic death of 14a, 14c, and 15a in HCT-116 and PC3 Cells^1^.

Entry	HTC-116 Cell Distribution (%)					PC3 Cell Distribution (%)				
	Ctrl	14a	14c	15a	5-FU	Ctrl	14a	14c	15a	5-FU
Live	82.1	74.8	70.9	46.3	17.0	92.7	79.9	86.5	61.3	24.0
Early apoptosis (Annexin+)	1.5	9.5	6.0	17.4	55.0	0.9	12.4	5.3	26.0	50.0
Necrotic cells (PI+)	1.6	4.8	4.4	0.4	0.2	1.7	0.1	0.2	0.4	0.3
Late apoptosis (Annexin+/PI+)	14.9	10.9	18.7	35.9	27.8	4.7	7.7	8.0	12.4	25.7

^1^ Determined by Alexa Fluor 488; annexin V/propidium iodide (annexin/PI) staining after treatment with 14a, 14c, 15a, and 5-FU (10 μM) for 72 h. The analysis was performed on 10,000 events for each condition and expressed in percentage of total number of events.

## Supplementary Materials

A copy of HRMS and NMR spectra of the new synthesized bisphenol neolignans is available online at https://www.mdpi.com/1420-3049/25/3/733/s1.

## References

[1] Zhang J., Chen J.J., Liang Z.Z., Zhao C.Q. (2014). New lignans and their biological activities. Chem. Biodivers..

[2] Aldemir H., Richarz R., Gulder T.A.M. (2014). The biocatalytic repertoire of natural biaryl formation. Ange. Chem. Int. Ed..

[3] Lee Y.J., Lee Y.M., Lee C.K., Jung J.K., Han S.B., Hong J.T. (2011). Therapeutic applications of compounds in the Magnolia family. Pharmacol. Ther..

[4] Patočka J., Jakl J., Strunecká A. (2006). Expectations of biologically active compounds of the genus *Magnolia* in biomedicine. J. Appl. Biomed..

[5] Kelm M.A., Nair M.G. (2000). A brief summary of biologically active compounds from *Magnolia* spp. Stud. Nat. Prod. Chem..

[6] Shen J.L., Man K.M., Huang P.H., Chen W.C., Chen D.C., Cheng Y.W., Liu P.L., Chou M.C., Chen Y.H. (2010). Honokiol and magnolol as multifunctional antioxidative molecules for dermatologic disorders. Molecules.

[7] Amorati R., Zotova J., Baschieri A., Valgimigli L. (2015). Antioxidant activity of magnolol and honokiol: Kinetic and mechanistic investigations of their reaction with peroxyl radicals. J. Org. Chem..

[8] Lin Y.R., Chen H.H., Ko C.H., Chan M.H. (2007). Effects of honokiol and magnolol on acute and inflammatory pain models in mice. Life Sci..

[9] Lin Y.R., Chen H.H., Ko C.H., Chan M.H. (2006). Neuroprotective activity of honokiol and magnolol in cerebellar granule cell damage. Eur. J. Pharmacol..

[10] Fried L.E., Arbiser J.L. (2009). Honokiol, a multifunctional antiangiogenic and antitumor agent. Antioxid. Redox Signal..

[11] Ranaware A.M., Banik K., Deshpande V., Padmavathi G., Roy N.K., Sethi G., Fan L., Kumar A.P., Kunnumakkara A.B. (2018). Magnolol: A neolignan from the Magnolia family for the prevention and treatment of cancer. Int. J. Mol. Sci..

[12] Kim G.D., Oh J., Park H.J., Bae K., Lee S.K. (2013). Magnolol inhibits angiogenesis by regulating ROS–Mediated apoptosis and the PI3K/AKT/mTOR signaling pathway in mES/EB-derived endothelial-like cells. Int. J. Oncol..

[13] Shen J., Ma H.L., Zhang T.C., Liu H., Yu L.H., Li G.S., Li H.S., Hu M.C. (2017). Magnolol inhibits the growth of non-small cell lung cancer via inhibiting microtubule polymerization. Cell. Physiol. Biochem..

[14] Shen L., Zhang F., Huang R.M., Yan J., Shen B. (2017). Honokiol inhibits bladder cancer cell invasion through repressing SRC-3 expression and epithelial-mesenchymal transition. Oncol. Lett..

[15] Yeh P.S., Wang W., Chang Y.A., Lin C.J., Wang J.J., Chen R.M. (2016). Honokiol induces autophagy of neuroblastoma cells through activating the PI3K/Akt/mTOR and endoplasmic reticular stress/ERK1/2 signaling pathways and suppressing cell migration. Cancer Lett..

[16] Hajduk P.J., Bures M., Praestgaard J., Fesik S.W. (2000). Privileged molecules for protein binding identified from NMR-based screening. J. Med. Chem..

[17] Jada S., Doma M.R., Singh P.P., Kumar S., Malik F., Sharma A., Khan I.A., Qazi G.N., Kumar H.M.S. (2012). Design and synthesis of novel magnolol derivatives as potential antimicrobial and antiproliferative compounds. Eur. J. Med. Chem..

[18] Amblard F., Govindarajan B., Lefkove B., Rapp K.L., Detorio M., Arbiser J.L., Schinazi R.F. (2007). Synthesis, cytotoxicity, and antiviral activities of new neolignans related to honokiol and magnolol. Bioorg. Med. Chem. Lett..

[19] Tripathi S., Chan M.H., Chen C.P. (2012). An expedient synthesis of honokiol and its analogues as potential neuropreventive agents. Bioorg. Med. Chem. Lett..

[20] Lee S.H., Fei X., Lee C., Do H.T.T., Rhee I., Seo S.Y. (2019). Synthesis of either C2-or C4′-alkylated derivatives of honokiol and their biological evaluation for anti-inflammatory activity. Chem. Pharm. Bull..

[21] Lin J.M., Gowda A.S.P., Sharma A.K., Amin S. (2012). In vitro growth inhibition of human cancer cells by novel honokiol analogs. Bioorg. Med. Chem..

[22] Maioli M., Basoli V., Carta P., Fabbri D., Dettori M.A., Cruciani S., Serra P.A., Delogu G. (2018). Synthesis of magnolol and honokiol derivatives and their effect against hepatocarcinoma cells. PLoS ONE.

[23] Sanchez-Peris M., Murga J., Falomir E., Carda M., Marco J.A. (2017). Synthesis of honokiol analogues and evaluation of their modulating action on VEGF protein secretion and telomerase-related gene expressions. Chem. Biol. Drug Des..

[24] Kotha S., Lahiri K., Kashinath D. (2002). Recent applications of the Suzuki–Miyaura cross-coupling reaction in organic synthesis. Tetrahedron.

[25] Martin R., Buchwald S.L. (2008). Palladium-catalyzed Suzuki–Miyaura cross-coupling reactions employing dialkylbiaryl phosphine ligands. Acc. Chem. Res..

[26] Pulvirenti L., Muccilli V., Cardullo N., Spatafora C., Tringali C. (2017). Chemoenzymatic synthesis and alpha-glucosidase inhibitory activity of dimeric neolignans inspired by magnolol. J. Nat. Prod..

[27] Cardullo N., Spatafora C., Musso N., Barresi V., Condorelli D., Tringali C. (2015). Resveratrol-related polymethoxystilbene glycosides: Synthesis, antiproliferative activity, and glycosidase inhibition. J. Nat. Prod..

[28] Cardullo N., Pulvirenti L., Spatafora C., Musso N., Barresi V., Condorelli D.F., Tringalii C. (2016). Dihydrobenzofuran neolignanamides: Laccase-mediated biomimetic synthesis and antiproliferative activity. J. Nat. Prod..

[29] Chillemi R., Cardullo N., Greco V., Malfa G., Tomasello B., Sciuto S. (2015). Synthesis of amphiphilic resveratrol lipoconjugates and evaluation of their anticancer activity towards neuroblastoma SH-SY5Y cell line. Eur. J. Med. Chem..

[30] Capolupo A., Tosco A., Mozzicafreddo M., Tringali C., Cardullo N., Monti M.C., Casapullo A. (2017). Proteasome as a new target for bio-inspired benzo[*k*,*l*]xanthene lignans. Chem. Eur. J..

[31] Spatafora C., Barresi V., Bhusainahalli V.M., Di Micco S., Musso N., Riccio R., Bifulco G., Condorelli D., Tringali C. (2014). Bio-inspired benzo[*k*,*l*]xanthene lignans: Synthesis, DNA-interaction and antiproliferative properties. Org. Biomol. Chem..

[32] Cardullo N., Catinella C., Floresta G., Muccilli V., Rosselli S., Rescifina A., Bruno M., Tringali C. (2019). Synthesis of rosmarinic acid amides as antioxidative and hypoglycemic agents. J. Nat. Prod..

[33] Cardullo N., Muccilli V., Pulvirenti L., Cornu A., Poiségu L., Deffieux D., Quideau S., Tringali C. (2020). *C*-glucosidic ellagitannins and galloylated glucoses as potential functional food ingredients with anti-diabetic properties: A study of α-glucosidase and α-amylase inhibition. Food Chem..

[34] Genovese C., Pulvirenti L., Cardullo N., Muccilli V., Tempera G., Nicolosi D., Tringali C. (2018). Bioinspired benzoxanthene lignans as a new class of antimycotic agents: Synthesis and *Candida* spp. growth inhibition. Nat. Prod. Res..

[35] Di Micco S., Spatafora C., Cardullo N., Riccio R., Fischer K., Pergola C., Koeberle A., Werz O., Chalal M., Vervandier-Fasseur D. (2016). 2,3-Dihydrobenzofuran privileged structures as new bioinspired lead compounds for the design of mPGES-1 inhibitors. Bioorg. Med. Chem..

[36] Gerstmeier J., Kretzer C., Di Micco S., Miek L., Butschek H., Cantone V., Bilancia R., Rizza R., Troisi F., Cardullo N. (2019). Novel benzoxanthene lignans that favorably modulate lipid mediator biosynthesis: A promising pharmacological strategy for anti-inflammatory therapy. Biochem. Pharmacol..

[37] Di Micco S., Pulvirenti L., Bruno I., Terracciano S., Russo A., Vaccaro M.C., Ruggiero D., Muccilli V., Cardullo N., Tringali C. (2018). Identification by inverse virtual screening of magnolol-based scaffold as new tankyrase-2 inhibitors. Bioorg. Med. Chem..

[38] Baschieri A., Pulvirenti L., Muccilli V., Amorati R., Tringali C. (2017). Chain-breaking antioxidant activity of hydroxylated and methoxylated magnolol derivatives: The role of H-bonds. Org. Biomol. Chem..

[39] Cardile V., Lombardo L., Spatafora C., Tringali C. (2005). Chemo-enzymatic synthesis and cell-growth inhibition activity of resveratrol analogues. Bioorg. Chem..

[40] Ng S.Y., Cardullo N., Yeo S.C.M., Spatafora C., Tringali C., Ong P.-S., Lin H.-S. (2014). Quantification of the resveratrol analogs trans-2,3-dimethoxy-stilbene and trans-3,4-dimethoxystilbene in rat plasma: Application to pre-clinical pharmacokinetic studies. Molecules.

[41] Schuhly W., Hufner A., Pferschy-Wenzig E.M., Prettner E., Adams M., Bodensieck A., Kunert O., Oluwemimo A., Haslinger E., Bauer R. (2009). Design and synthesis of ten biphenyl-neolignan derivatives and their in vitro inhibitory potency against cyclooxygenase-1/2 activity and 5-lipoxygenase-mediated LTB4-formation. Bioorg. Med. Chem..

[42] Hu Y., Shen Y.F., Tu X., Wu X.H., Wang G.X., Ling F. (2019). Isolation of anti-*Saprolegnia* lignans from *Magnolia officinalis* and SAR evaluation of honokiol/magnolol analogs. Bioorg. Med. Chem. Lett..

[43] Tanaka T., Sakurai Y., Okazaki H., Hasegawa T., Fukuyama Y. (1991). Biphenyl Derivative Composition for Nerve Cell Degeneration Repairing or Protective Agent and Process for Preparing a Phenyl Derivative Contained in the Composition. U.S. Patent.

[44] Bovicelli P., Antonioletti R., Mancini S., Causio S., Borioni G., Annnendola S., Barontini M. (2007). Expedient synthesis of hydroxytyrosol and its esters. Synt. Commun..

[45] Ichikawa T., Netsu M., Mizuno M., Mizusaki T., Takagi Y., Sawama Y., Monguchi Y., Sajiki H. (2017). Development of a unique heterogeneous palladium catalyst for the Suzuki–Miyaura reaction using (hetero)aryl chlorides and chemoselective hydrogenation. Adv. Synth. Catal..

[46] Brunel J.M. (2007). Scope, limitations and mechanistic aspects in the selective homogeneous palladium-catalyzed reduction of alkenes under transfer hydrogen conditions. Tetrahedron.

[47] Freudenberg K., Renner K.C. (1965). Biphenyls and diaryl ethers among the precursors of lignin. Chem. Ber..

[48] Zhang J., Sun T.J., Diao H.P., Li M.P. (2011). Quantitative structure-activity relationship studies of phenol’s biological activity. Shanxi Daxue Xuebao.

[49] Accardo A., Del Zoppo L., Morelli G., Condorelli D.F., Barresi V., Musso N., Spampinato G., Bellia F., Tabbi G., Rizzarelli E. (2016). Liposome antibody-ionophore conjugate antiproliferative activity increases by cellular metallostasis alteration. Med. Chem. Comm..

[50] Scherf U., Ross D.T., Waltham M., Smith L.H., Lee J.K., Tanabe L., Kohn K.W., Reinhold W.C., Myers T.G., Andrews D.T. (2000). A gene expression database for the molecular pharmacology of cancer. Nat. Genet..

