# Supplementary material for: Synthesis of Bisphenol Neolignans Inspired by Honokiol as Antiproliferative Agents
Source: Molecules. 2020 Feb 7;25(3):733. doi: 10.3390/molecules25030733 (PMC7037512; doi:10.3390/molecules25030733)
Supplement: Supplementary file 1 [file molecules-25-00733-s001.pdf]

Supplementary Material

Synthesis of bisphenol neolignans inspired by honokiol as antiproliferative agents

Nunzio Cardullo [1,*], Vincenza Barresi [2], Vera Muccilli [1], Giorgia Spampinato [2], Morgana D'Amico [2] Daniele Filippo Condorelli [2] and Corrado Tringali [1,*]

1 Dipartimento di Scienze Chimiche, University of Catania, Viale A. Doria 6, 95125-Catania, Italy; v.muccilli@unict.it (V.M.)

2 Department of Biomedical and Biotechnological Sciences, Section of Medical Biochemistry, University of Catania, Via Santa Sofia 97, 95123-Catania, Italy; vincenza.barresi@unict.it (V.B.), giorgiaspampinato@unict.it (G.S.), morganadamico01@gmail.com (M.D.), daniele.condorelli@unict.it (D.F.C.)

* Correspondence: ctringali@unict.it (C.T.); ncardullo@unict.it (N.C.); tel: +39-095-7385025 (C.T.).

Preliminary experiments for bromination	1
Preliminary experiments for Suzuki Miyaura reaction	1
HRESIMS and NMR spectra of compound **6**	2
HRESIMS and NMR spectra of compound **12a**	3
HRESIMS and NMR spectra of compound **12b**	6
HRESIMS and NMR spectra of compound **12c**	8
HRESIMS and NMR spectra of compound **13a**	11
HRESIMS and NMR spectra of compound **13b**	14
HRESIMS and NMR spectra of compound **13c**	17
HRESIMS and NMR spectra of compound **14a**	21
HRESIMS and NMR spectra of compound **14b**	23
HRESIMS and NMR spectra of compound **14c**	26
HRESIMS and NMR spectra of compound **15a**	29

Preliminary experiments for bromination

2-Methoxy-4-propylphenol (**10a**) has been subjected to preliminary reactions as reported:

1) compound **10a** (21 mg; 126 μmol) was solubilized in CH_3CN (700 μL) and treated with I_2 (7 mg; 20%) and *N*-bromosuccinimide (NBS; 31.5 mg; 175 μmol) at rt;

2) compound **10a** (21 mg; 126 μmol) was solubilized in $CHCl_3$ (700 μL) and treated with I_2 (7 mg; 20%) and NBS (31.5 mg; 175 μmol) at rt;

3) compound **10a** (21 mg; 126 μmol) was solubilized in CH_3CN (700 μL) and treated with $AlCl_3$ (7 mg; 40%) NBS (31 mg; 170 μmol) at rt;

4) compound **10a** (21 mg; 126 μmol) was solubilized in $CHCl_3$ (700 μL) and treated with $AlCl_3$ (7 mg; 40%) and NBS (31.5 mg; 175 μmol) at rt;

5) the phenol **10a** (21 mg; 126 μmol) solubilized in CH_3CN (700 μL) was mixed with $AlCl_3$ (7 mg; 40%) and Br_2 (10 μL; 210 μmol) at rt;

6) the phenol **10a** (21 mg; 126 μmol) was solubilized in $CHCl_3$ (700 μL) and treated with Br_2 (10 μL; 210 μmol) at rt;

7) the phenol **10a** (21 mg; 126 μmol) was solubilized in $CHCl_3$ (700 μL) and treated with Br_2 (10 μL; 210 μmol) at 0 °C;

8) the phenol **10a** (21 mg; 126 μmol) was solubilized in acetone (500 μL) and treated with NaBr (26.1 mg; 252 μmol) and a solution of oxone (100.2 mg) in water (500 μL) at -10 °C.

The mixtures were stirred at room temperature and monitored by TLC (85:15 *n*-hexane/acetone) for 6 h. Then each mixture was diluted with CH_2Cl_2 (1 mL) and partitioned with a saturated $Na_2S_2O_3$ solution. The organic layer of experiment 7 was purified on silica gel column chromatography (cyclohexane:EtOAc 98:2 → cyclohexane:EtOAc 96:4) to give **11a**. The pure product was used to create a calibration curve via HPLC-UV, to determine the yield of other reactions (see Table 1).

Preliminary experiments for Suzuki-Miyaura cross-coupling

Preliminary experiments for S-M reaction were performed employing compound **11a** as starting material (8.3 mg, 50 μmol) in presence of 4-hydroxyphenylboronic acid (10.3 mg, 75 μmol), dppf (8.3 mg, 15 μmol), $Pd(OAc)_2$ (1.1 mg, 5 μmol) as catalyst and K_2CO_3 (34.6 mg, 250 μmol). The solvent and temperature were varied as reported:

1) the reaction was carried out in THF (500 μL) at 25 °C;
2) the reaction was carried out in THF (500 μL) at 70 °C;
3) the mixture was stirred in THF:H_2O 10:1 (500 μL and 50 μL) at 70 °C;
4) the mixture was stirred in THF:H_2O 10:1 (910 μL and 90 μL) at 70 °C;
5) the reaction was carried out in 1,4-dioxane (500 μL) at 70 °C;
6) the reaction was carried out in 1,4-dioxane (500 μL) at 180 °C.

The course of the reactions was followed by TLC for 24h, then they were partitioned between H_2O:EtOAc (3 x 1 mL). The organic layer obtained from experiment 4) was purified on silica gel column chromatography (petroleum ether → petroleum ether:acetone 92:8) to furnish the product **12a**. The pure bisphenol was used to create a calibration curve via HPLC-UV, to determine the yield of other reactions (see Table 2).

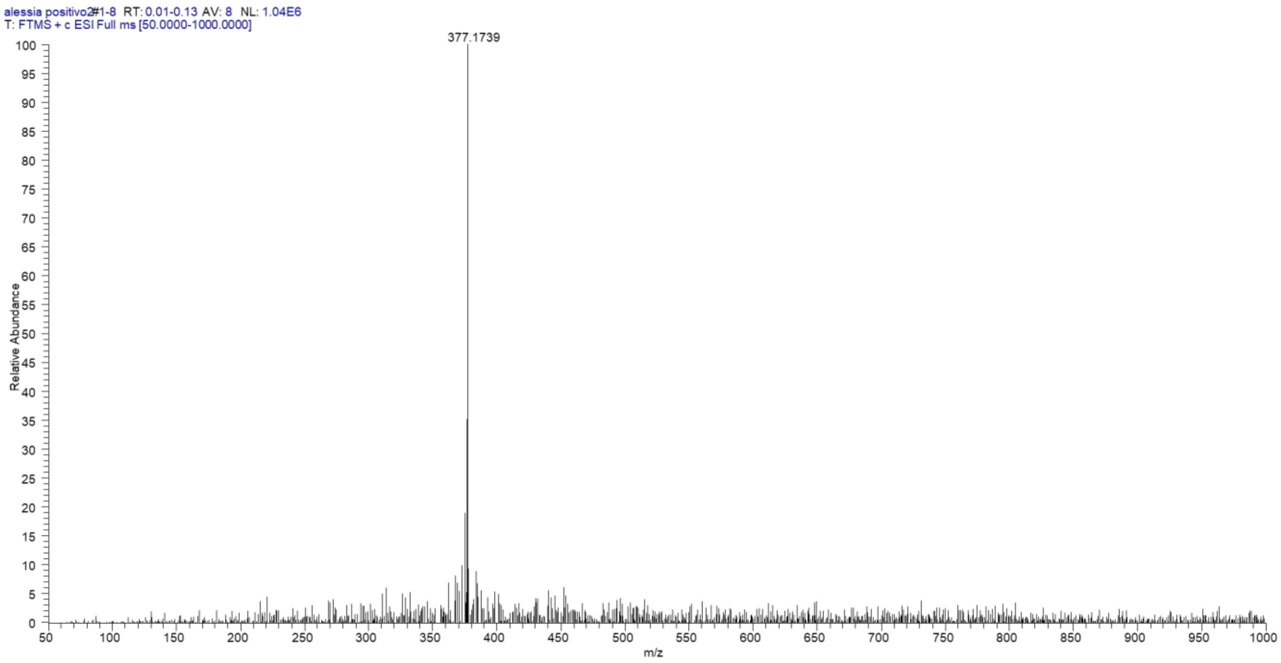

Figure S1. HRESIMS (+) spectrum of **6**.

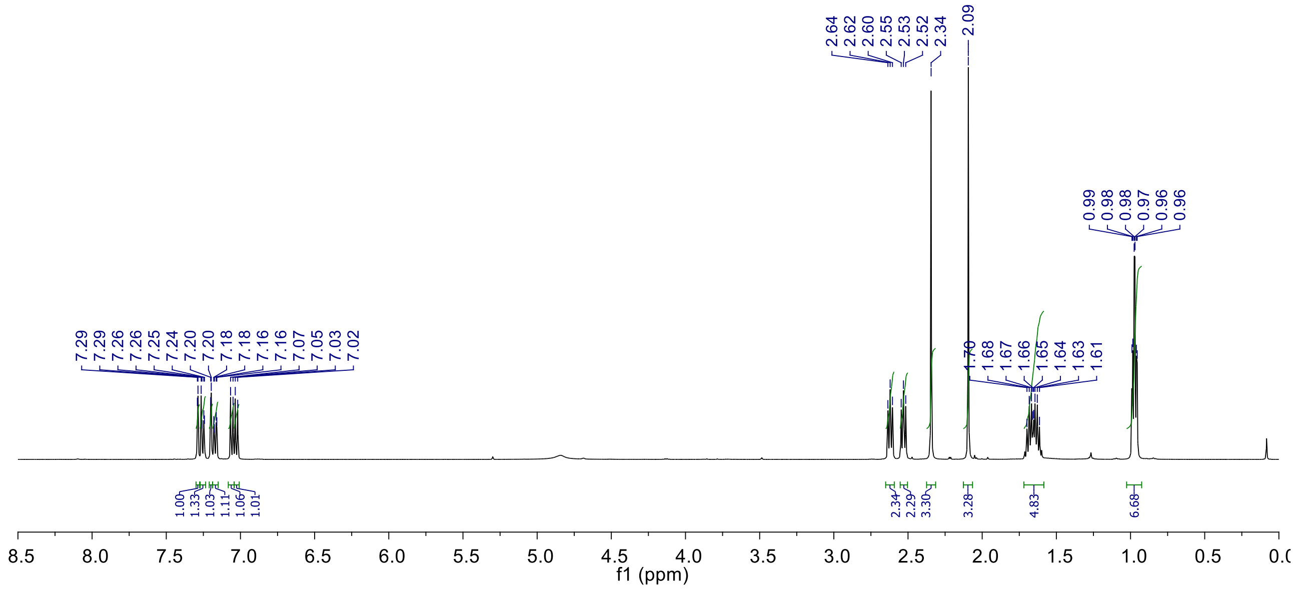

Figure S2. ^{1}H NMR spectrum (500 MHz, $CDCl_3$) of **6**.

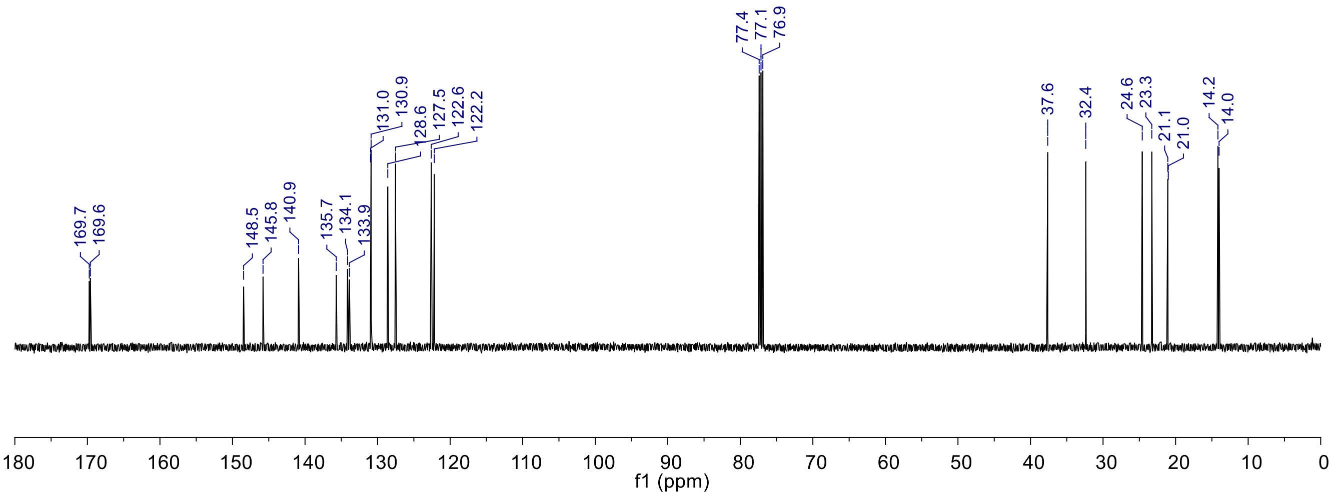

Figure S3. ^{13}C NMR spectrum (125 MHz, $CDCl_3$) of **6**.

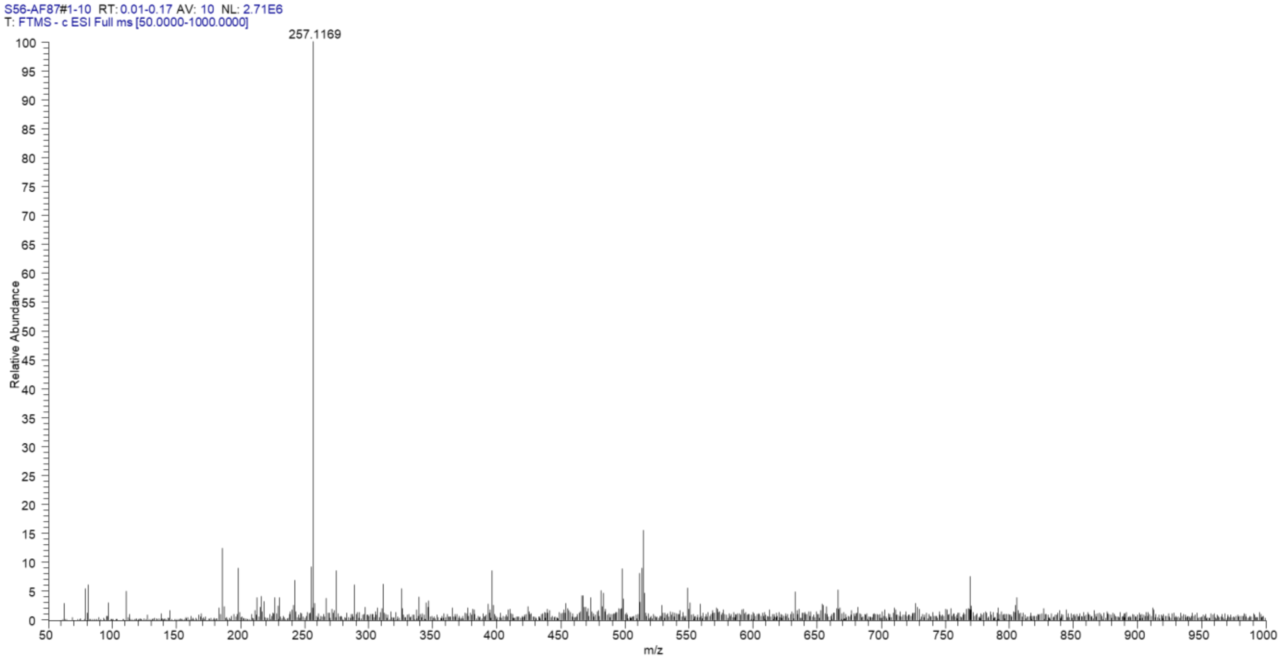

Figure S4. HRESIMS (-) spectrum of **12a**.

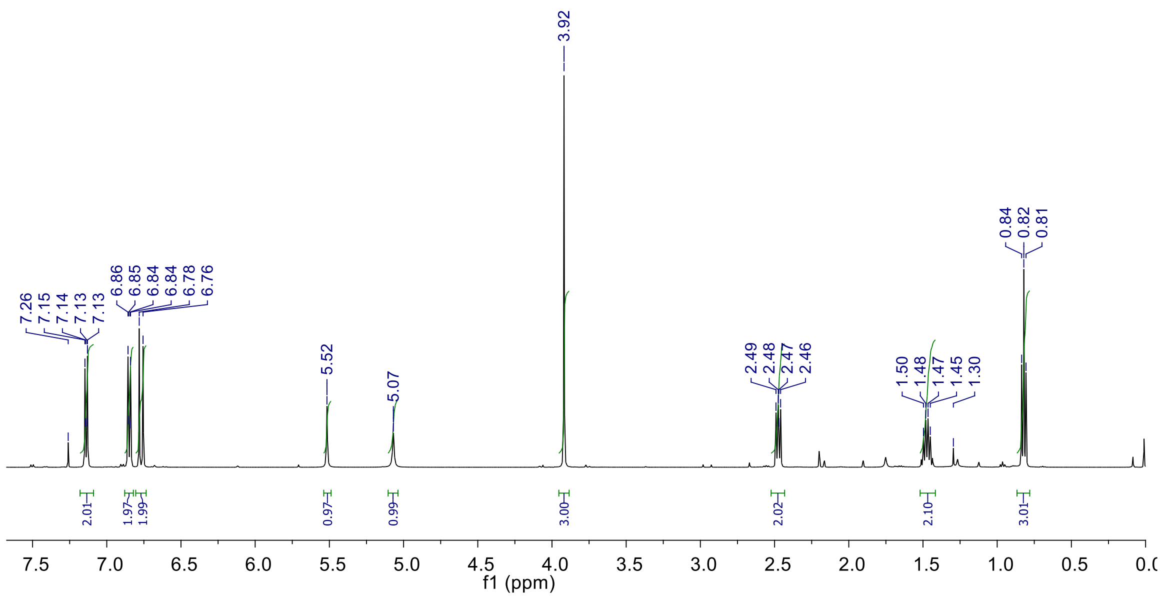

Figure S5. ^{1}H NMR spectrum (500 MHz, $CDCl_3$) of **12a**.

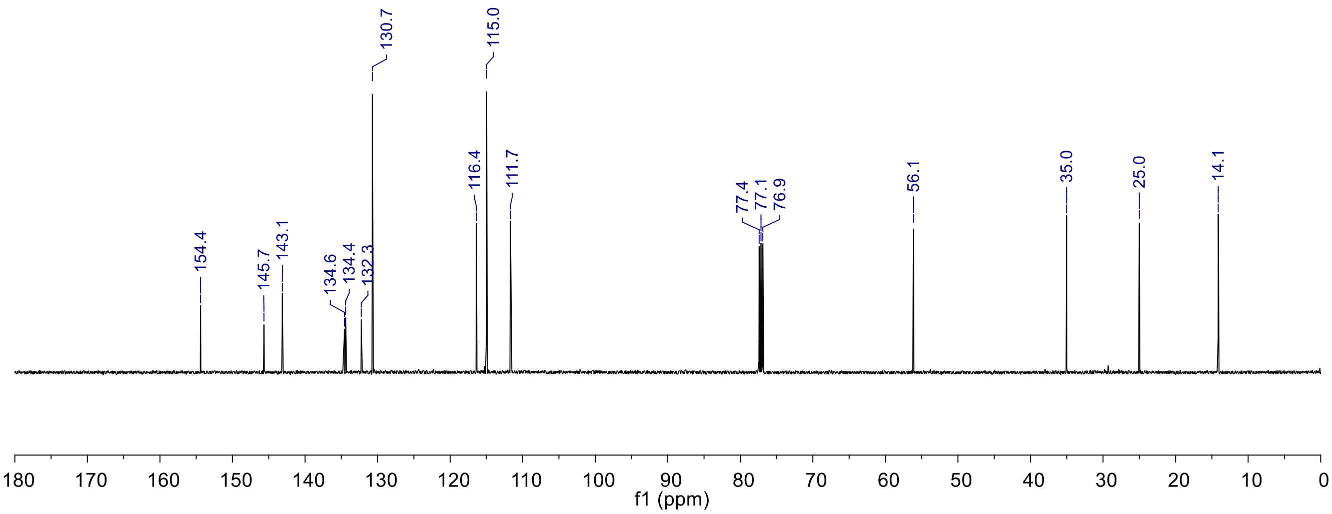

Figure S6. ^{13}C NMR spectrum (125 MHz, $CDCl_3$) of **12a**.

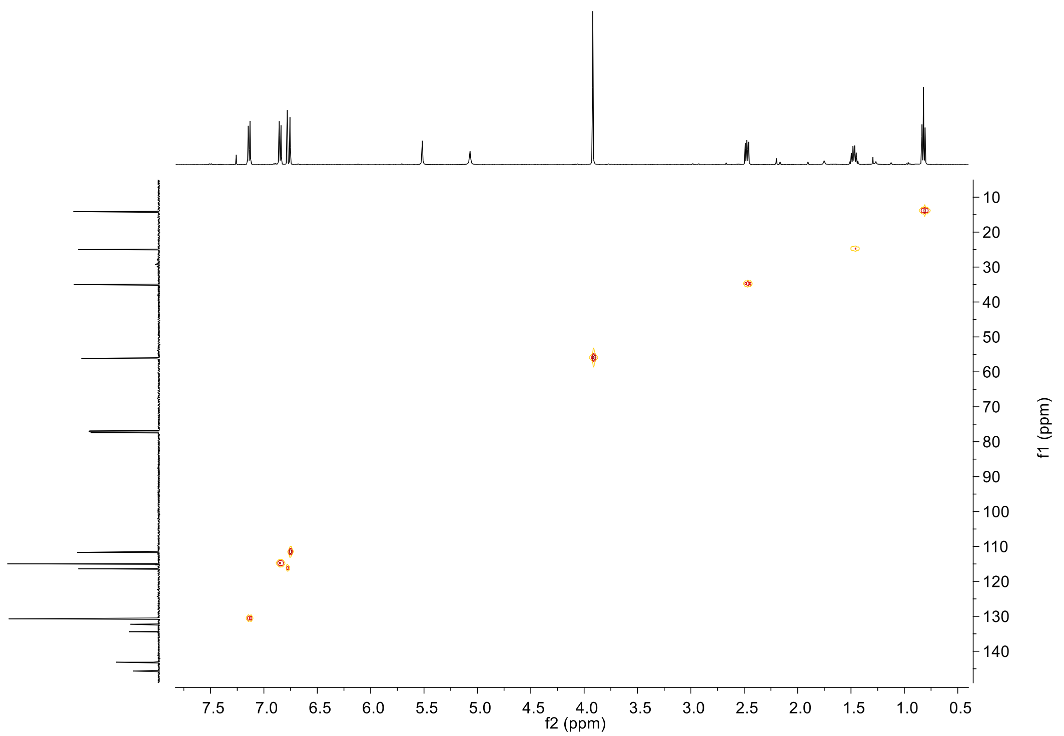

Figure S7. gHSQC spectrum of **12a**.

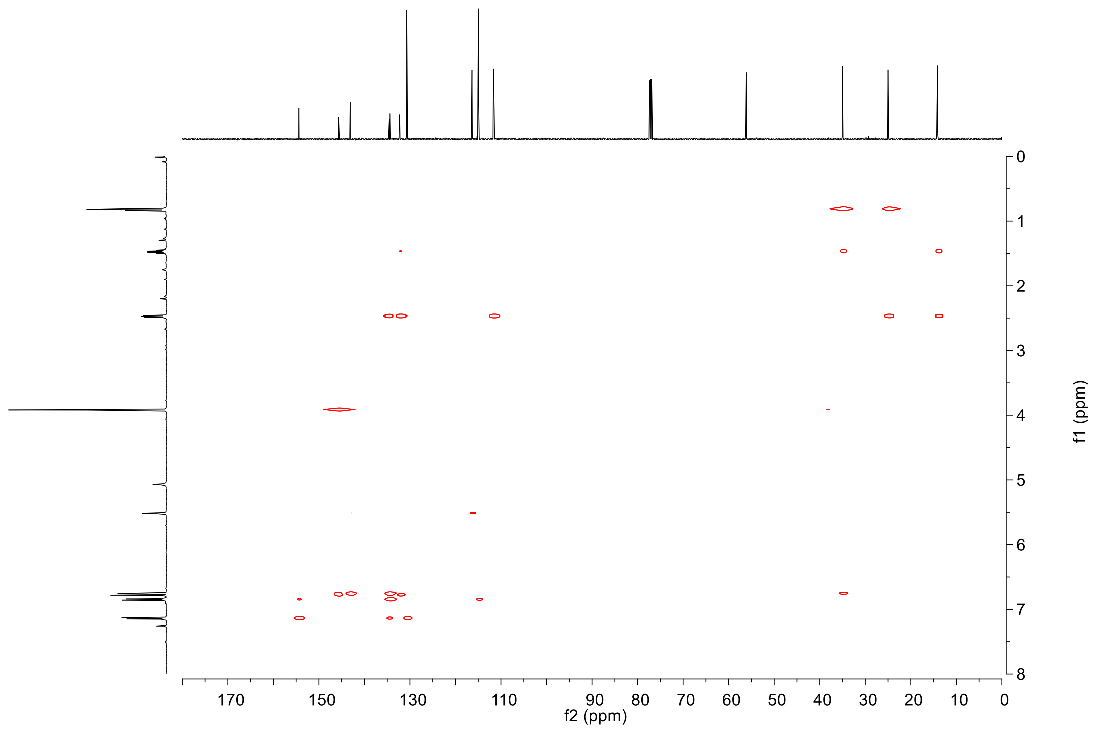

Figure S8. gHMBC spectrum of **12a**.

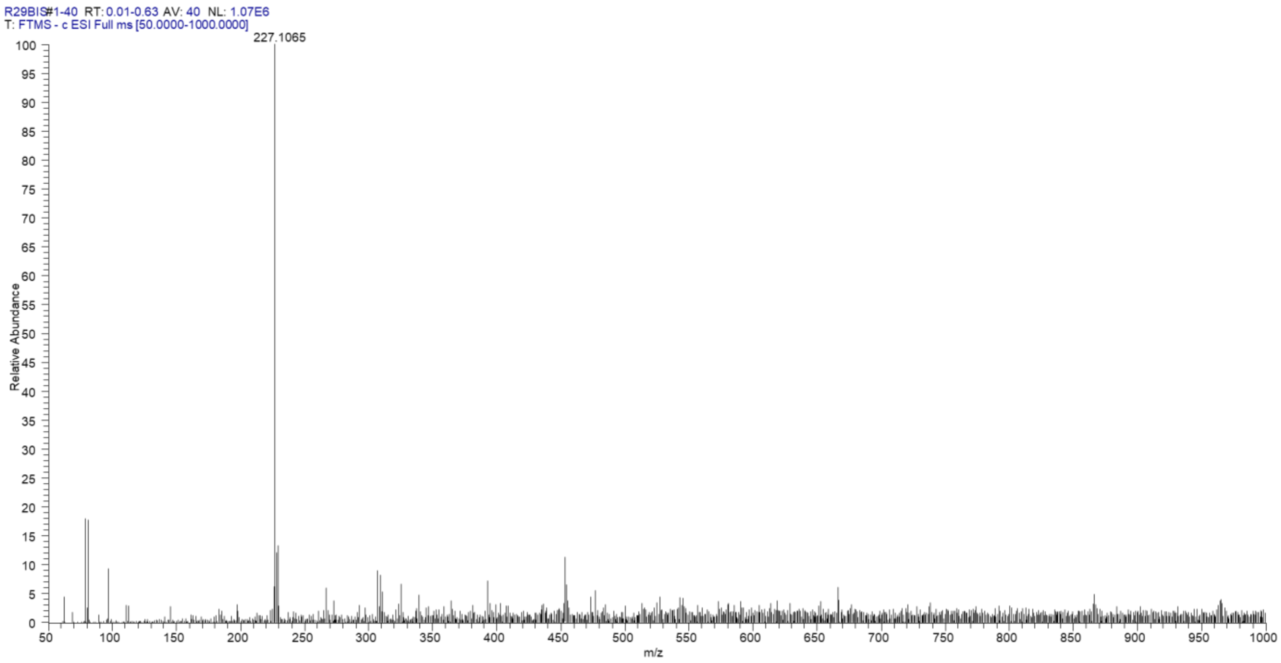

Figure S9. HRESIMS (-) spectrum of **12b**.

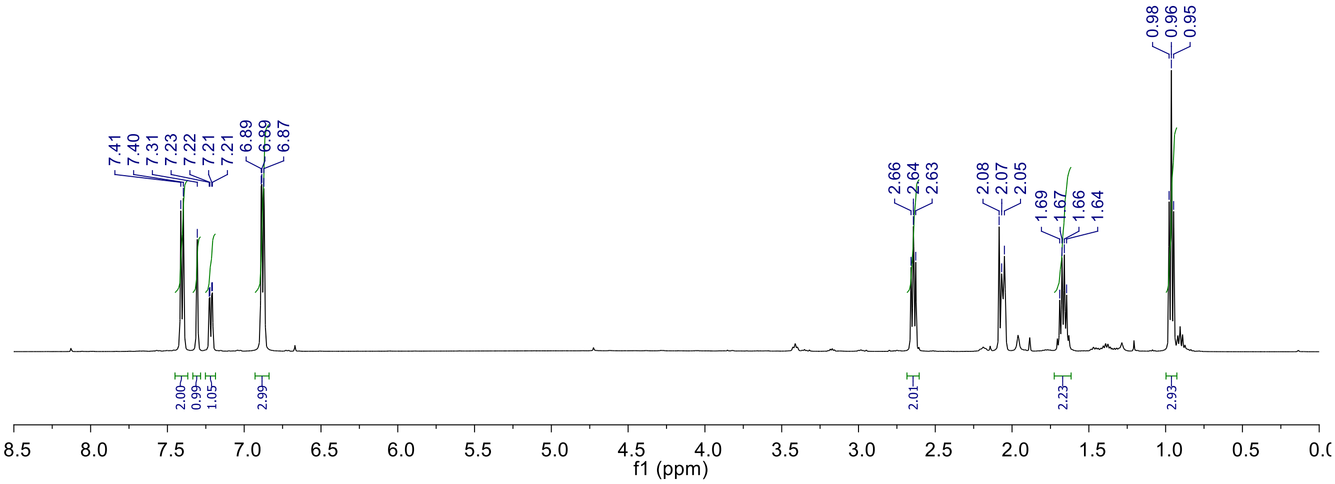

Figure S10. ^{1}H NMR spectrum (500 MHz, $(CD_3)_2CO$) of **12b**.

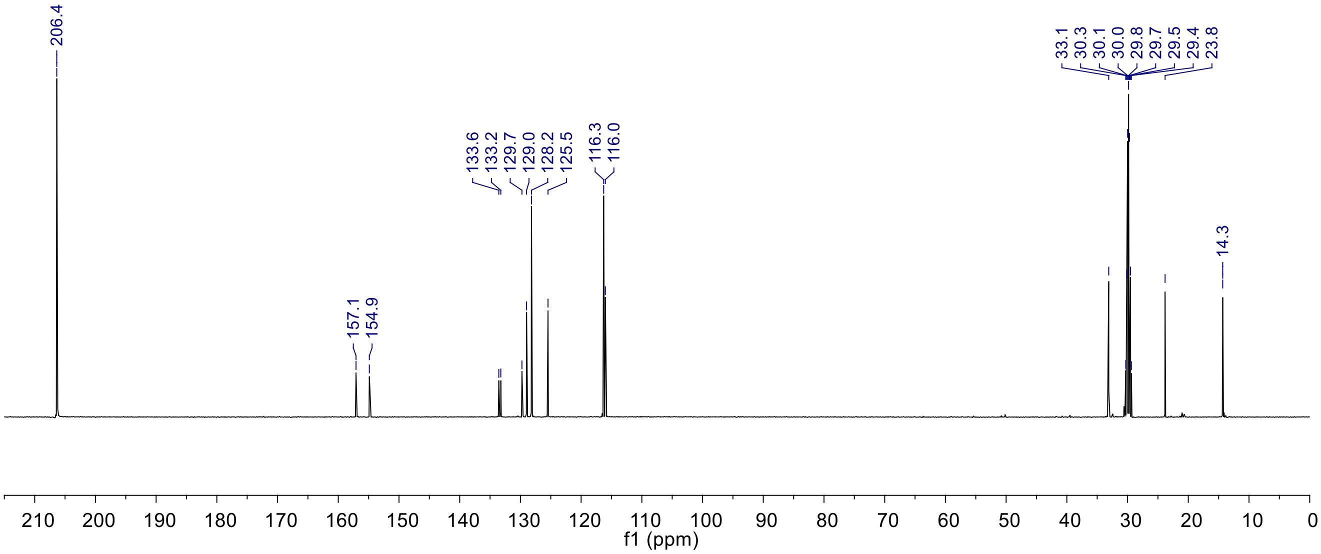

Figure S11. ^{13}C NMR spectrum (125 MHz, $(CD_3)_2CO$) of **12b**.

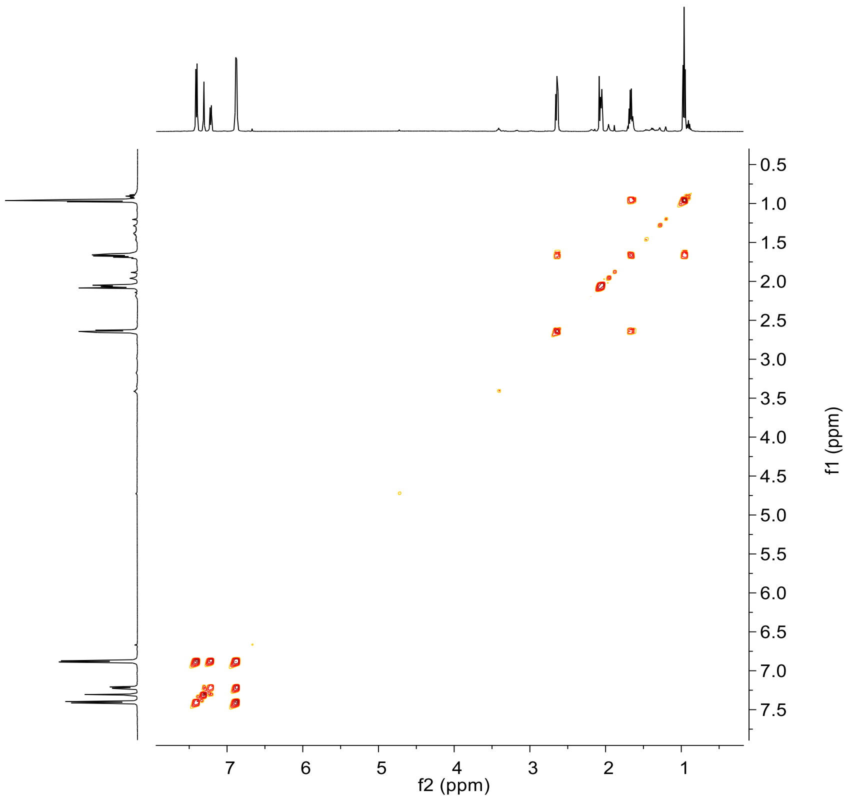

FigureS12. gCOSY spectrum of **12b**.

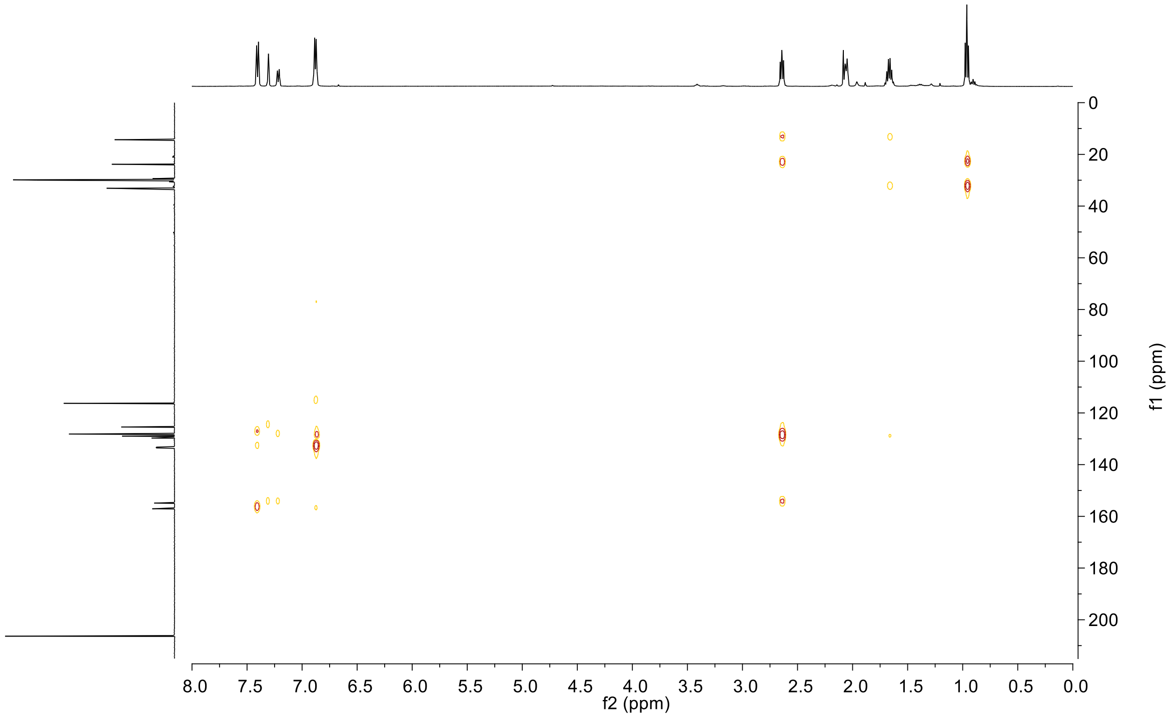

FigureS13. gHMBC spectrum of **12b**.

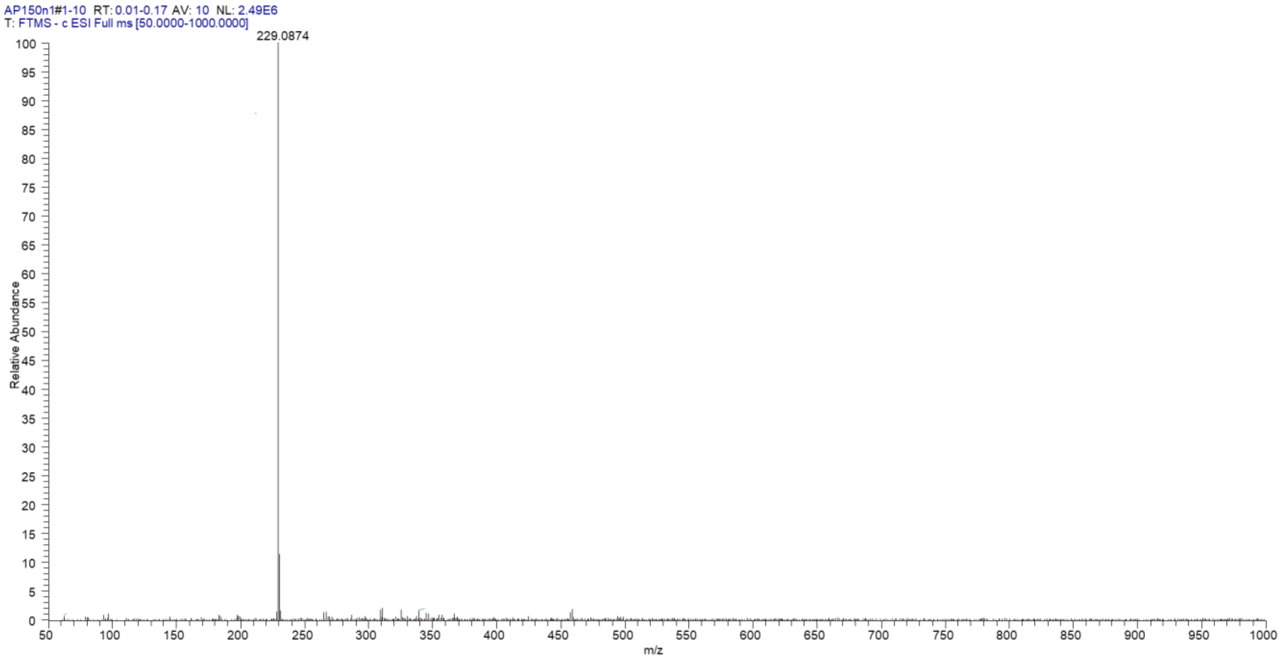

FigureS14. HRESIMS (-) spectrum of **12c**.

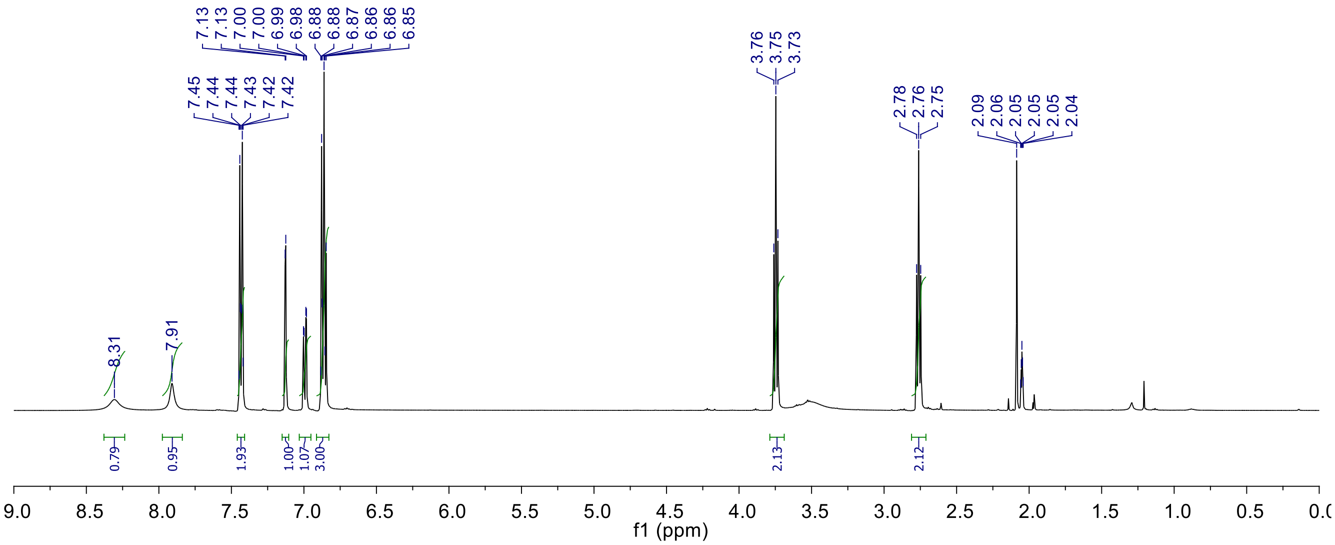

Figure S15. ^{1}H NMR spectrum (500 MHz, $(CD_3)_2CO$) of **12c**.

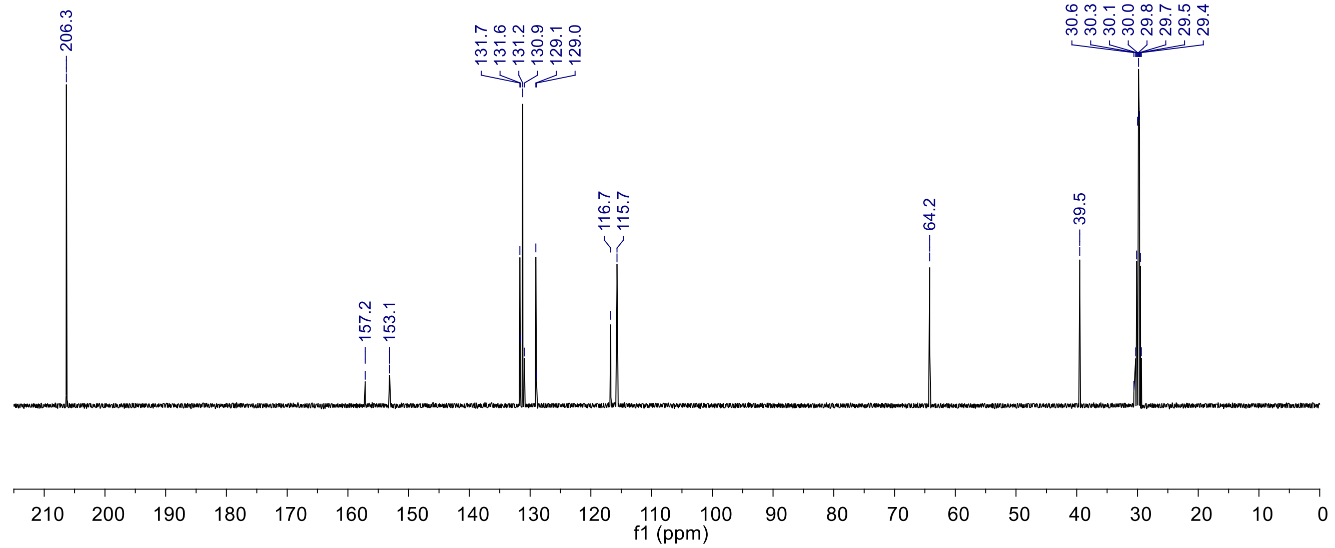

Figure S16. ^{13}C NMR spectrum (125 MHz, $(CD_3)_2CO$) of **12c**.

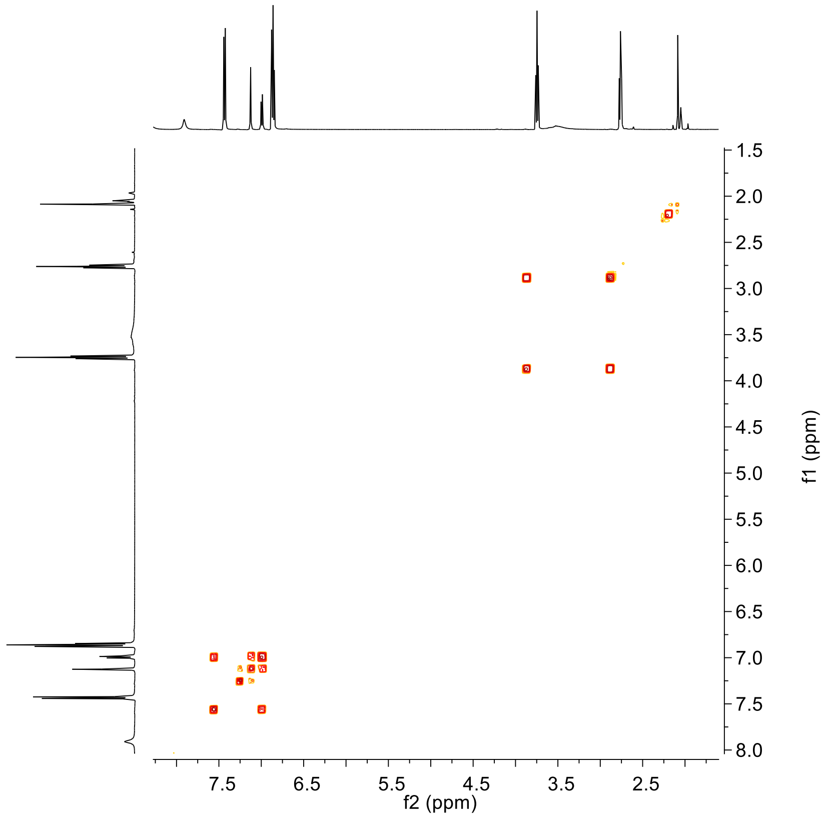

Figure S17. gCOSY spectrum of **12c**.

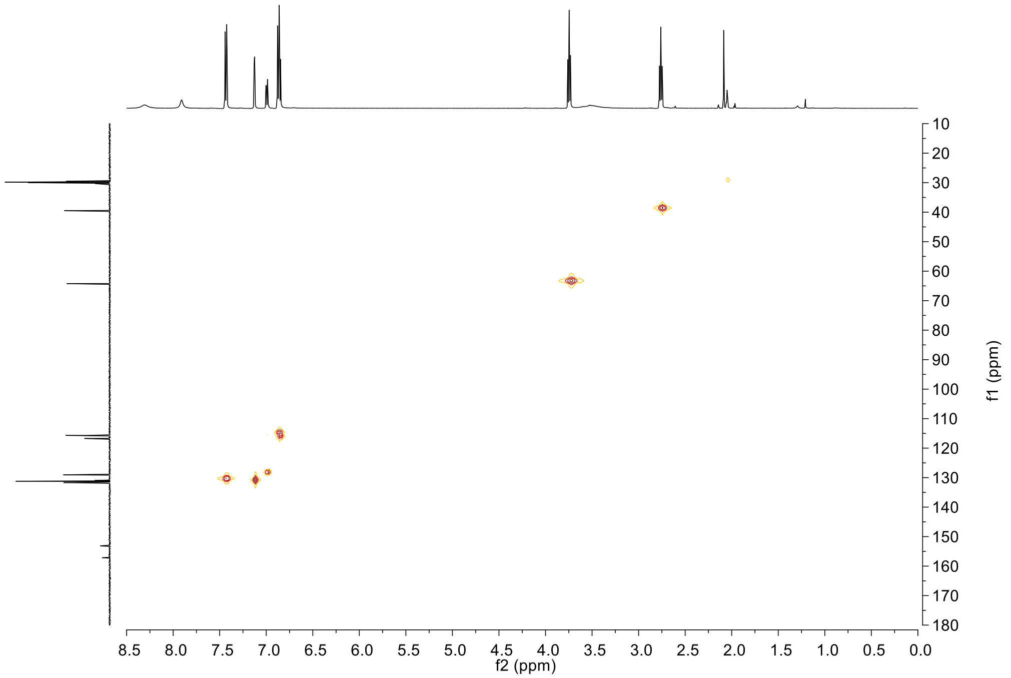

Figure S18. gHSQC spectrum of **12c**.

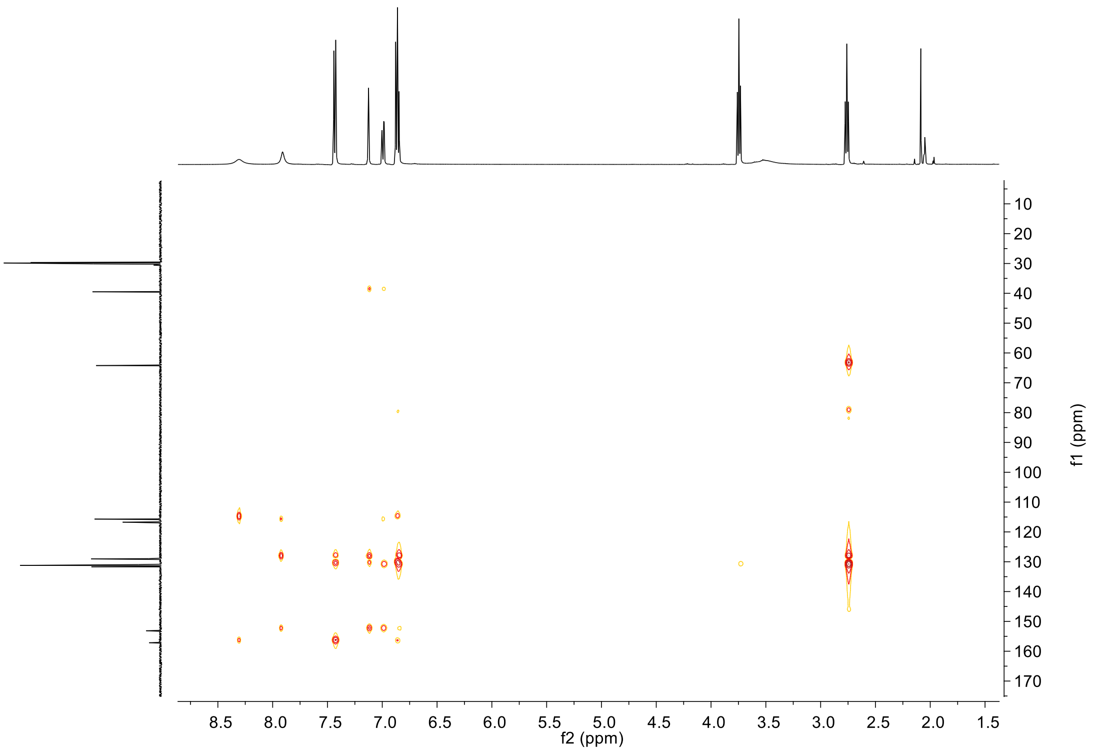

Figure S19. gHMBC spectrum of **12c**.

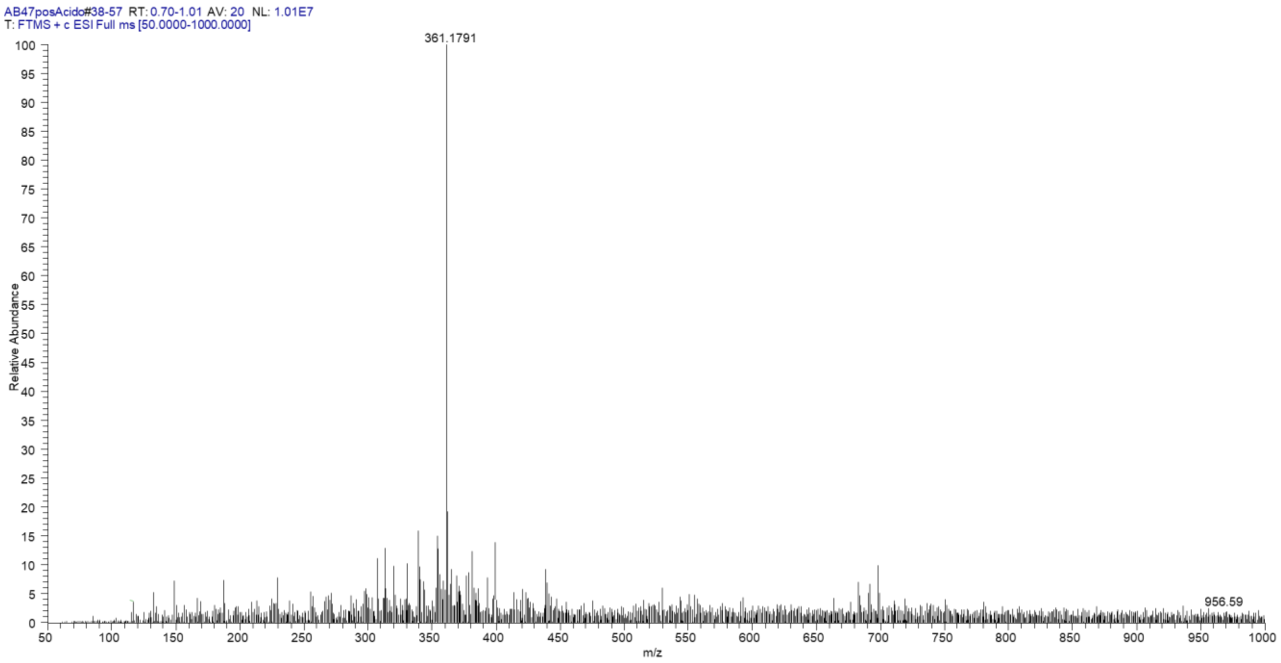

Figure S20. HRESIMS (+) spectrum of **13a**.

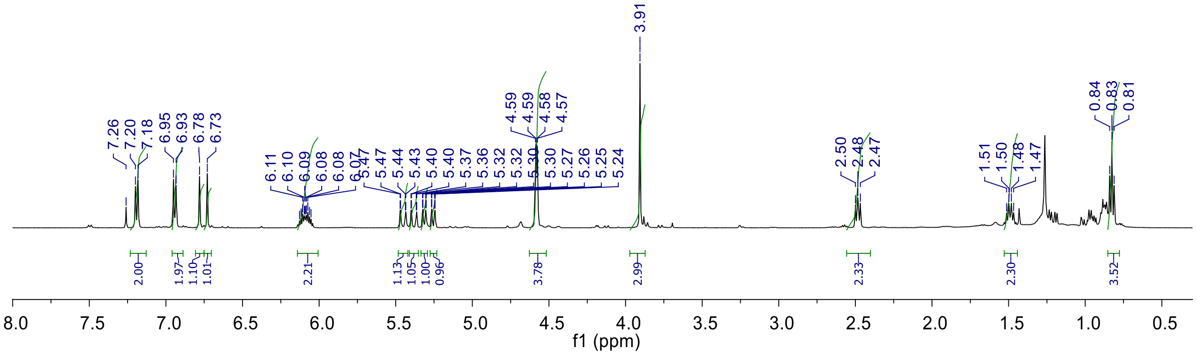

Figure S21. ^{1}H NMR spectrum (500 MHz, $CDCl_3$) of **13a**.

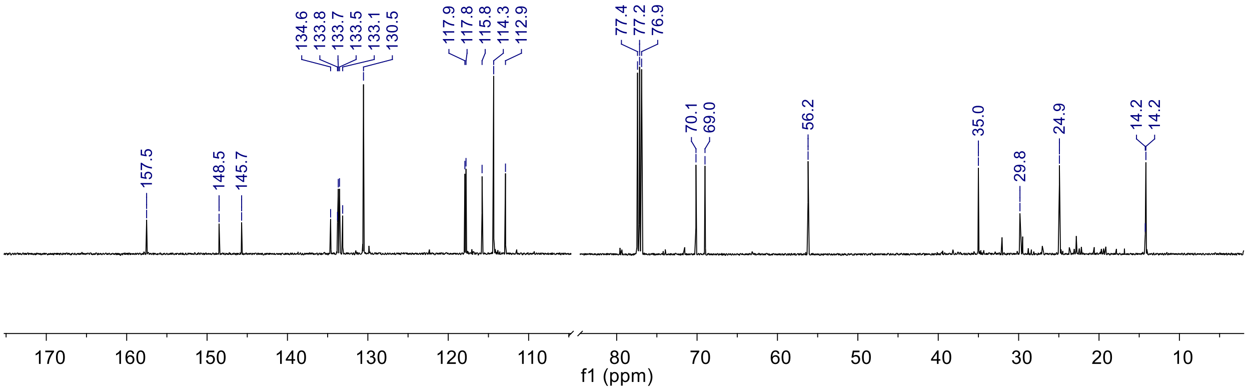

Figure S22. ^{13}C NMR spectrum (125 MHz, $CDCl_3$) of **13a**.

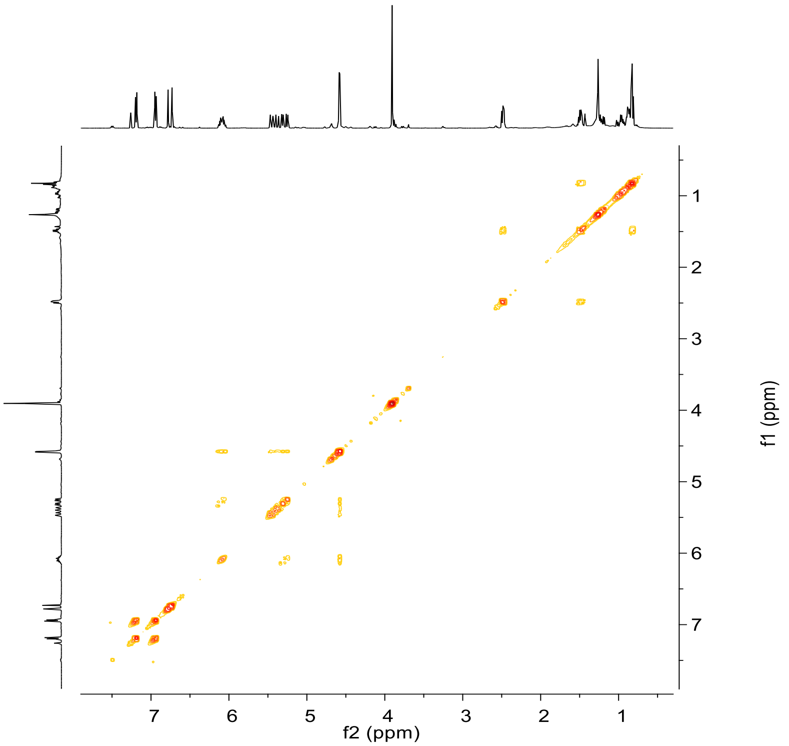

Figure S23. gCOSY spectrum of **13a**.

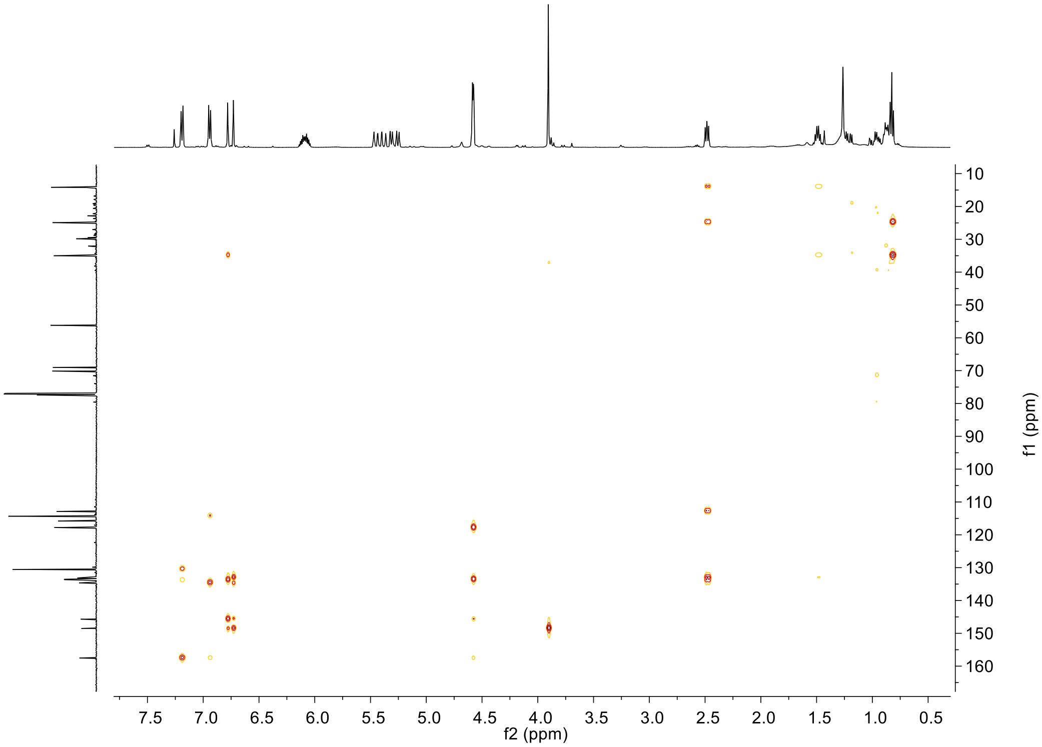

Figure S24 gHMBC spectrum of **13a**.

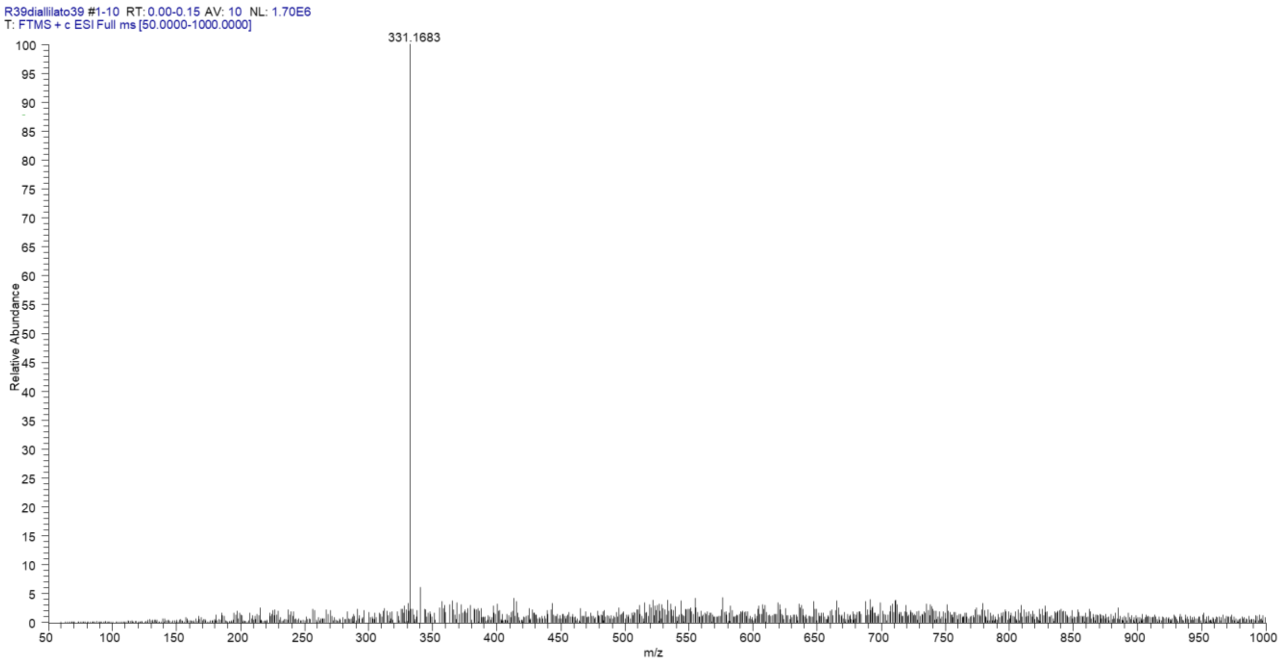

Figure S25 HRESIMS (+) spectrum of **13b**.

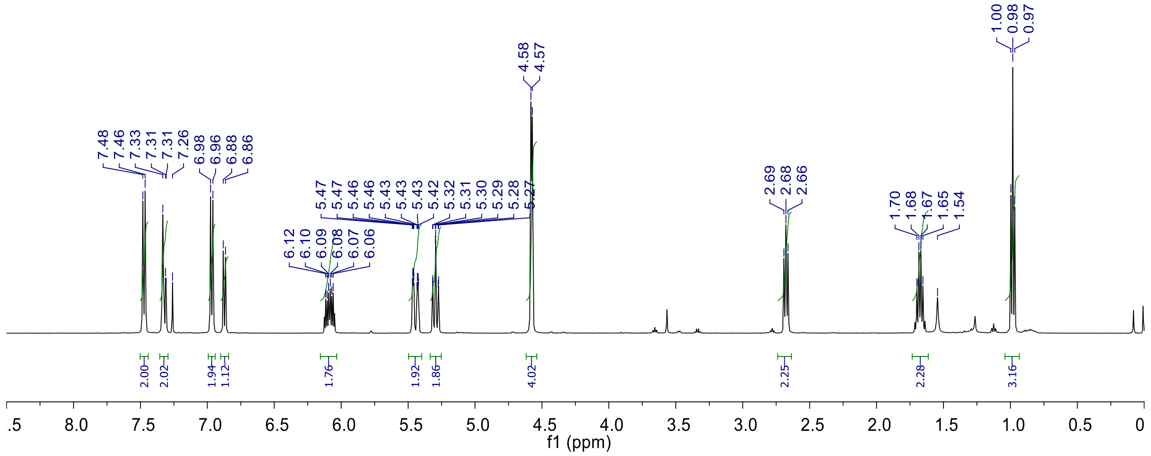

Figure S26. ^{1}H NMR spectrum (500 MHz, $CDCl_3$) of **13b**.

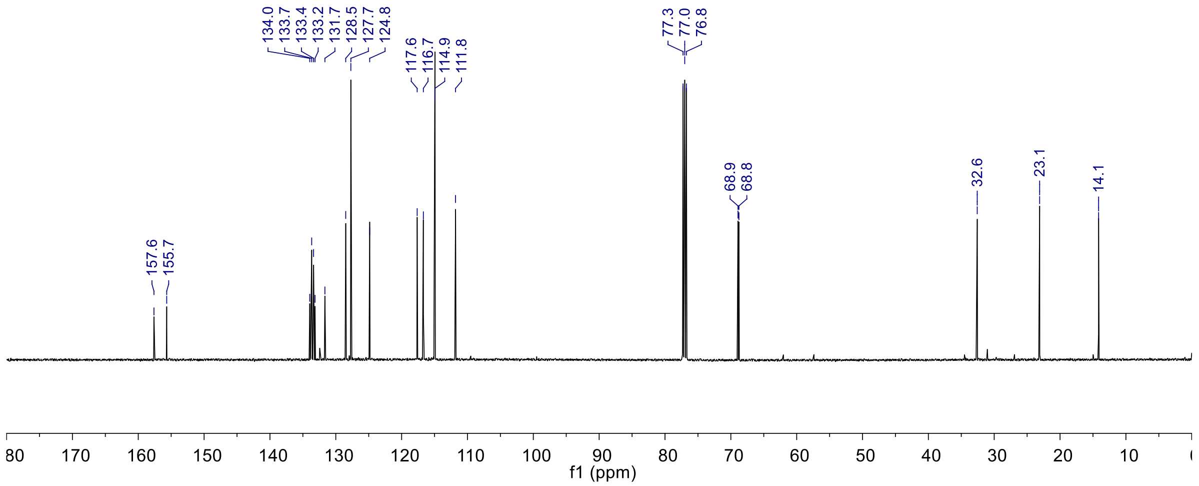

Figure S27. ^{13}C NMR spectrum (125MHz, $CDCl_3$) of **13b**.

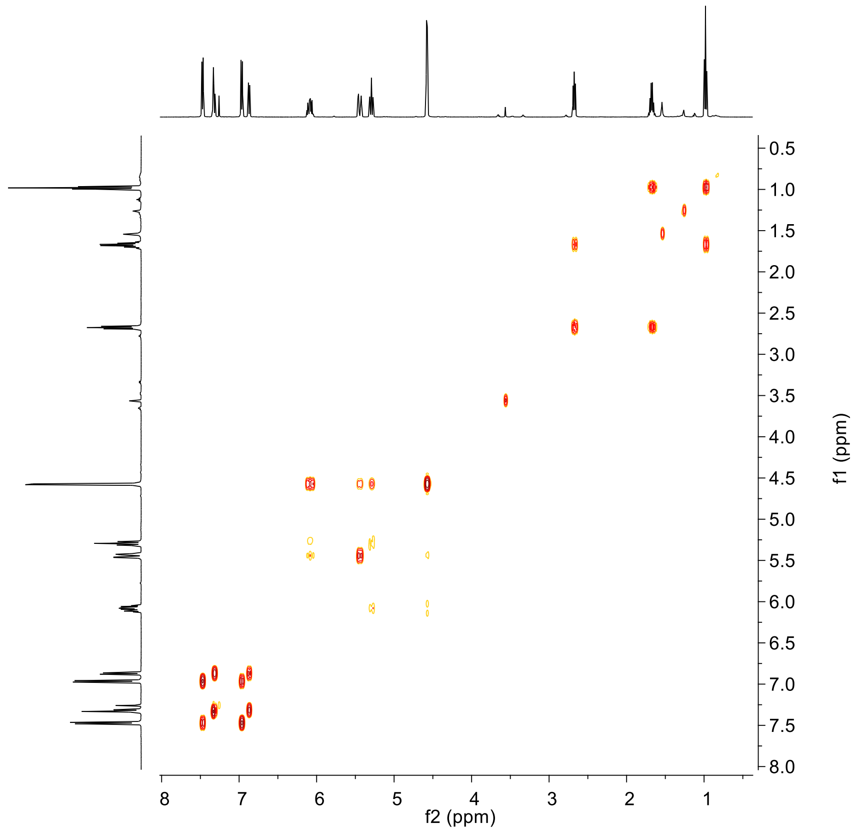

Figure S28. gCOSY spectrum of **13b**.

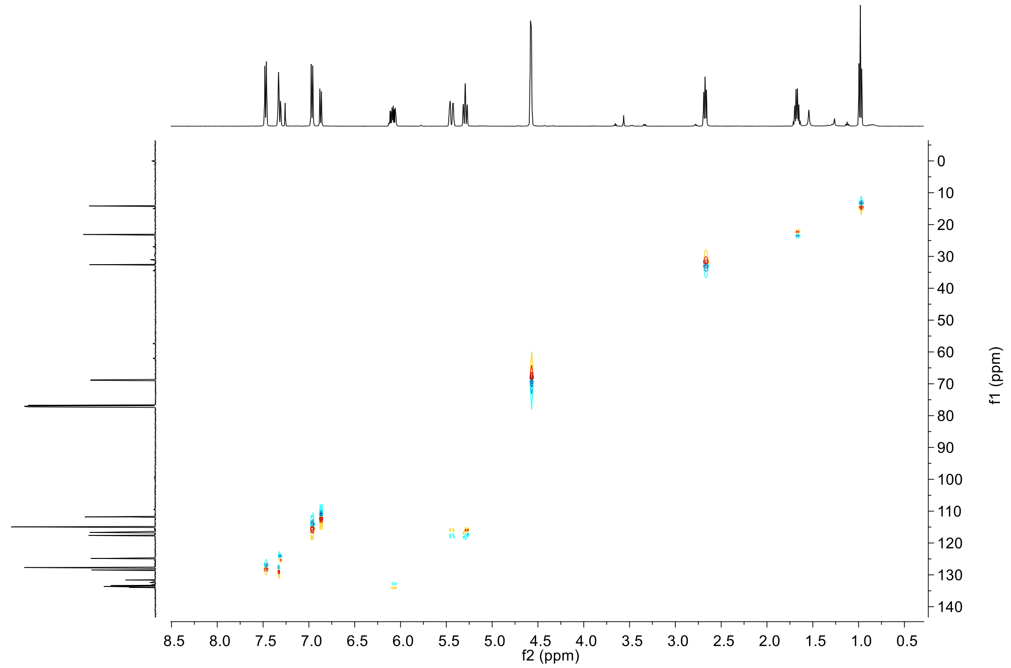

Figure S29. gHSQC spectrum of **13b**.

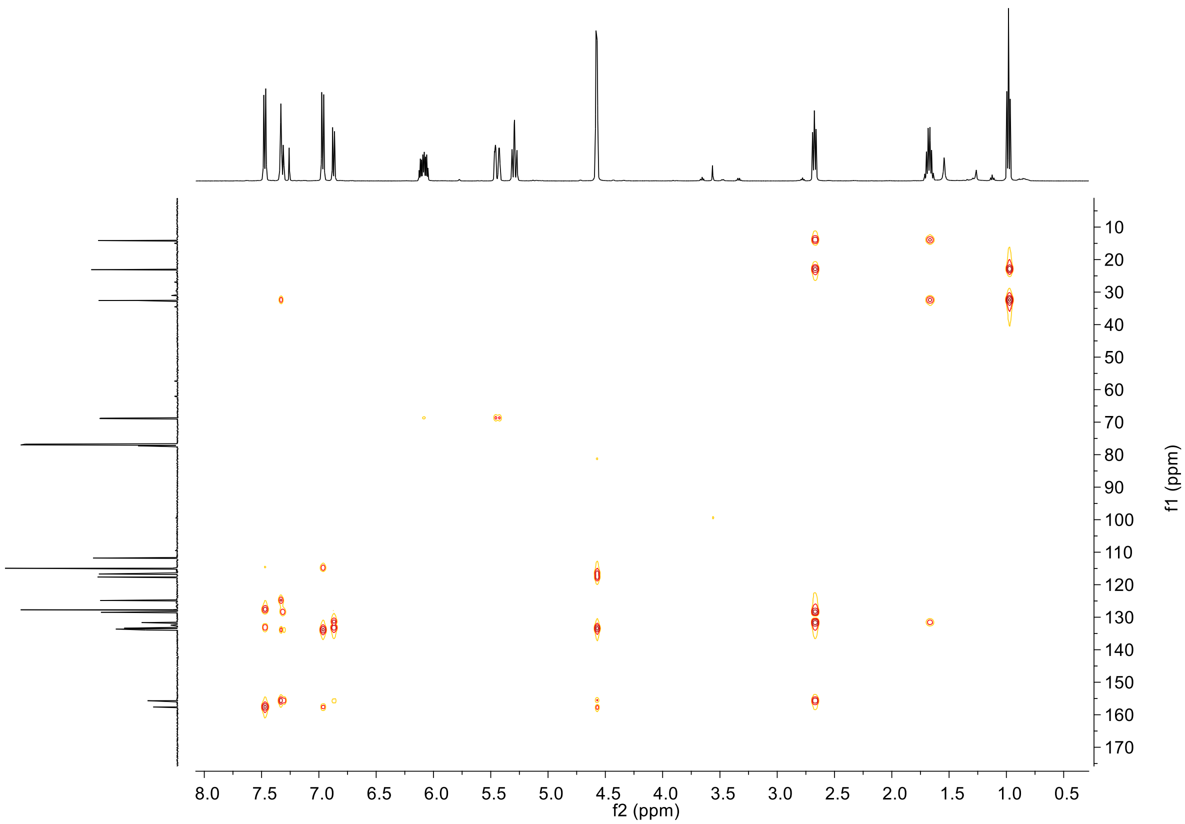

Figure S30. gHMBC spectrum of **13b**.

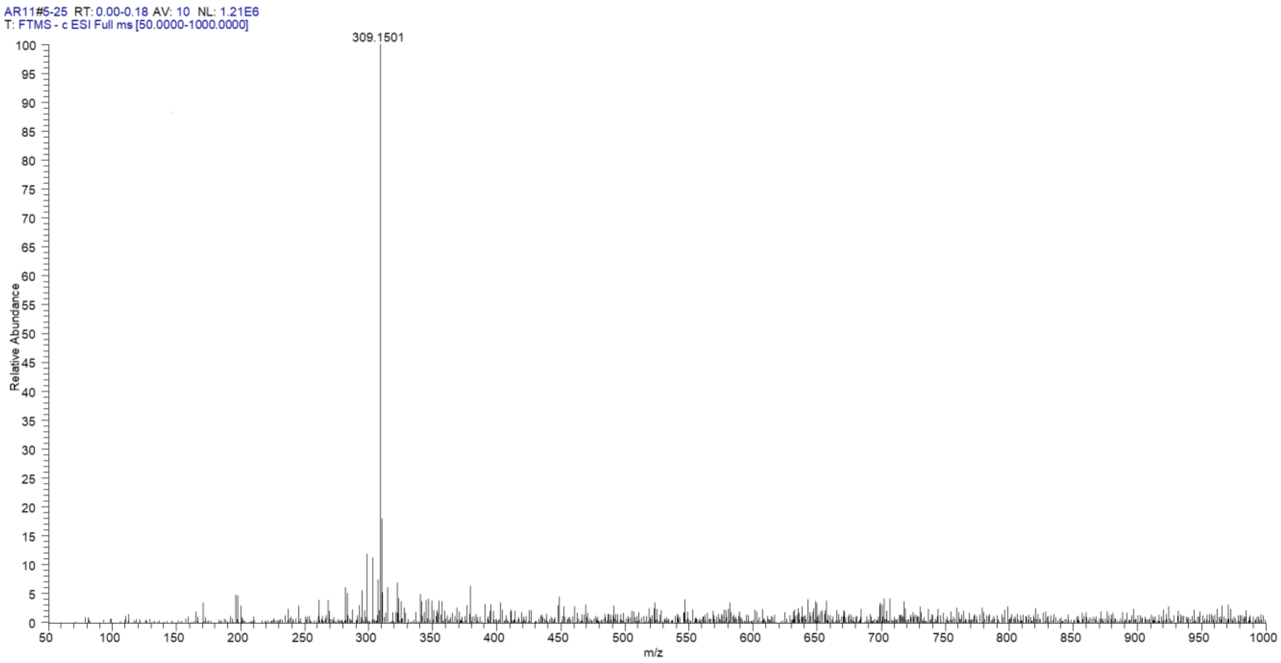

Figure S31. HRESIMS (-) spectrum of **13c**.

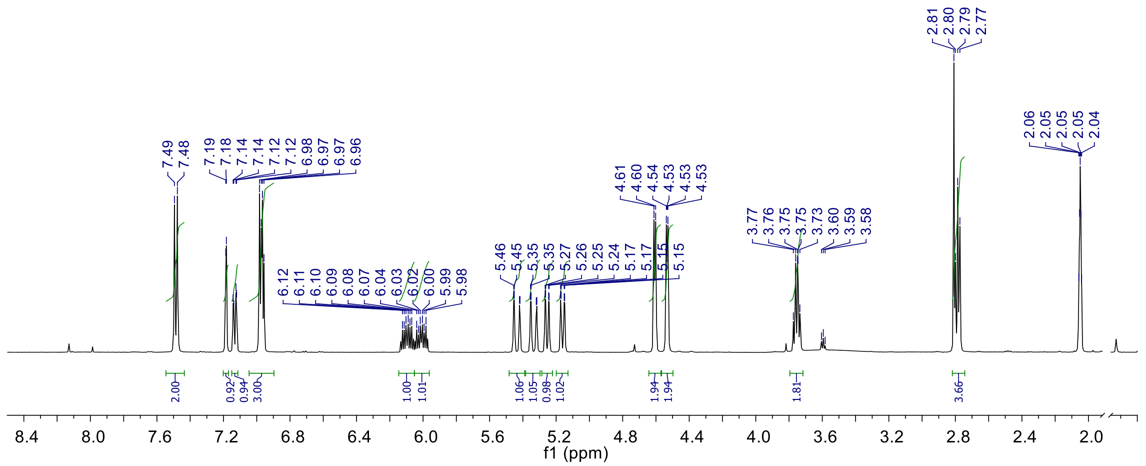

Figure S32. ^{1}H NMR spectrum (500 MHz, $(CD_3)_2CO$) of **13c**.

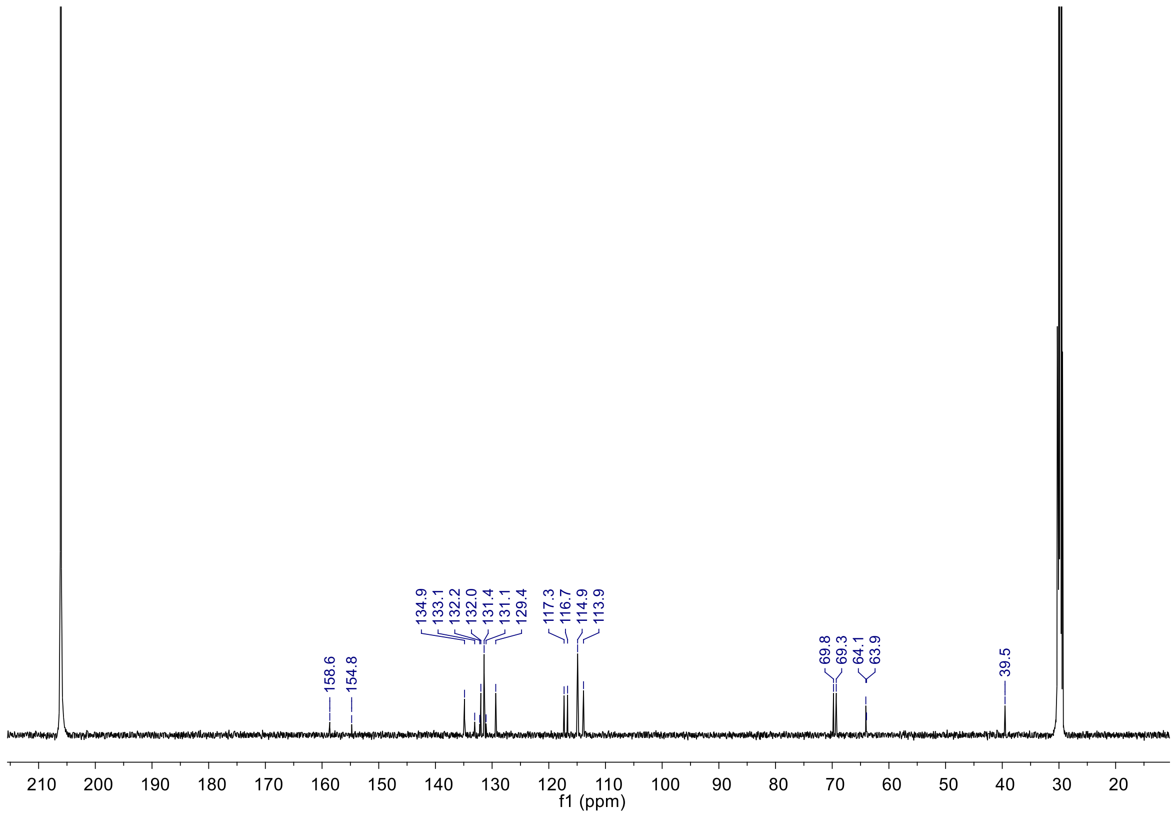

Figure S33. ^{13}C NMR spectrum (125 MHz, $(CD_3)_2CO$) of **13c**.

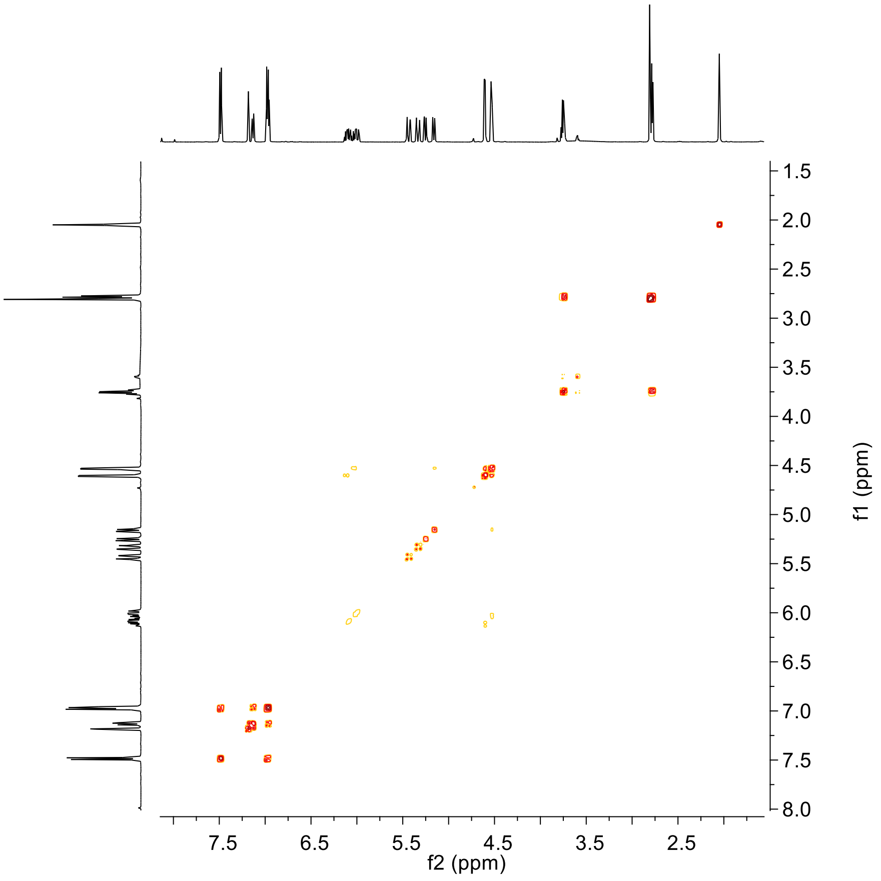

Figure S34. gCOSY spectrum of **13c**.

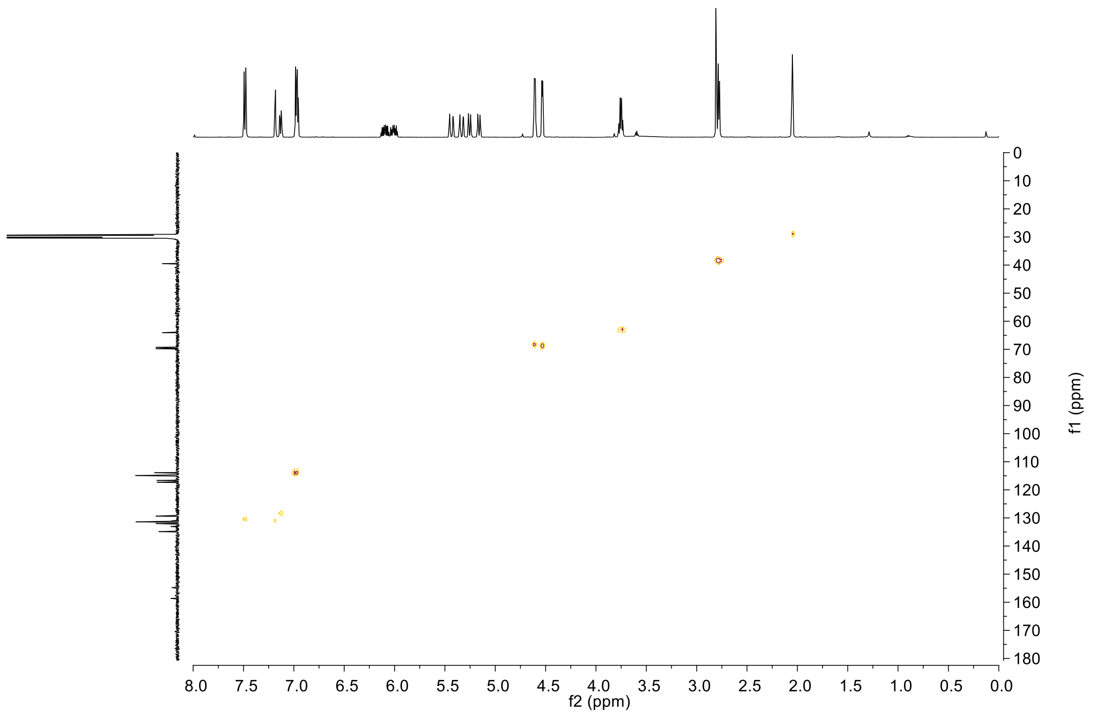

Figure S35. gHSQC spectrum of **13c**.

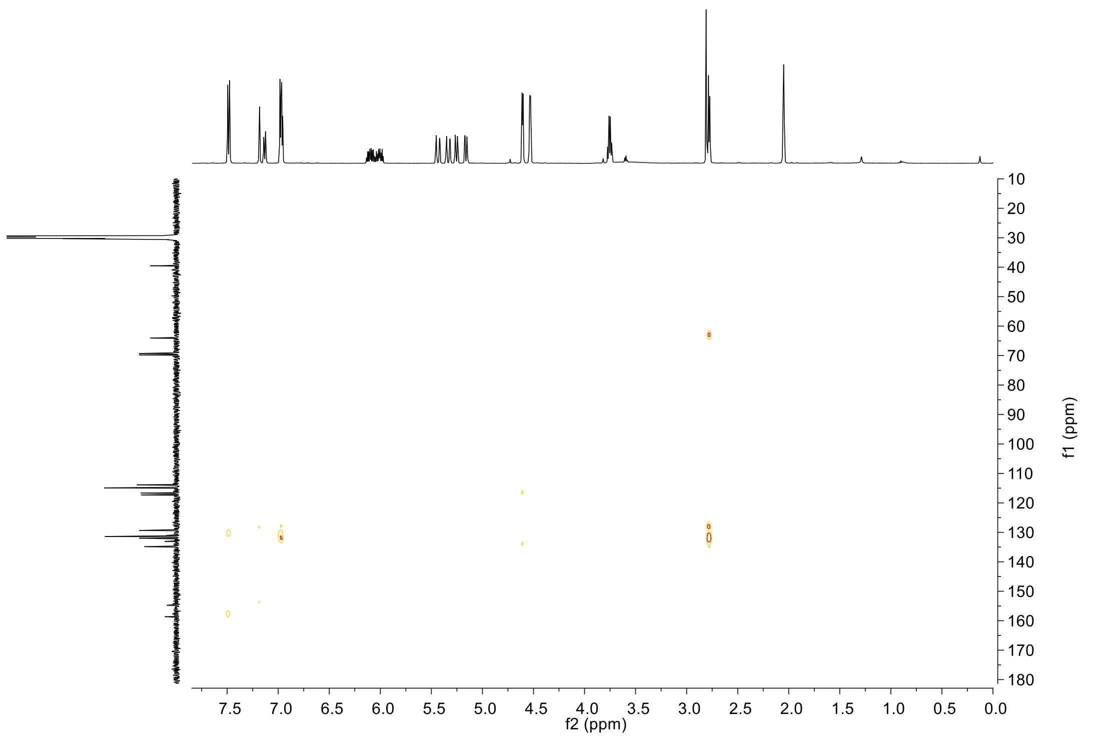

Figure S36. gHMBC spectrum of **13c**.

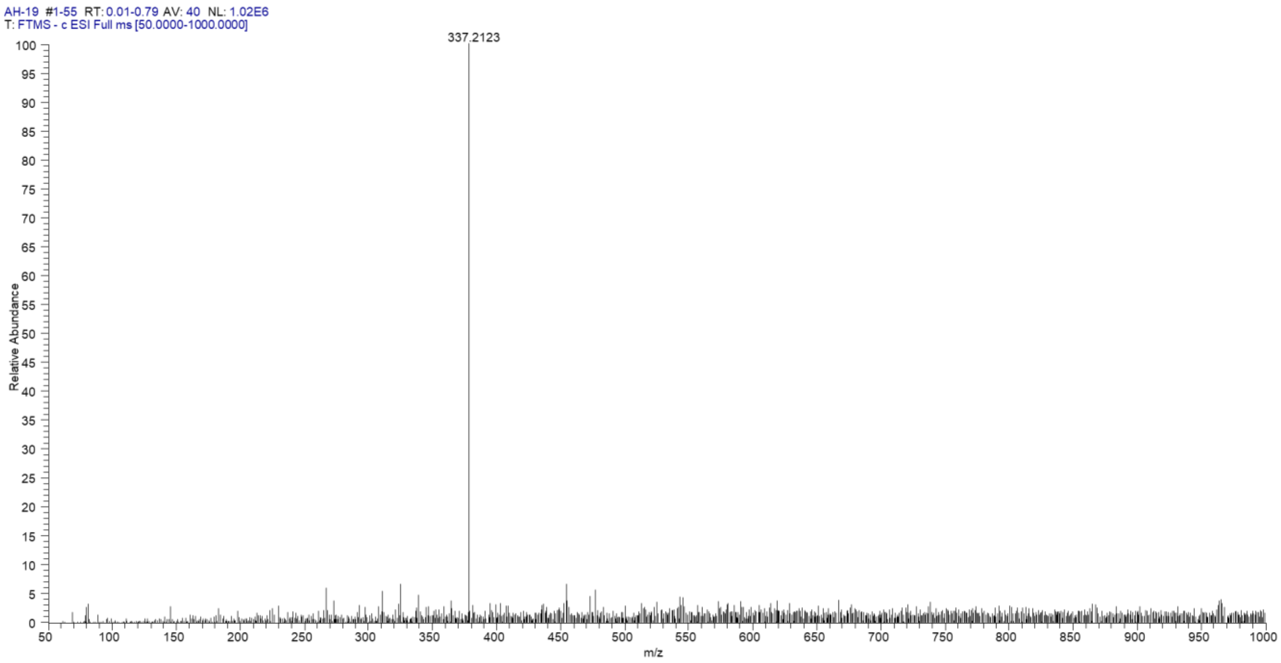

Figure S37. HRESIMS (-) spectrum of **14a**.

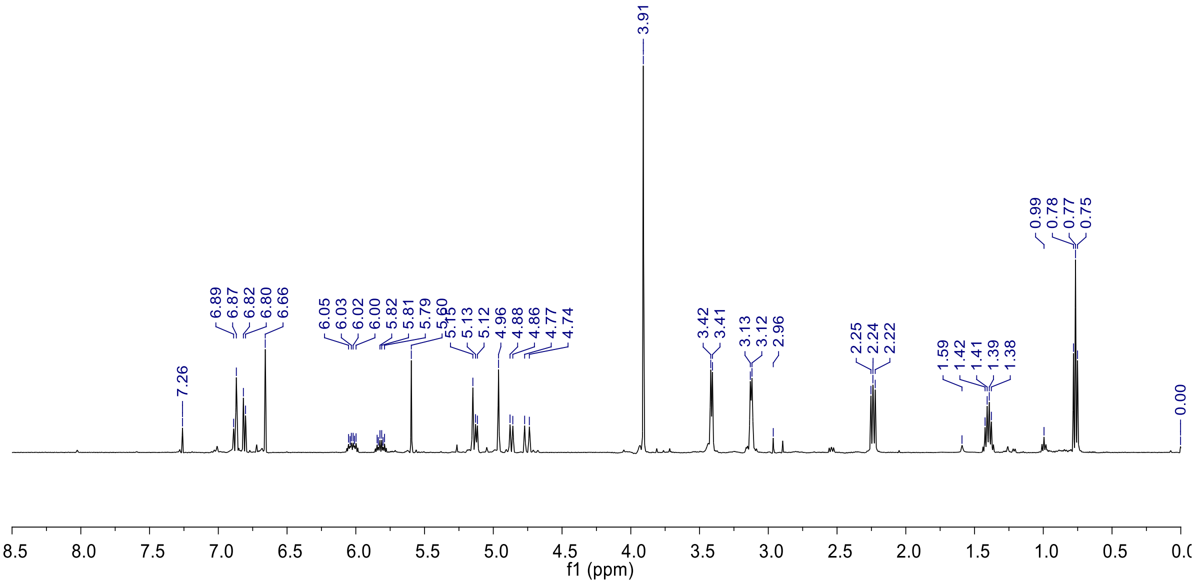

Figure S38. ^{1}H NMR spectrum (500 MHz, $CDCl_3$) of **14a**.

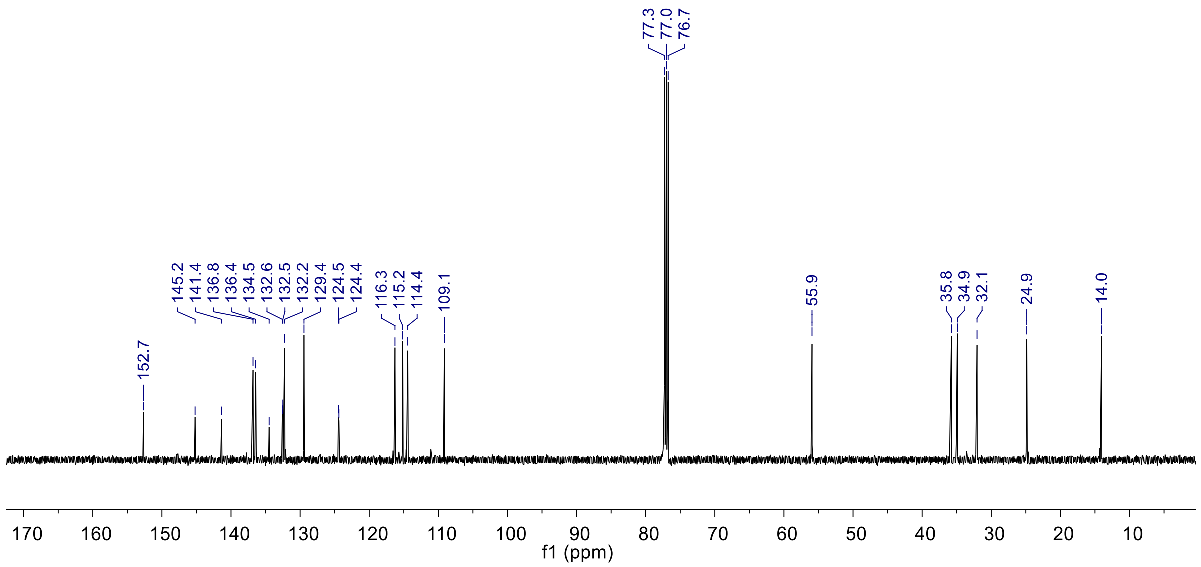

Figure S39. ${}^{13}C$ NMR spectrum (125 MHz, $CDCl_3$) of **14a**.

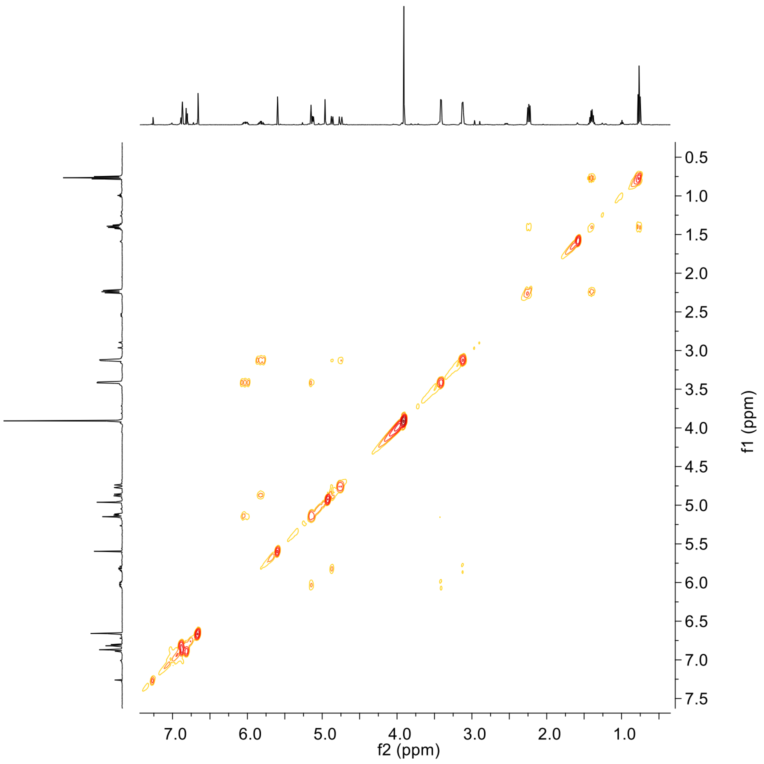

Figure S40. gCOSY spectrum of **14a**.

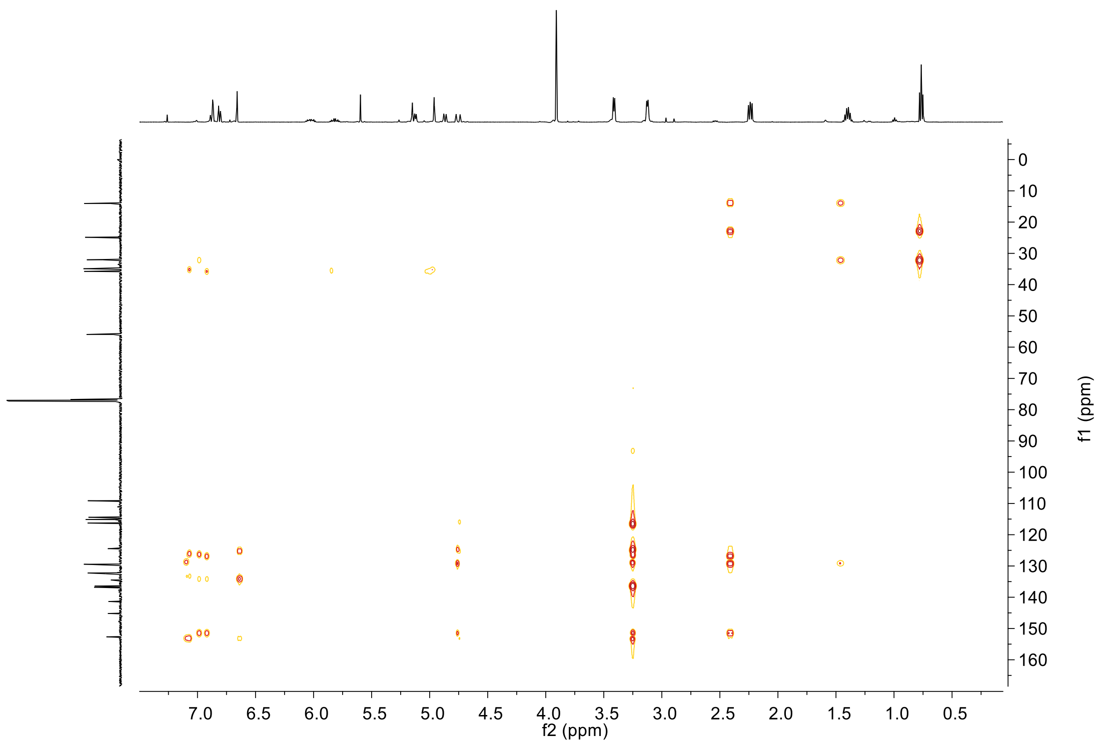

Figure S41. gHMBC spectrum of **14a**.

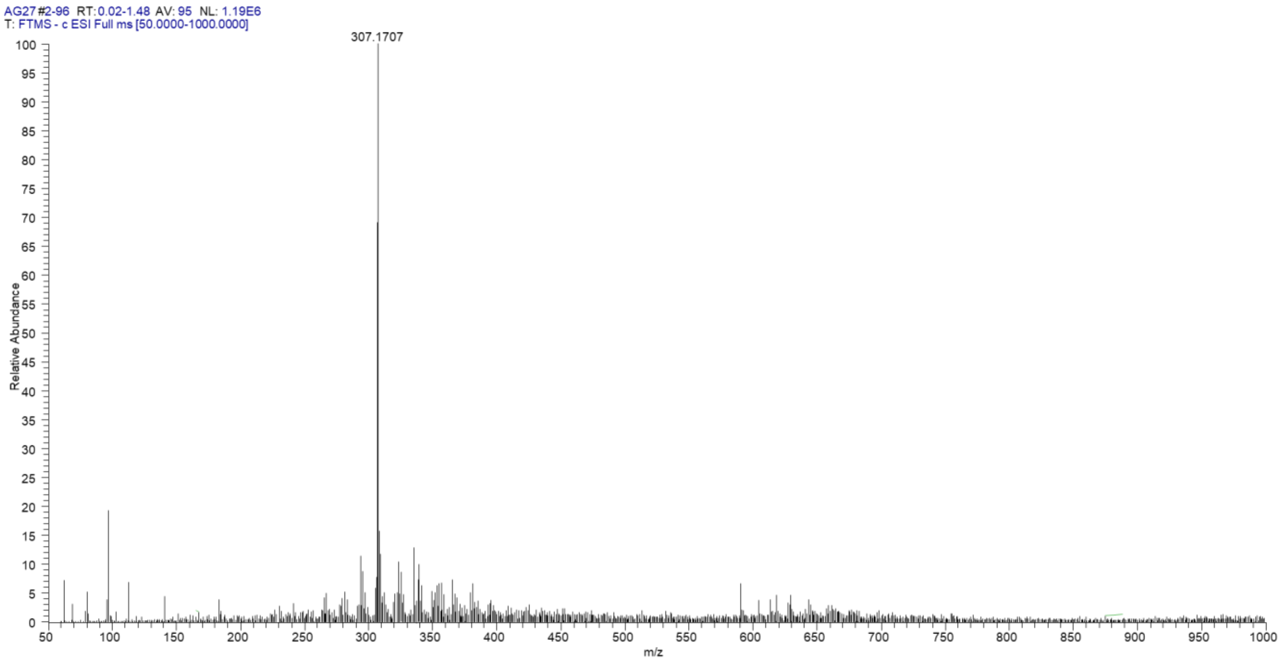

Figure S42. HRESIMS (-) spectrum of **14b**.

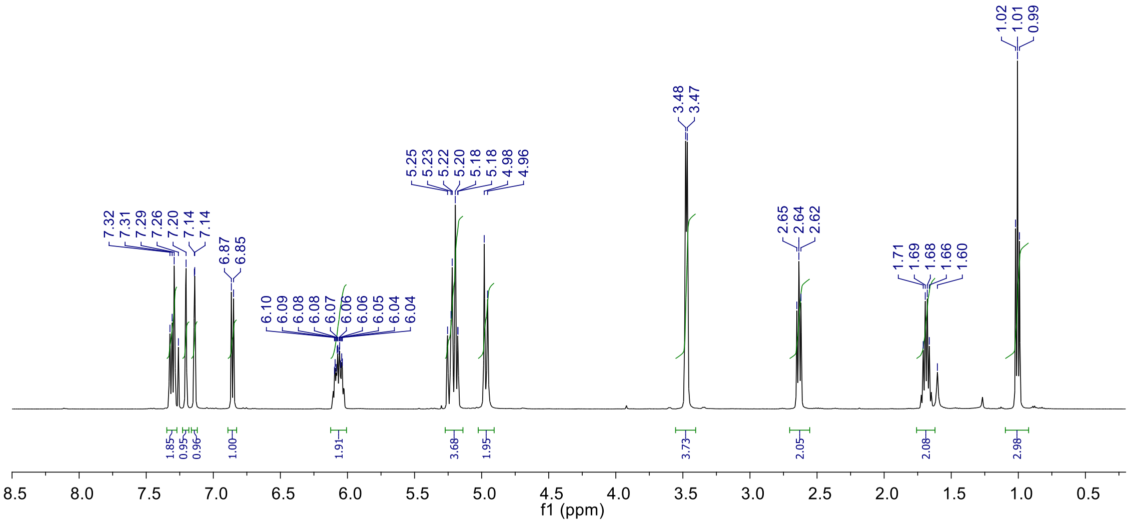

Figure S43. ^{1}H NMR spectrum (500 MHz, $CDCl_3$) of **14b**.

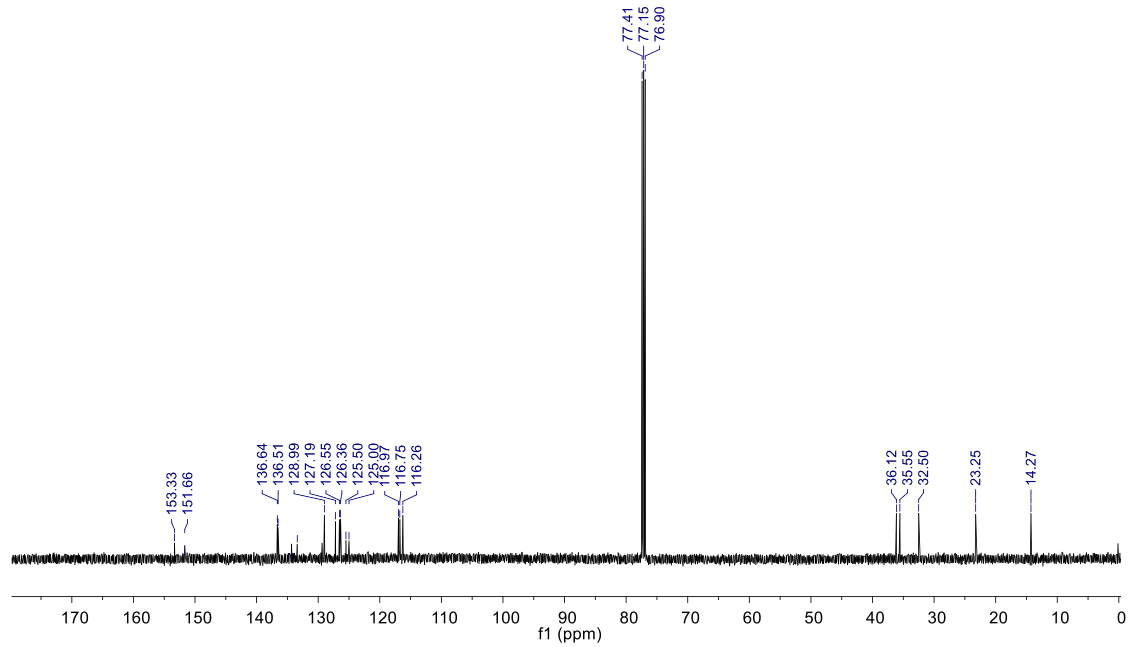

Figure S44. ^{13}C NMR spectrum (125 MHz, $CDCl_3$) of **14b**.

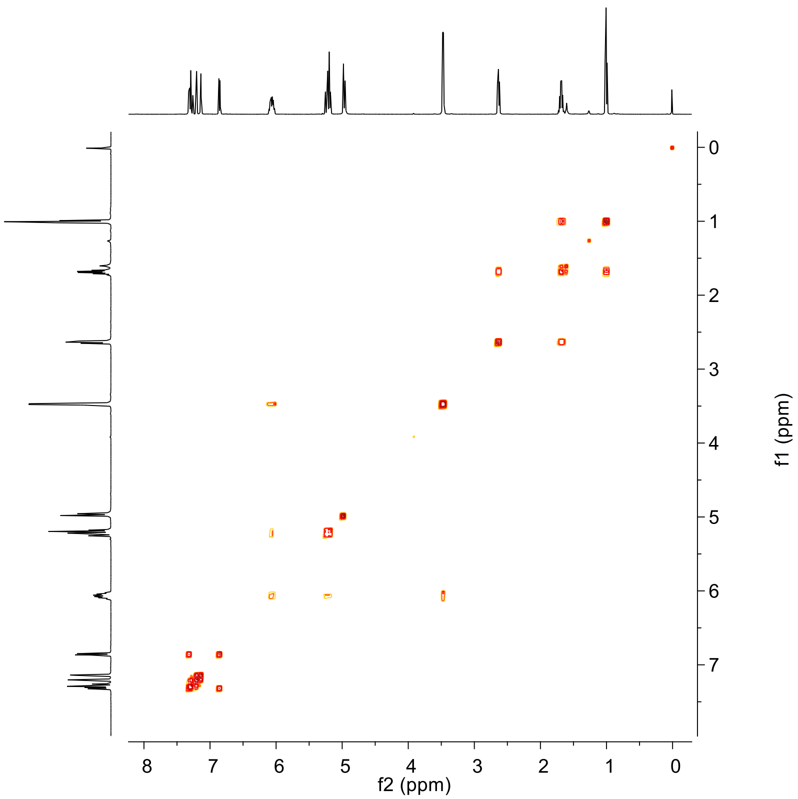

Figure S45. gCOSY spectrum of **14b**.

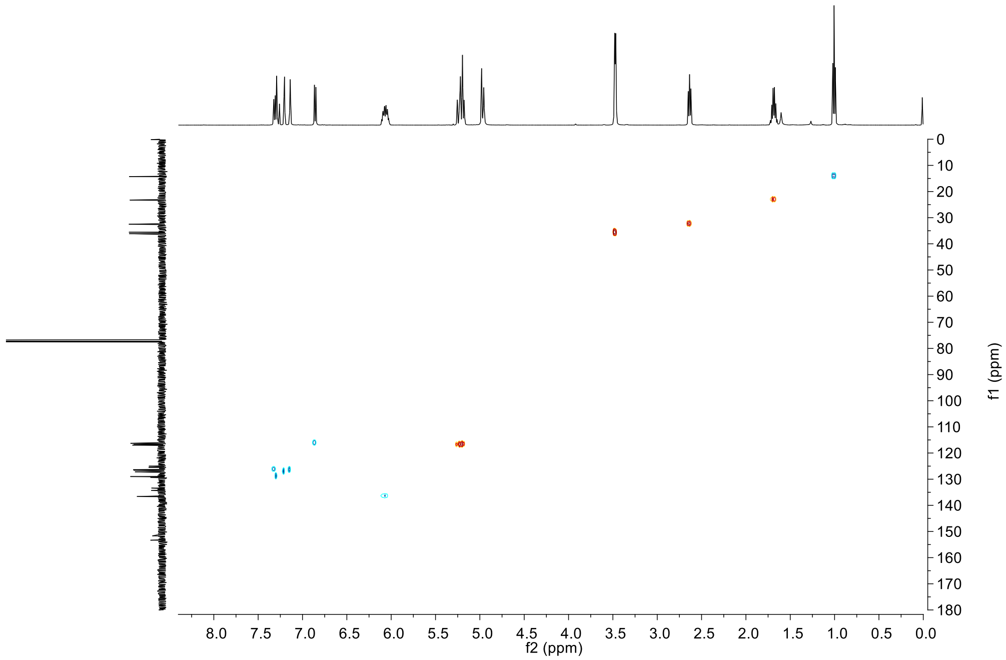

Figure S46. gHSQC spectrum of **14b**.

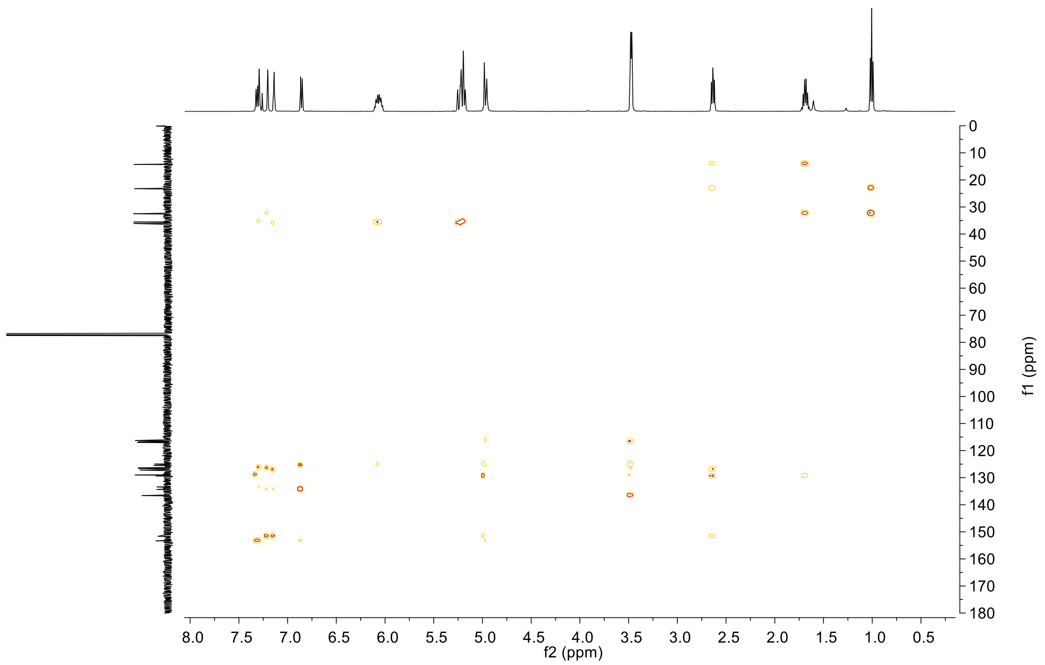

Figure S47. gHMBC spectrum of **14b**.

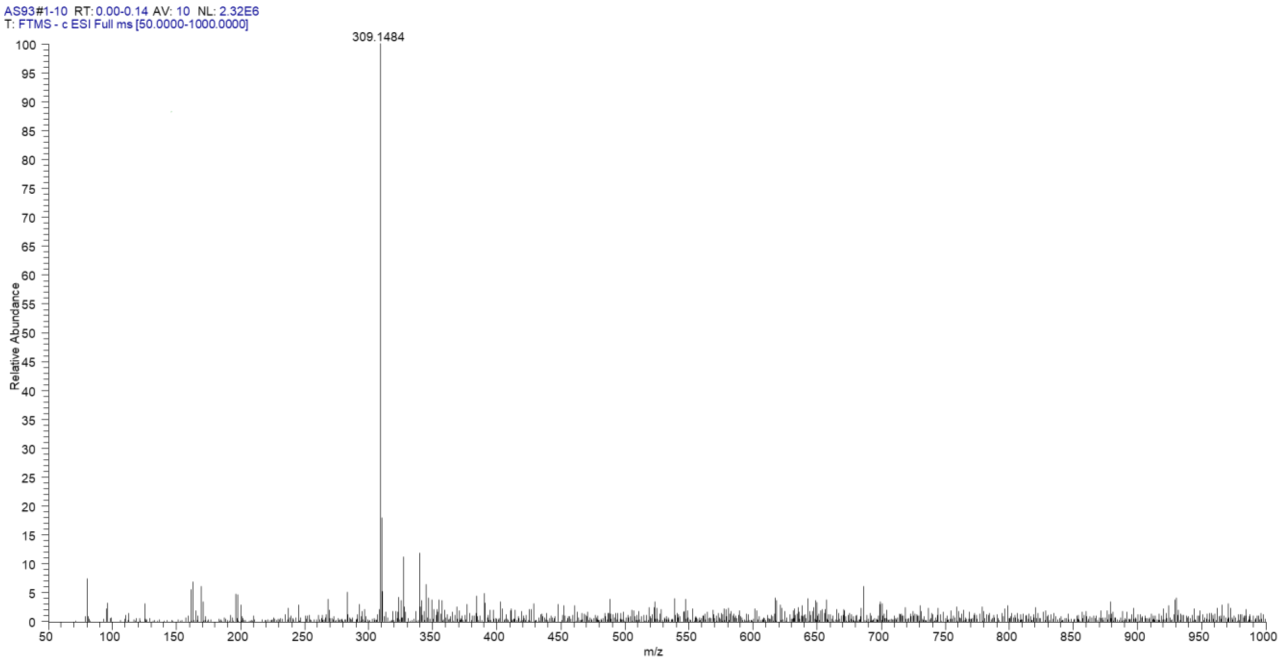

Figure S48. HRESIMS (-) spectrum of **14c**.

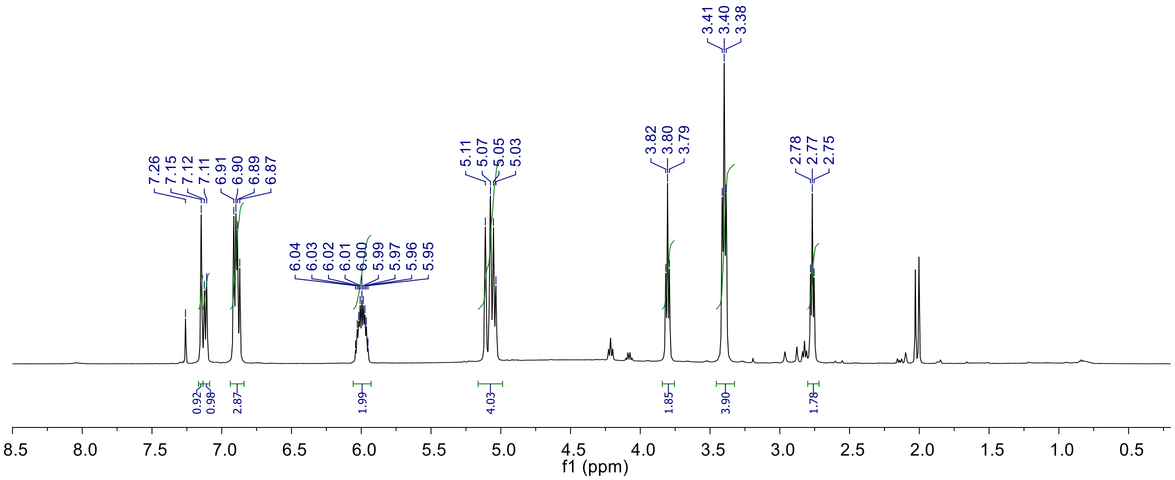

Figure S49. ^{1}H NMR spectrum (500 MHz, $(CD_3)_2CO$) of **14c**.

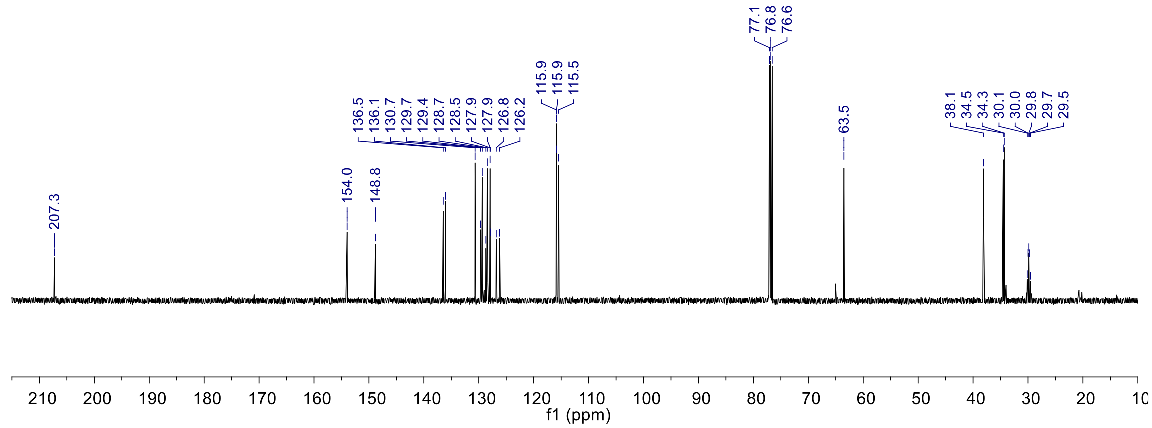

Figure S50. ^{13}C NMR spectrum (125 MHz, $(CD_3)_2CO$) of **14c**.

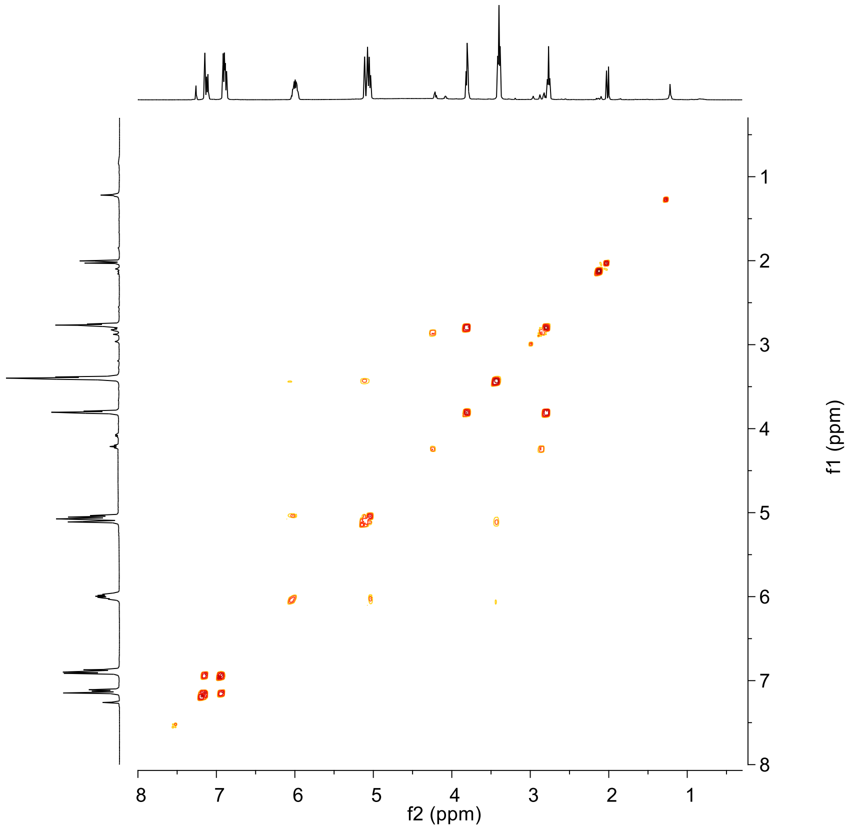

Figure S51. gCOSY spectrum of **14c**.

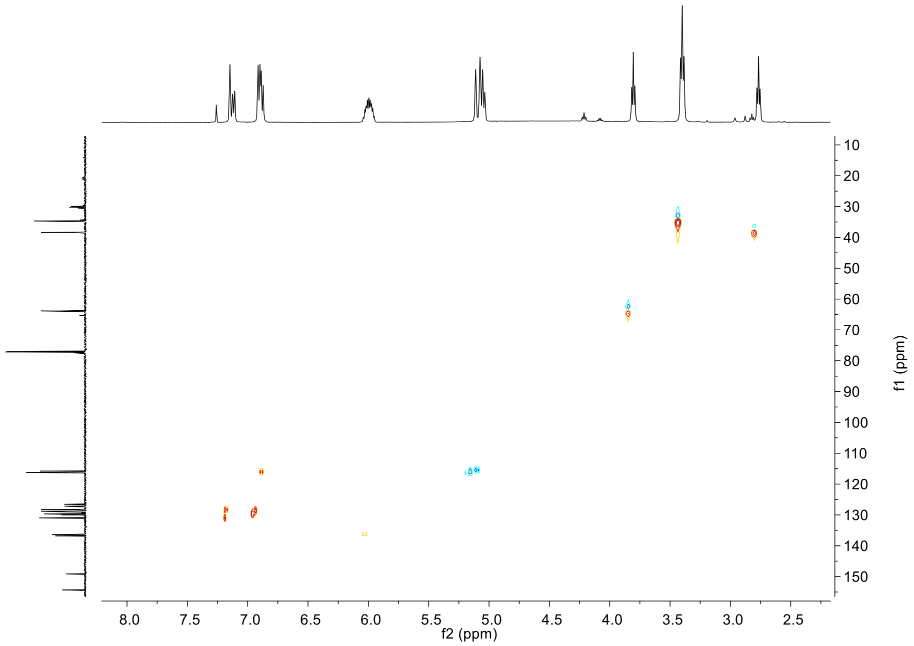

Figure S52. gHSQC spectrum of **14c**.

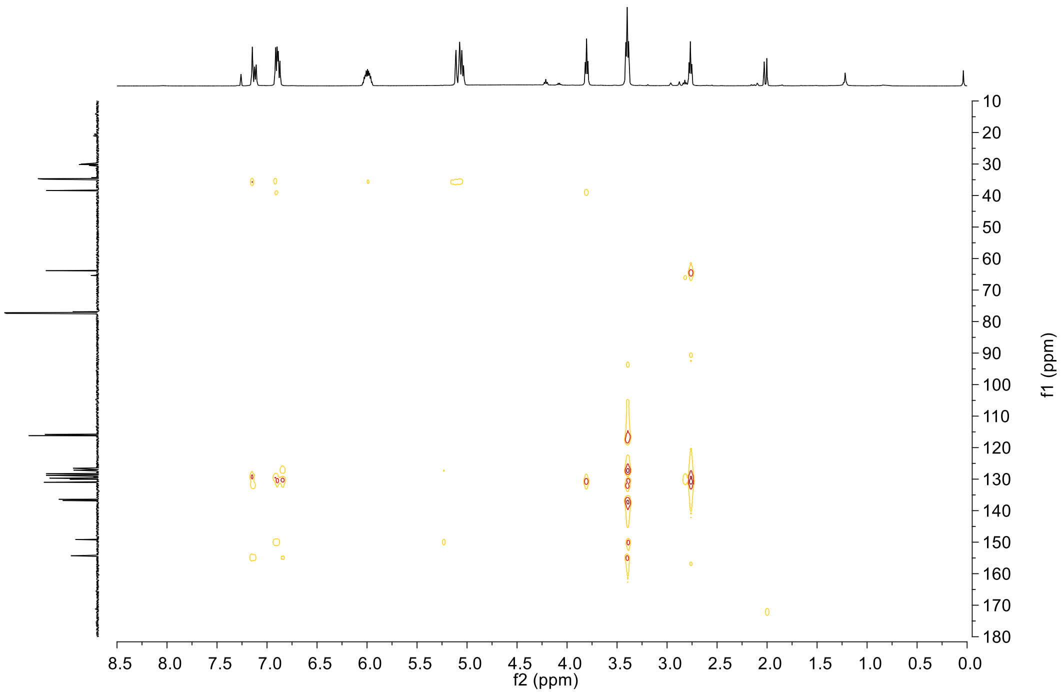

Figure S53. gHMBC spectrum of **14c**.

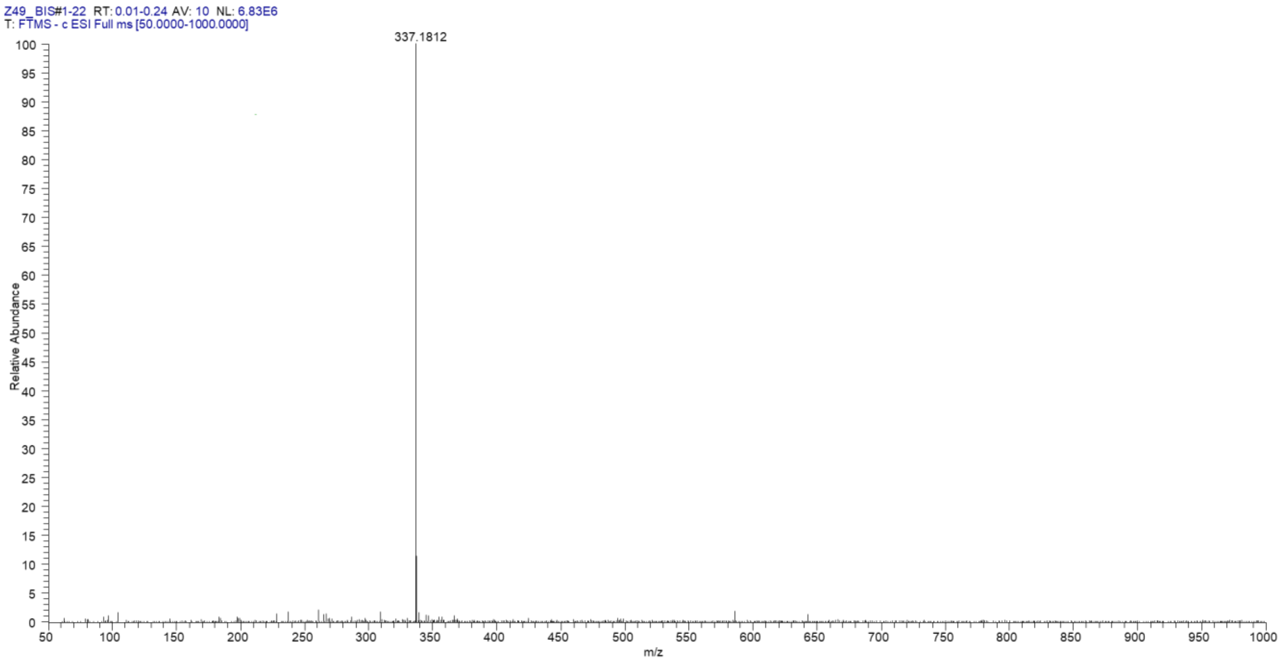

Figure S54. HRESIMS (-) spectrum of **15a**.

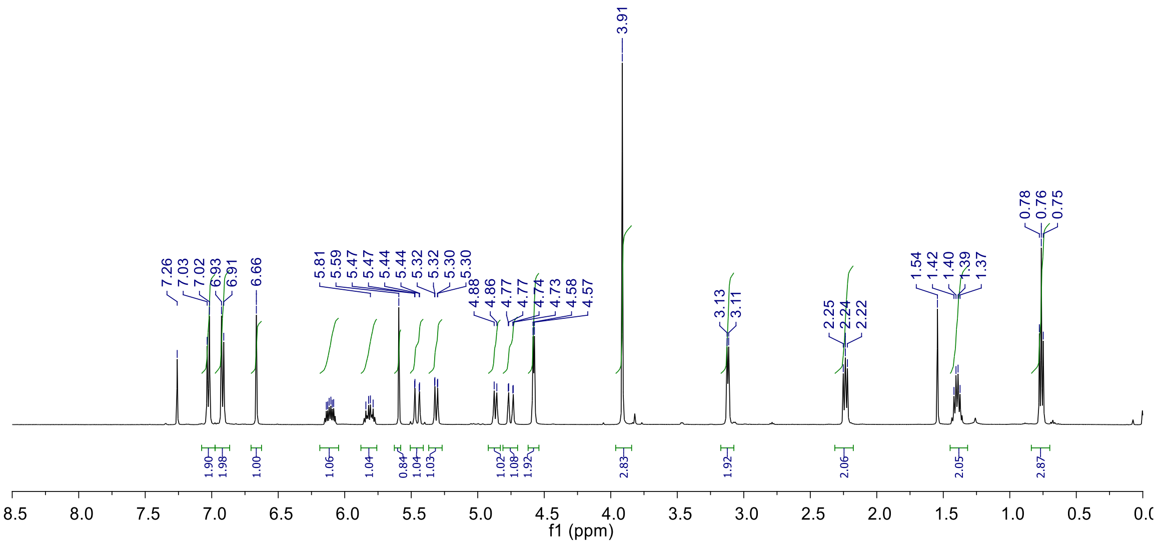

Figure S55. ^{1}H NMR spectrum (500 MHz, $CDCl_3$) of **15a**.

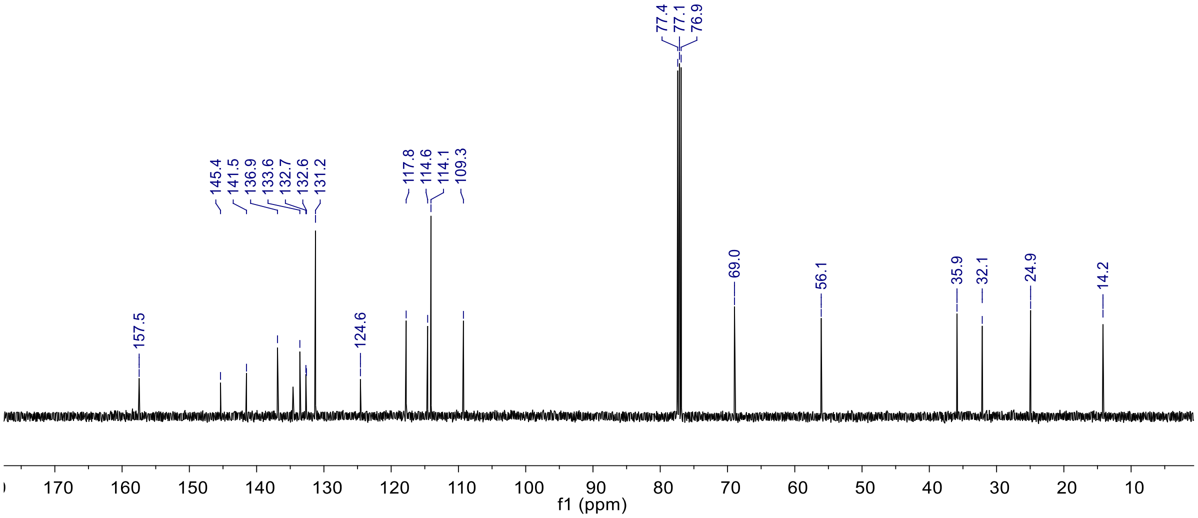

Figure S56. ^{13}C NMR spectrum (125 MHz, $CDCl_3$) of **15a**.

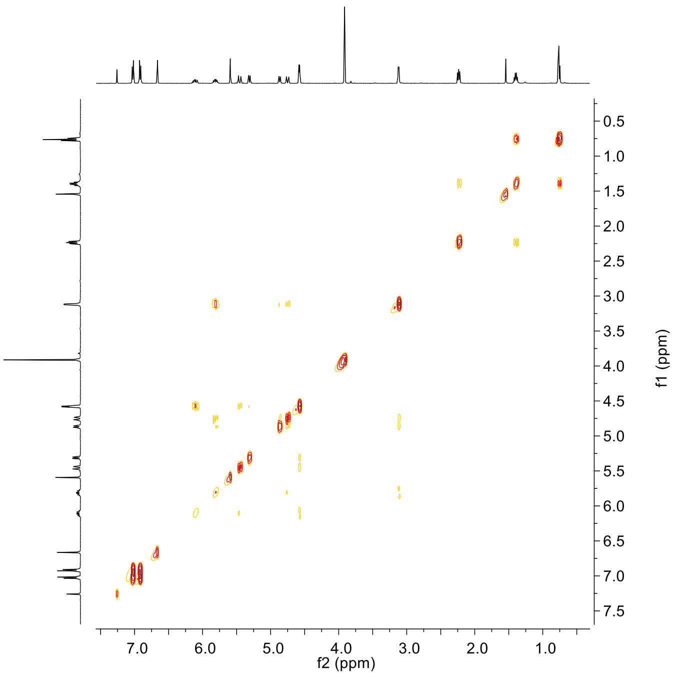

Figure S57. gCOSY spectrum of **15a**.

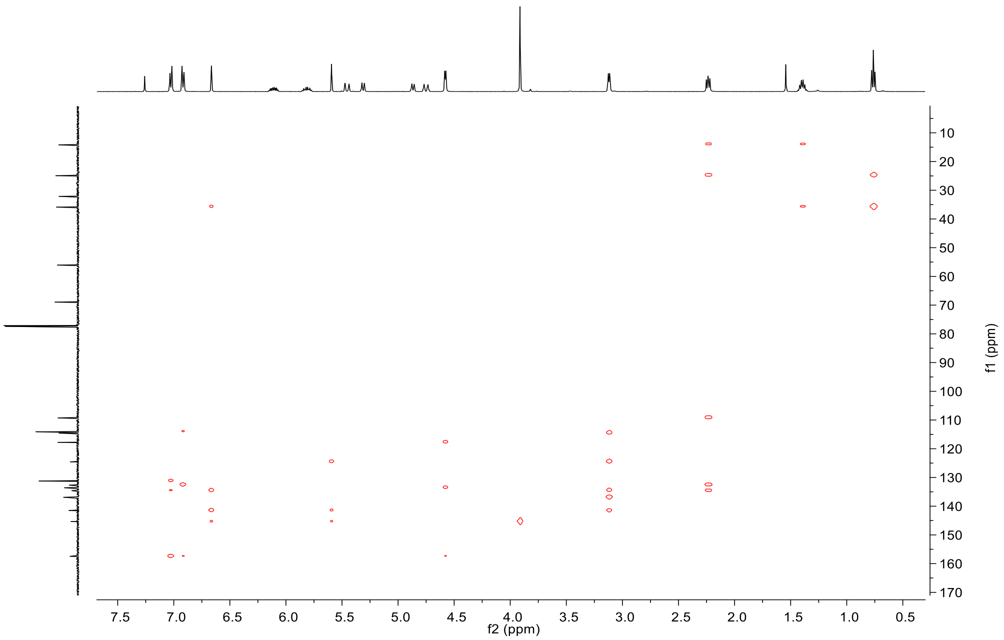

Figure S58. gHMBC spectrum of **15a**.
